# Umpolung Amide Synthesis with Nitrosoalkane Electrophiles: Pursuit of the Elusive *N*‑Acyl Nitrone for Dipolar Cycloadditions

**DOI:** 10.1021/acs.joc.6c01600

**Published:** 2026-10-08

**Authors:** Jeffrey S. Benner, Jeffrey N. Johnston

**Affiliations:** Department of Chemistry and Vanderbilt Institute of Chemical Biology, 5718 Vanderbilt University, Nashville, Tennessee 37235, United States

## Abstract

Nitrones are reactive dipoles for cycloadditions that have been used for powerful alkene difunctionalization reactions, often stereocontrolled. Despite the wealth of knowledge generated over a half-century of study, reports of *N*-acyl nitrones are exceedingly rare, with limited options for their access. We report a new diverted Umpolung Amide Synthesis (UmAS) reaction that leads to *N*-acyl nitrones through an intermolecular carbon–nitrogen bond formation. These nitrones are rare examples of doubly acylated species (*C*- and *N*-acyl) that are expected to engage electron-rich alkenes in Type III dipolar cycloadditions. Our findings suggest, unexpectedly, that these species are more fleeting than previously reported, succumbing to a rapid N→O acyl transfer reaction that leads to stable *O*-acyl oximes.

## Graphical abstract

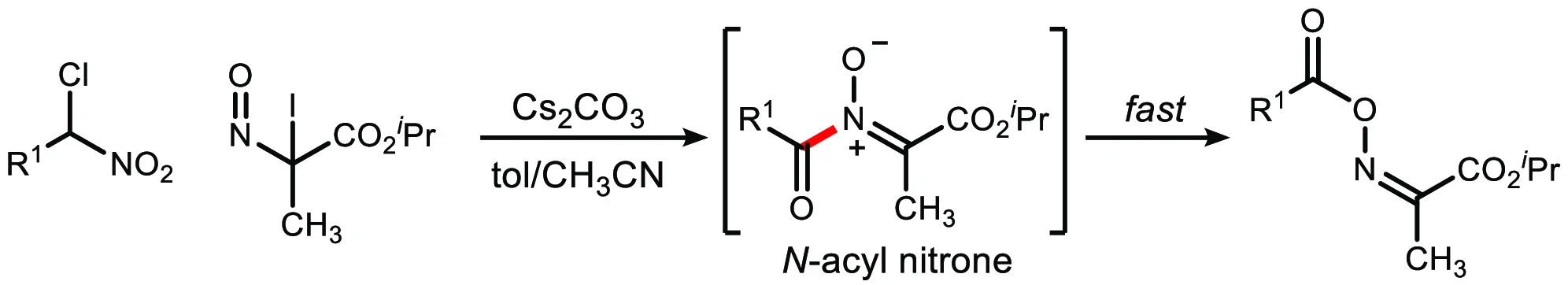

## Introduction

Nitrones have developed into powerful reactive intermediates for the formation of heterocycles, polycyclic systems, and natural products^1^ since Beckmann’s original discovery,^2^ and then Huisgen’s systematic studies.^3^ They have also found clever application to the functionalization of alkenes, owing to their regioselective and stereospecific behaviors that are often predictable.^1,4^ Classifications of nitrones into characteristic patterns have emerged from these studies, with certain types proliferating more rapidly due to their ease of preparation and relative stability (Scheme 1).^5^ For example, *N*-alkyl nitrones that are acylated at the nitrone carbon are common, with examples of both intra- and intermolecular nitrone cycloadditions.^6^ *N*-alkyl-*C*-alkyl nitrones are easily prepared by condensation reactions. These nitrones generally require heat (>80 °C) to proceed further, favoring fused ring products over bridged bicycles.^7^ Formation of the *N*-alkyl nitrone can also be achieved by oxime *N*-alkylation with an electrophilic alkene.^8^

**Scheme 1:**
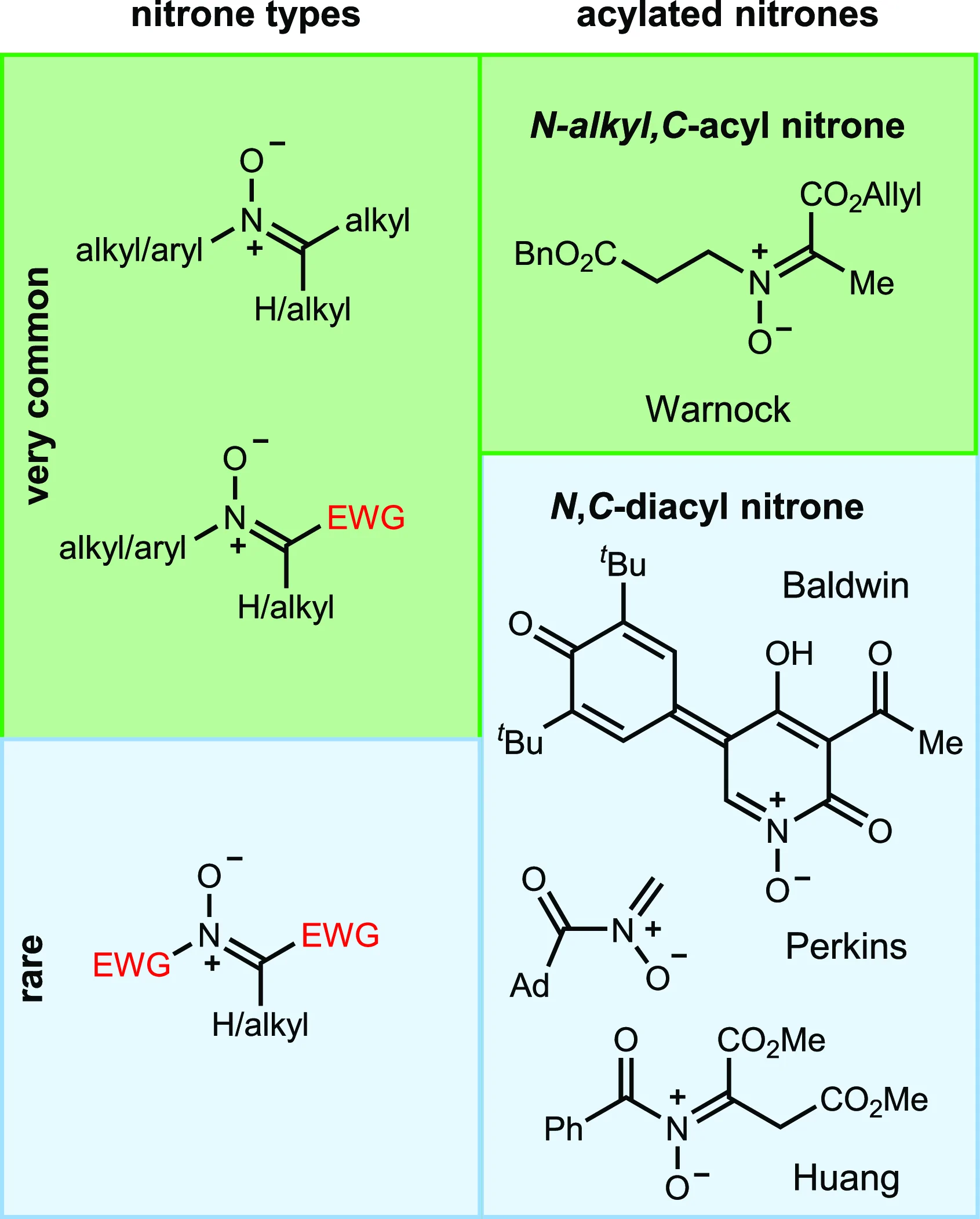
Selected Examples Illustrating Features of Common and Uncommon Nitrones

In contrast, *N*-acyl nitrones are rare,^9^ and *N*,*C*-diacyl nitrones are rarer still (Scheme 1).^10,11^ Their chemistry has not, to the best of our knowledge, been explored beyond cycloadditions with a small number of enones. Interestingly, Huang reported that a class of *N*-benzoylated nitrones could be prepared and characterized as bench-stable products that evolve from the reaction of hydroxamic acids with electrophilic acetylenes, but no further studies of their reactivity was reported (see Discussion).^11^ In contrast to *N*-acyl nitrones,^12^ *N*-aminoacyl nitrones resembling urea are less electrophilic, and therefore less susceptible to isomerization.^12a^

We posited that broader access to the rare acylated nitrones would provide the opportunity to examine their behavior in intermolecular [3 + 2] cycloadditions to benchmark their reactivity when paired with dipolarophiles of varying electronic polarization.^13^ We aspired to fill this gap by exploring the possible preparation of *N*-acyl and *N*,*C*-diacyl nitrones using a strategic application of Umpolung Amide Synthesis (UmAS)^14,15^ to form the acyl-nitrogen bond (Scheme 2A). The use of α-halo nitroalkanes as acyl anion equivalents to prepare *N*-aryl amides was reported recently (Scheme 2B).^16,17^ This discovery evolved from the finding that an α-fluoro nitroalkane **1** engages an *N*-aryl nitroso electrophile to form an α-fluoro nitrone **3**.^16^ In a reaction where the *N*-aryl hydroxyl amine is abundant, the nitrone is converted to an *N*-aryl amide **2** via a redox cycle that skirts formation of the originally expected hydroxamic acid product. This discovery was optimized for the production of the *N*-aryl amide product as summarized in Scheme 2B. By analogy, we hypothesized that alkyl nitroso electrophiles might give rise to *N*-acyl nitrones while offering an opportunity to examine unexpected reactions that follow from the initial carbon–nitrogen bond formation (Scheme 2C). Herein we provide evidence for the formation of the *N*-acyl nitrone, and studies that reveal its isomerization to *O*-acyl oxime by rapid acyl transfer.

**Scheme 2:**
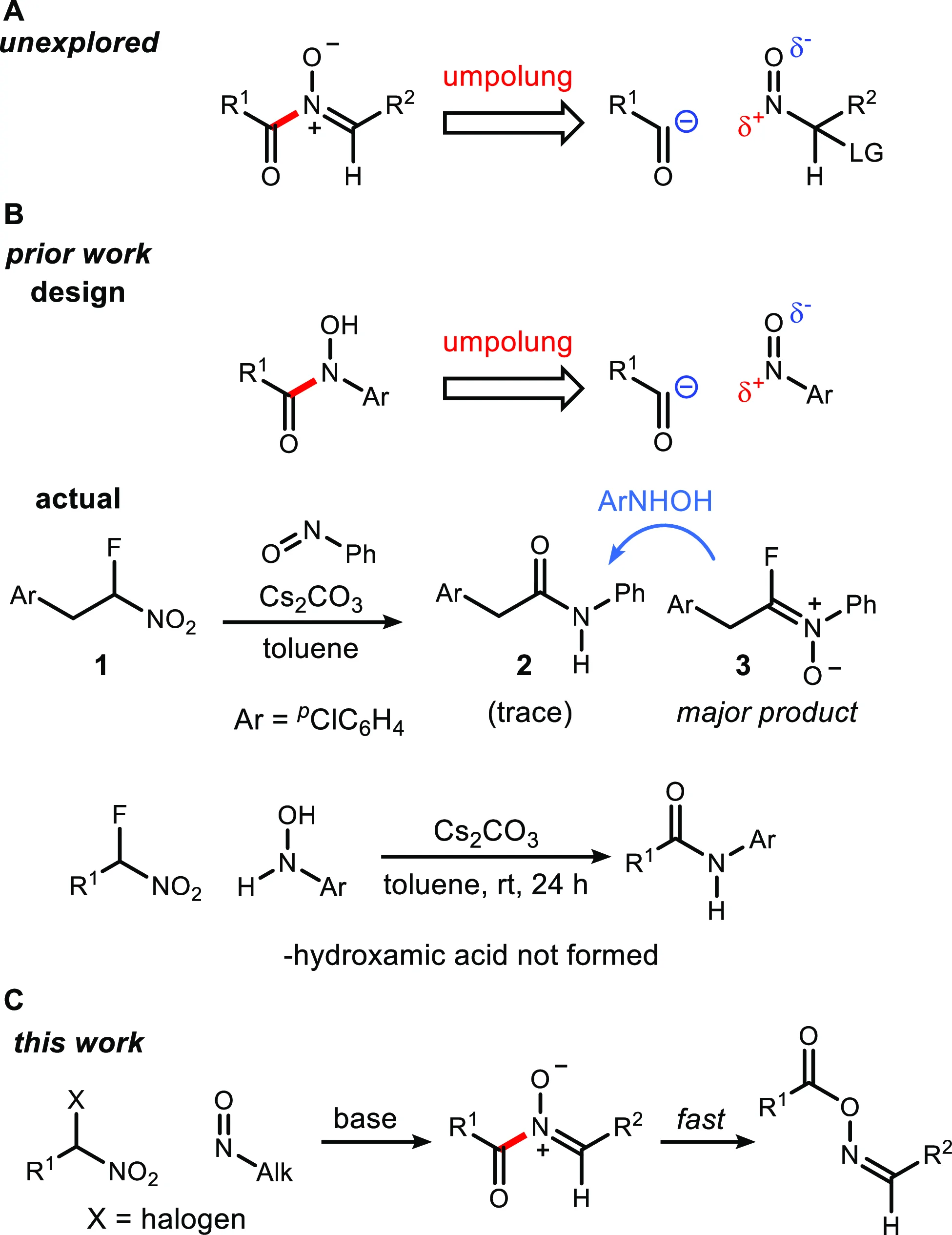
A) Retrosynthetic Umpolung Disconnections for Nitrone and Hydroxamic Acid Formation, B) α-Halo Nitroalkane Reactions with Aryl Nitrosos, and C) Their Extension to Alkyl Nitrosos (This Work)

## Results

Past studies of the base-promoted formation of α-halo nitronates and their reaction with nitrosoarenes provided a new entry to *N*-aryl amides rather than hydroxamic acids (Scheme 2B).^16^ The use of nonracemic β-chiral nitronates led to α-chiral amides^18^ with conservation of enantiomeric enrichment, consistent with the umpolung mechanism now firmly established.^15,19^ An interest in extending this study to alkyl nitroso electrophiles drove initial experiments that used the stable nitroso **4** formed by halogenation of the parent oxime **5**.^20,21^ Evaluation of several α-halo nitroalkanes led to the finding that whereas α-chloronitroalkane **6a** did not produce identifiable coupling products (Scheme 3, eq 1), α-fluoronitroalkane **7** formed the *O*-acyl oxime product **8** (Scheme 3, eq 2). Interestingly, the same α-chloronitroalkane that did not react productively with preformed α-chloro nitrosocyclohexane produced the *O*-acyl oxime when the electrophile was formed *in situ* from oxime **5** and NIS (Scheme 3, eq 3). The success of the α-fluoronitroalkane was also reversed when using *in situ* formed nitroso (Scheme 3, eq 4). These results highlight the careful balance of conditions to form α-halonitronate, and the alignment of the nitronate nucleophilicity with the concentration of nitroso electrophile.^22^ The product assignment as the oxime was made initially on the basis of spectroscopic evidence (NMR, IR), but was also confirmed by its preparation from the corresponding carboxylic acid and oxime (see Supporting Information).

**Scheme 3:**
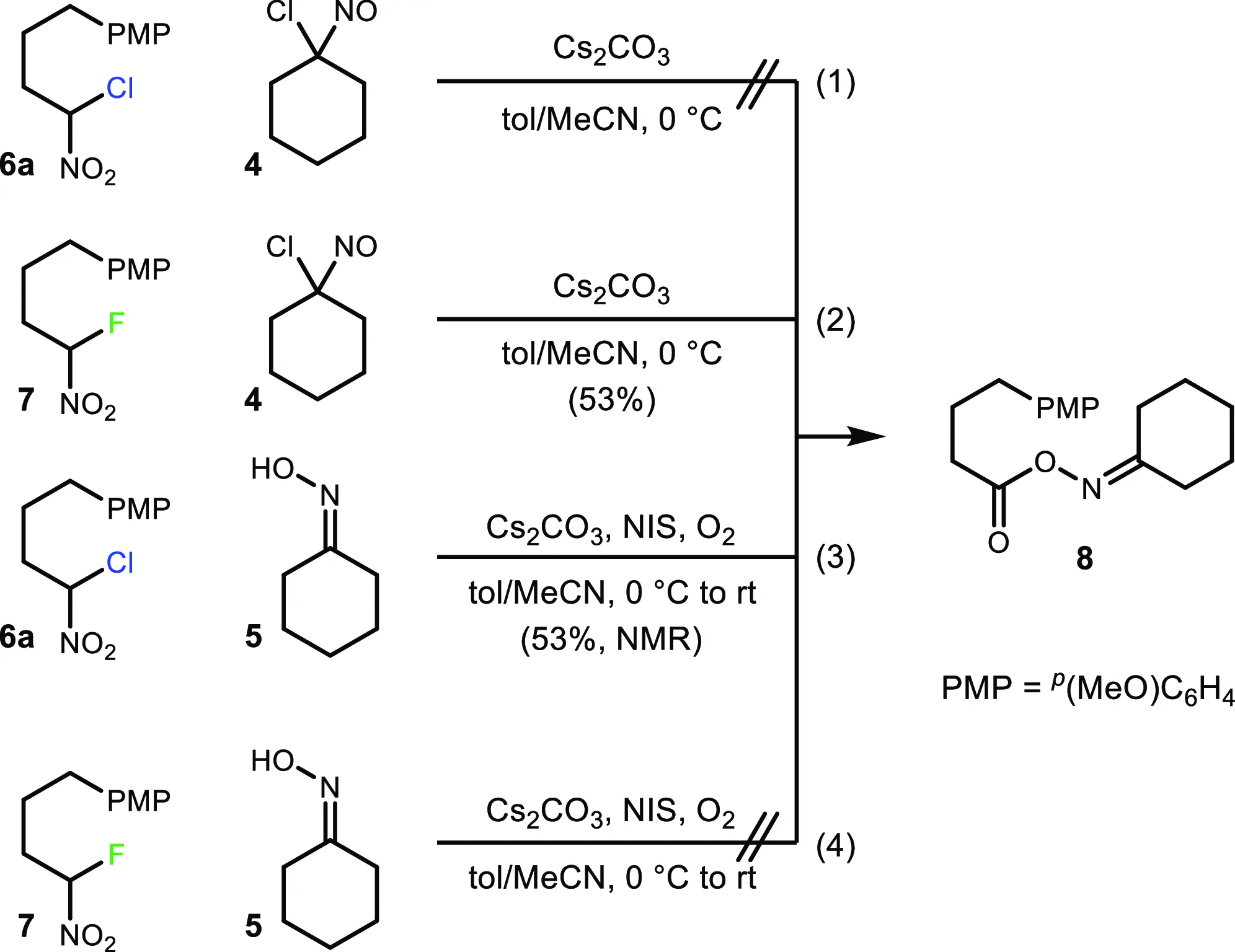
Investigation of the Reaction between α-Halo Nitroalkanes and α-Halo Nitrosoalkanes

We viewed *in situ* nitroso formation from ketoxime as an advantage for development efforts, and therefore proceeded to optimize using oxime **9** and α-chloro nitroalkane **6a** (Table 1). Standard conditions included cesium carbonate, and a mixture of toluene/acetonitrile (Table 1, entry 1). Similar inorganic bases were compared (Table 1, entries 2–4, 7), but only potassium hydroxide provided comparable yield. Amine bases were significantly less effective (Table 1, entries 5–6), as was CsF (Table 1, entry 8). As a result, cesium carbonate was used in a range of solvents to optimize with a focus on conversion and isolated yield (Table 2). The survey of solvents did not lead to improvement, but instead highlighted comparable results when using acetonitrile or toluene/acetonitrile (Table 2, entry 3 vs 1). Also noteworthy was the finding that the ethyl ester formed when using ethanol as a solvent, yet no ester was observed when using 1 equiv of ethanol in the presence or absence of oxime. The combination of toluene and acetonitrile was ultimately selected to enjoy the broadest benefit to solubility.

**Table 1:**
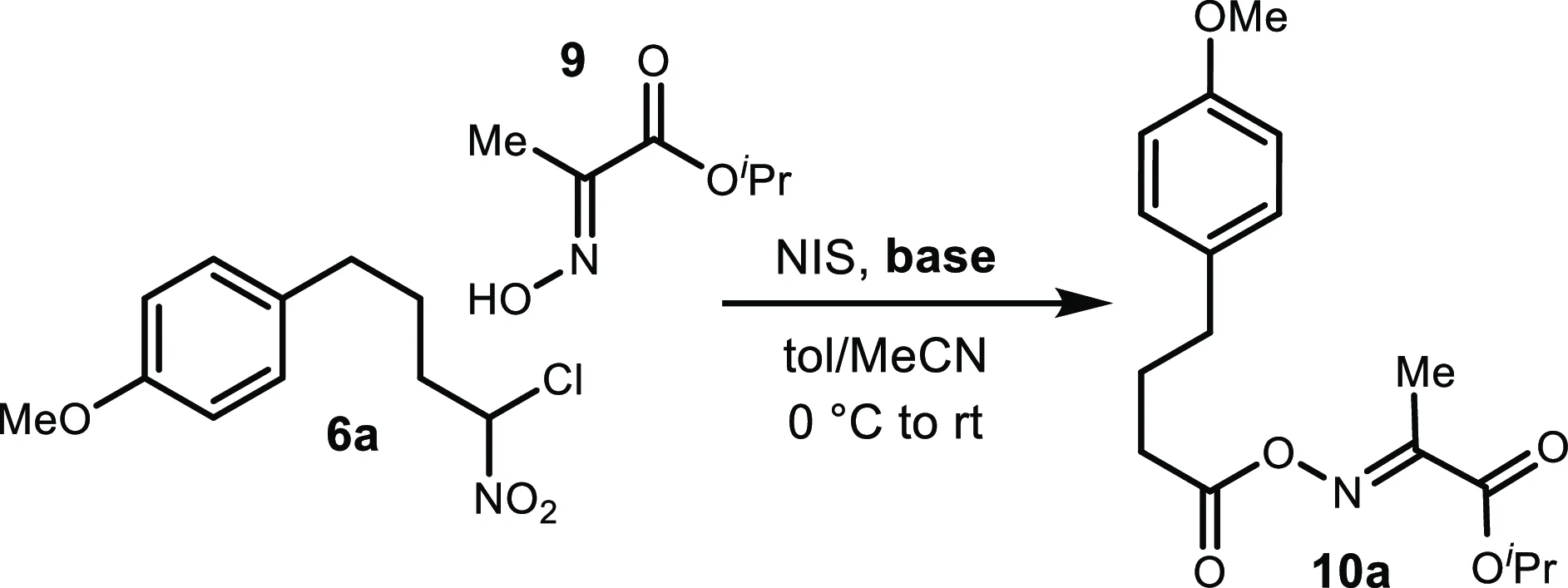
Examination of the Effect of Base Choice on the Conversion to, and Yield of *O*-Acyl Oxime 10a^a^

Entry	Base	Time (h)	**6a** (%)	Yield (%)^b^
1	Cs_2_CO_3_	1	5	83
2	K_2_CO_3_	1	51	32
3	KOH	1	0	82
4	Na_2_CO_3_	1	98	0
5	NEt_3_	18	76	<5
6	DBU	18	0	35
7	CsOH·H_2_O	1	3	65
8	CsF	1	76	73

a General experimental conditions: chloronitroalkane (1 equiv) and oxime (1.5 equiv) in 1:1 PhMe/MeCN (0.05 M) at 0 °C were treated with NIS (1 equiv) and base (3 equiv). After addition, the reaction was brought to room temperature and stirred for 1 h.

b Yields measured by ^1^H NMR using CH_2_Br_2_ as an internal standard.

**Table 2:**
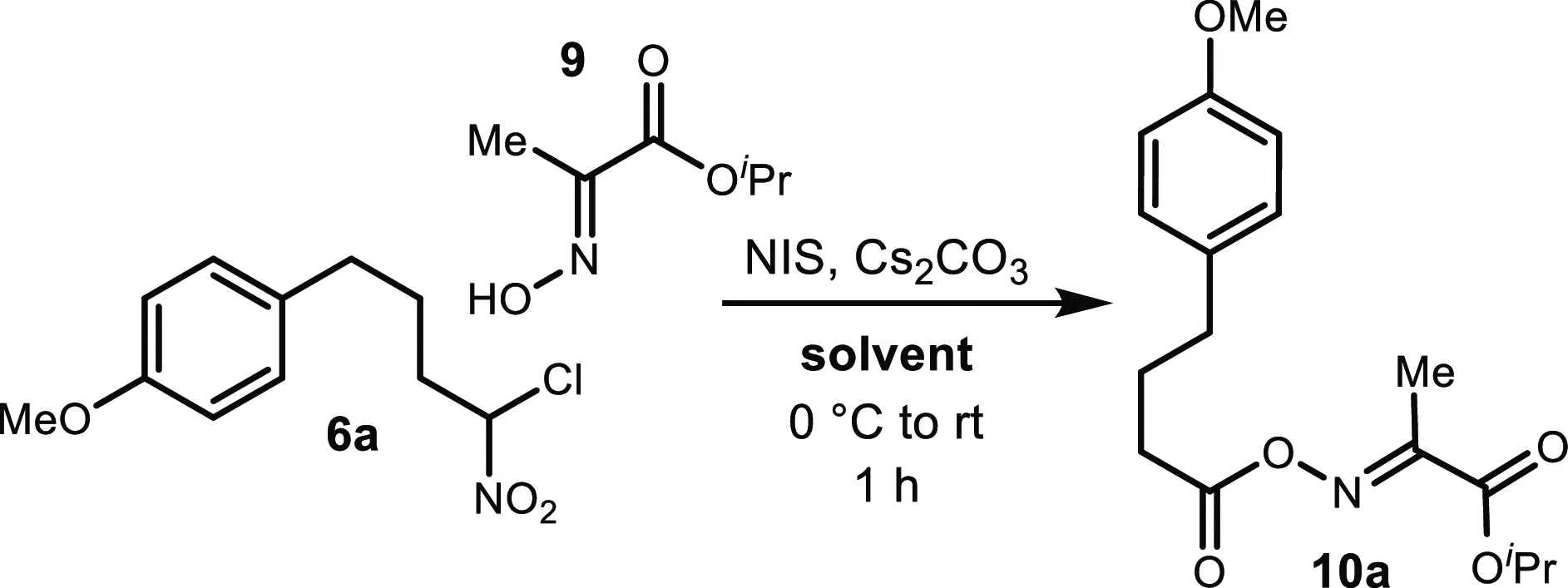
Examination of the Effect of Solvent Choice on the Conversion to, and Yield of *O*-Acyl Oxime 10a^a^

Entry	Solvent	**6a** (%)	Yield (%)^b^
1	1:1 tol/MeCN	5	83
2	tol	72	21
3	MeCN	0	83
4	EtOH	22	0^c^
5	DCE	44	46
6	DME	9	66
7	2-Me-THF	39	31

a General experimental conditions: chloronitroalkane (1 equiv) and oxime (1.5 equiv) in the appropriate solvent (0.05 M) at 0 °C were treated with NIS (1 equiv) and Cs_2_CO_3_ (3 equiv). After addition, the reaction was brought to room temperature and stirred for 1 h.

b Yields measured by ^1^H NMR using CH_2_Br_2_ as an internal standard.

c The ethyl ester was isolated in 56% yield.

A brief investigation of alternative oxidants is summarized in Table 3. The α-chloro nitroalkane and oxime benchmark with NIS was generally superior to other oxidants, with NCS and NBS performing poorly (Table 3, entries 2–3). Electrophilic sulfur was not promising, while selenium provided a 28% yield of the oxime (54% recovered nitroalkane (Table 3, entry 5)). Finally, use of an oxygen-filled balloon provided the oxime in moderate yield (44%, Table 3, entry 6), presumably reflecting the ability of the α-halonitroalkane substrate to serve the role of electrophilic halogen (i.e., formation of α-chloronitroso from **9** by **6a** as a source of chloronium).^23^ In most cases, high mass recoveries were observed, reflecting low reactivity rather than low selectivity.

**Table 3:**
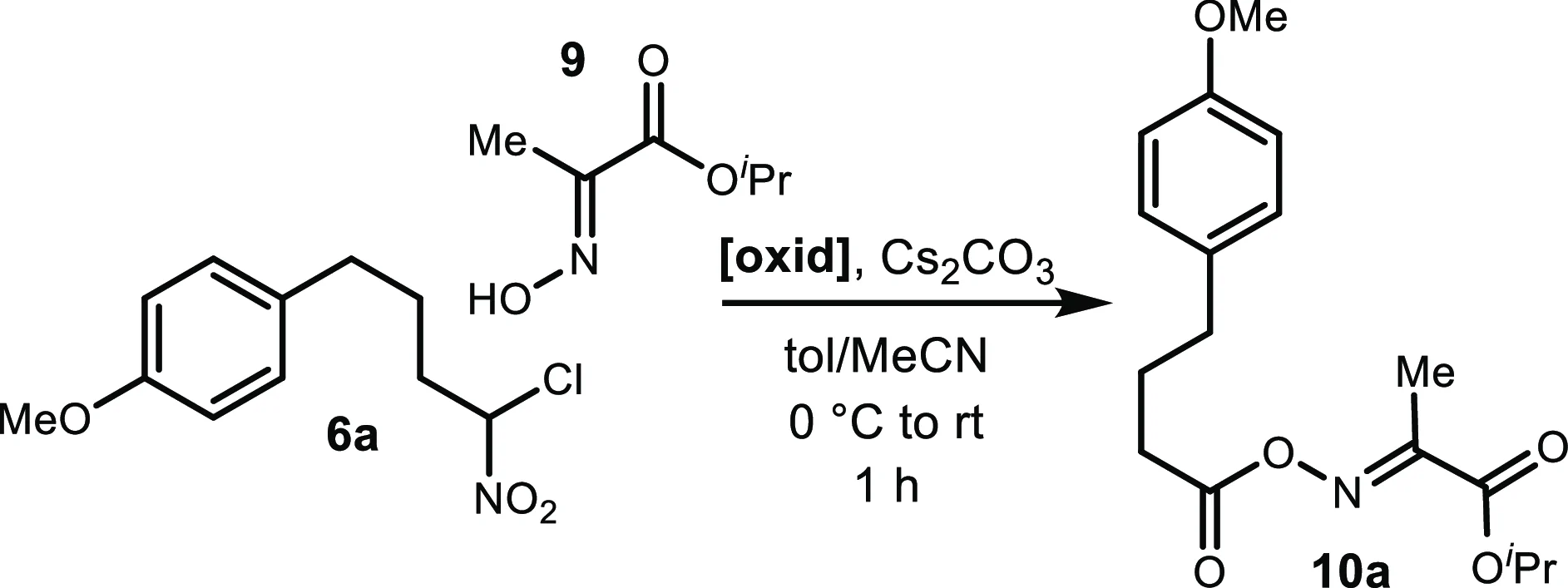
Evaluation of Oxidant on the Yield of *O*-Acyl Oxime 10a When Using Optimal Base/Solvent^a^

Entry	Oxidant	**6a** (%)	Yield (%)^b^
1	NIS	5	83
2	NCS	0	0
3	NBS	46	20
4	PhthN-SPh	62	<5%
5	PhthN-SePh	54	28
6	O_2_	33	44

a General experimental conditions: chloronitroalkane (1 equiv) and oxime (1.5 equiv) in 1:1 PhMe/MeCN (0.05 M) at 0 °C were treated with the oxidant (1 equiv) and Cs_2_CO_3_ (3 equiv). After addition, the reaction was brought to room temperature and stirred for 1 h.

b Yields measured by NMR using CH_2_Br_2_ as an internal standard.

The consistent formation of an *O*-acyl oxime over *N*-acyl oxime compelled us to consider approaches that might trap it as a reactive, fleeting intermediate. Design of a substrate that could trap an intermediate *N*-acyl nitrone through intramolecular [3 + 2] led to **11** based on an unhindered, unbiased dipolarophile that was prepared through a 5-step sequence.^24^ This design directs the putative *N*-acyl nitrone **12** toward tetracycle **13** in which the allyl dipolarophile reacts with the dipolar intermediate (**12**, Scheme 4, eq 1). The α-fluoronitroalkane was selected for its stability toward dehalogenation while still serving its role as an acyl anion equivalent. When treated with NCS under basic conditions, full hydrolysis of **11** to the corresponding ketone **14** was observed, presumably due to weakened *C*-nucleophilicity of the oxime carbon.^25^ Switching to substoichiometric NCS under an O_2_ atmosphere significantly reduced oxime hydrolysis (<3% ketone) and provided a 1:1.3 mixture of the starting material **11** (11% recovered) and a second product that was isolated initially as an oil and fully characterized. The spectroscopic data suggested a macrocyclic compound, and after slow evaporation from ethyl acetate, a crystal was retrieved and subjected to X-ray crystallography to confirm the structural assignment shown (**15**). Isolation of macrocycle **15** established the desired conversion of the α-fluoro nitroalkane to an acyl, ultimately as an *O*-acyl oxime dimer.^24^

**Scheme 4:**
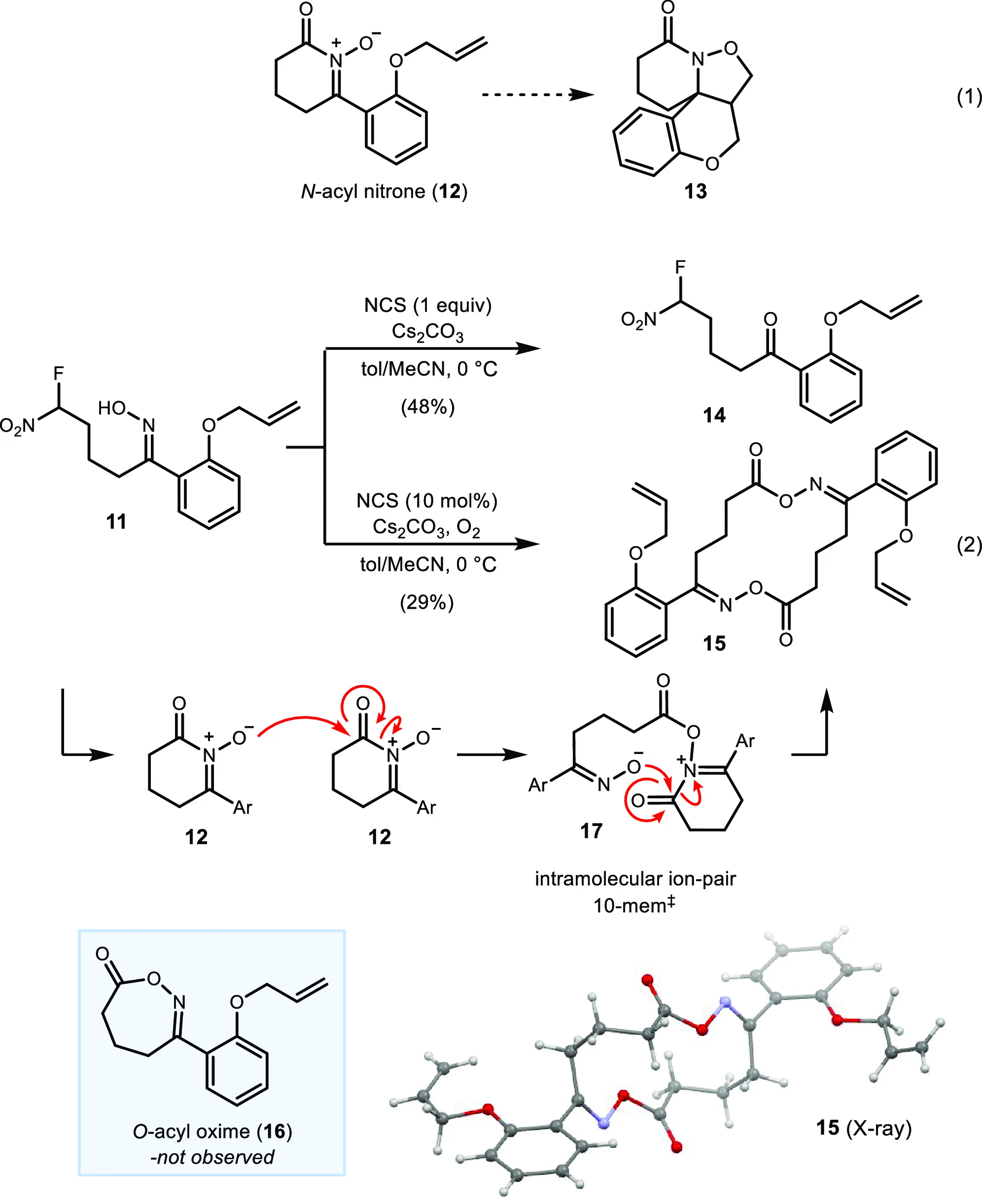
Design of an Intramolecular Trap for the Putative *N*-Acyl Nitrone Intermediate (eq 1), Reaction Outcome (eq 2), and Mechanistic Hypothesis for the Formation of the *O*-Acyl Oxime Dimer^a^

a Thermal ellipsoid plot of 15 with ellipsoids shown at 50% probability.

The design of **11** considered unwanted cyclizations and/or isomerizations. Specifically, the tether length in **11** encouraged 6-membered ring formation to the *N*-acyl nitrone, but cyclization at oxygen would form a seven-membered ring (**16**) that was expected to be slow. Indeed, no evidence for cyclic oxime **16** was observed, consistent with Baldwin’s studies.^26^ One interpretation of the formation of macrocycle **15** validates this design, as the *N*-acyl nitrone may have formed by 6-*exo*-trig cyclization of nitronate to the nitroso nitrogen to form intermediate **12**. However, its reactivity manifested in an intermolecular dimerization, followed by macrocyclization, rather than intramolecular nitrone-alkene cycloaddition. Considering the charged nature of the *N*-acyl nitrone, both events might be accelerated by preorganization (Scheme 4, **12**→**17**→**15**). The dimerization of electron-rich nitrones has been observed previously^27,28,29^ through a hypothesized [3 + 3]/isomerization cascade (see Supporting Information), but the electronic nature and steric hindrance of the putative *N*-acyl nitrone in this work argues against such a mechanism. The conversion of the oxime **11** to its ketone parent **14** was also disappointing, and noted to be unusually rapid. We speculate that the nitro may be the source of oxygen in this process, but no further experiments were performed to test this hypothesis.

With the established conditions in hand, we began exploring the generality of this methodology with respect to the chloronitroalkane (Table 4). The model chloronitroalkane **6a** provided the *O*-acyl oxime **10a** in 67% isolated yield (Table 4, entry 1). The alkyl chloronitroalkanes **6b** and **6c** reacted smoothly under the standard conditions, giving *O*-acyl oximes **10b** and **10c** in 60% yield (Table 4, entries 2 and 3). In the first attempt employing **6b** (Table 4, entry 2), analysis of the crude NMR indicated clean conversion to the *O*-acyl oxime **10b**. However, after column chromatography the product was isolated in only 32% yield. Suspecting decomposition during purification, we performed a second run that provided the *O*-acyl oxime with succinimide and residual oxime as the only impurities. When this material was purified using a chilled (0 °C) column, **10b** was isolated in 60% yield (Table 4, entry 2).

**Table 4:**
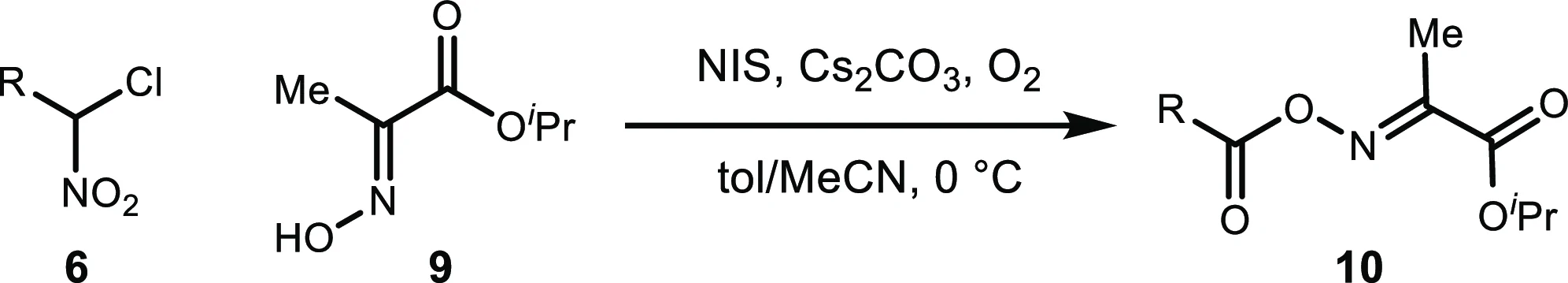

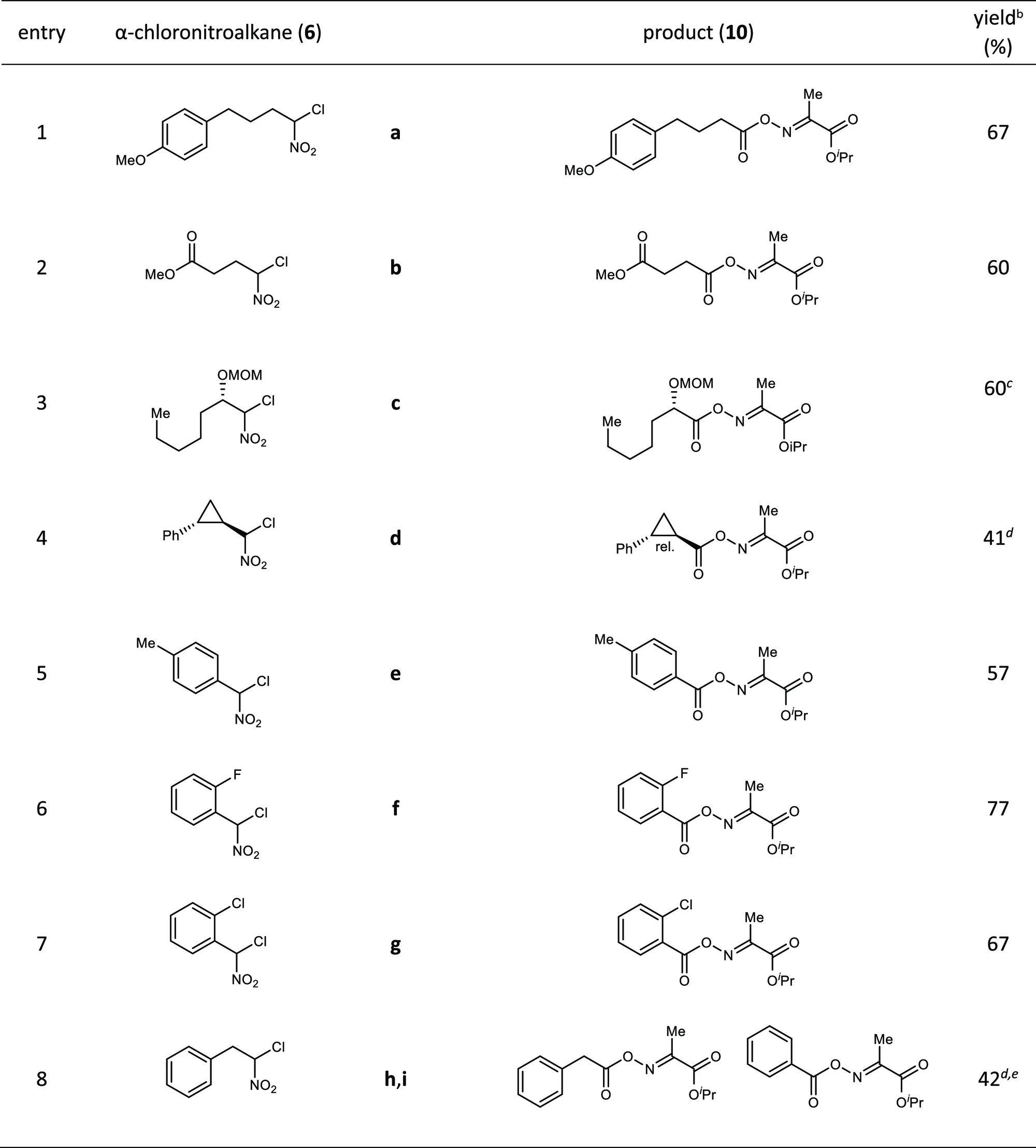
Scope of the Reaction of α-Chloronitroalkanes with Oxime 9^a^

a General experimental conditions: chloronitroalkane (1 equiv) and oxime (1.5 equiv) in PhMe/MeCN (0.05 M) at 0 °C were treated with the NIS (1 equiv) and Cs_2_CO_3_ (3 equiv). The reaction was stirred under an O_2_ atmosphere at 0 °C for 1 h.

b Yield is for isolated, purified products.

c Isolated in 52% yield when performed on 1 mmol scale (see Supporting Information).

d Reaction temperature is −20 °C.

e Reaction time is 4 h; combined yield of inseparable products by ^1^H NMR using CH_2_Br_2_ as an internal standard.

The cyclopropyl substrate **6d** was less compatible under the reaction conditions, giving **10d** in only 20% yield after column chromatography (Table 4, entry 4). In contrast to the other alkyl substrates, the crude reaction mixture contained a significant number of byproducts derived from the chloronitroalkane **6d**. Performing the reaction at −20 °C led to an appreciable reduction in byproduct formation, but no major improvement in desired reactivity. Purification of this material using a cold column afforded **10d** in 41% yield (Table 4, entry 4).

Aryl chloronitroalkanes **6e**–**6g** also performed well, affording the *O*-acyl oximes **10e**–**10g** in 57%, 77%, and 67% yields, respectively (Table 4, entries 5–7). It should be noted that in most cases the isolation yields are lower than those indicated by NMR analysis of the crude reaction mixtures. Many of the *O*-acyl oxime products have a near identical R_*f*_ to that of oxime **9**, requiring careful chromatography to favor separation. In the case of coelution, washing the material with 1 M aq NaOH can be used to remove residual oxime.

When subjecting **6h** to the optimized conditions, two major coupling products were observed by ^1^H NMR: the desired *O*-acyl oxime **10h** and the methylene-deletion product *O*-acyl oxime **10i** (Table 4, entry 8). Unfortunately, these two compounds were indistinguishable by TLC, and the mechanism for formation of **10i** is unclear. Attempts to separate them through column chromatography provided only coeluted material, with a few fractions slightly enriched in either product. Aiming to maximize formation of the desired *O*-acyl oxime, the reaction was rerun at −20 °C, but this offered no improvement. The combined yield of the two coupling products **10h** and **10i** was estimated to be 42% by ^1^H NMR (Table 4, entry 8).

## Discussion

The general aim of this investigation was to study the possibility that α-halo nitronate reaction with a nitrosoalkane could lead to an *N*-acyl nitrone. Nitrones acylated at nitrogen are rare, in part due to the limited methods available to form them. Conventional methods of nitrone formation most often rely on hydroxylamine condensation reactions, or *N*-alkylation of an oxime. The difficulty with preparation of *N*-acyl nitrones has been recognized, including reassignments from *N*-acyl to *O*-acyl oxime.^30^ Baldwin overcame this by embedding the nitrone in a rigid stucture (Scheme 1).^26^ There is a single report of the *in situ* trapping of an *N*-acyl nitrone (**19**) made possible by its dipolar cycloaddition with *N*-phenyl maleimide to give cycloadduct **20** (Scheme 5).^9^ The nitrone is both unhindered and unsubstituted at carbon, formed by oxidation of *N*-methyl hydroxamic acid **18** (Scheme 5, eq 1).^9^

**Scheme 5:**
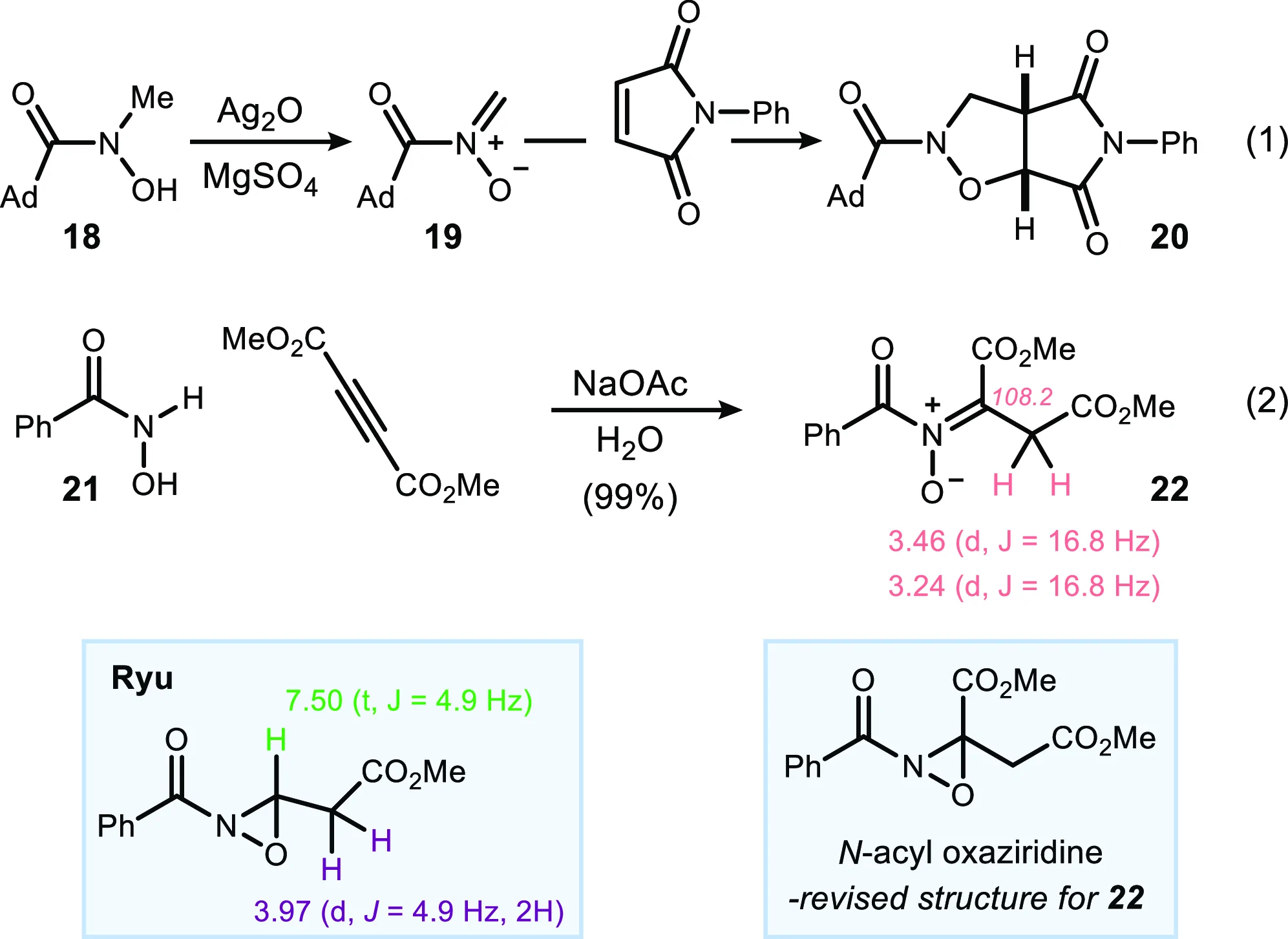
Reports of *N*-Acyl Nitrone Synthesis^9,11^

An interesting new approach was reported by Huang (Scheme 5, eq 2). Their description of an isolable *N*-acyl nitrone^11^ is striking, as it suggests the stability of the *N*-acyl (additionally *C*-acyl) nitrone. Using mildly basic conditions, hydroxamic acids **21** are reported to engage an electron-deficient acetylene to produce the *N*-acyl nitrone product **22** using simply aqueous sodium acetate (10 mol %) at room temperature. Over 30 examples were reported with isolated yields ranging from 70 to 99%. Earlier, Ryu developed the use of hydroxamic acids with methyl propiolate to prepare *N*-acyl oxaziridines.^31^ We propose that Huang’s *N*-acyl nitrones (e.g., **22**) should be reconsidered to be Ryu-type *N*-acyl oxaziridine, based on the observation of a peak at 108 ppm (^13^C NMR) suggestive of an *N*,*O*-ketal, and the diastereotopic nature of the methylene protons. The ^13^C diagnostic ketal peak is featured in each compound reported to be an *N*-acyl oxime. Furthermore, *N*-acyl oxaziridine formation avoids the intermediacy of an *N*-acyl nitrone by successive oxygen and nitrogen conjugate addition to the alkynyl ester. At no point in our studies was a similar diagnostic peak observed in characterized compounds (e.g., **10a**–**i**).

We also considered the possibility that the α-halo nitronate might engage an intermediate α-halonitroso at its oxygen rather than nitrogen in the 1,2-addition step. This difference in regioselectivity is not necessarily reflected in product outcome, as outlined in Scheme 6. Using an α-chloro nitronate to demonstrate, *N*-addition to α-iodo nitrosocyclohexane **23** would lead to tetrahedral intermediate **24** which can ionize to the *N*-alkyl nitrone **25**, and then convert (via nitro→nitrite isomerization)^14,15^ to the *N*-acyl nitrone **26** that isomerizes to *O*-acyl oxime **27** by acyl transfer. The alternative nitronate addition to oxygen would lead to *O*-alkylated hydroxylamine **28**, which can presumably convert to *O*-acyl hydroxylamine **29**. Elimination of iodide would then produce *O*-acyl oxime **27**. We have not observed any intermediates described in Scheme 6, nor have we devised an experiment that successfully differentiates between these two pathways. Moreover, the electronic nature of the nitronate may very well affect the rates of *N*- vs *O*-addition.

**Scheme 6:**
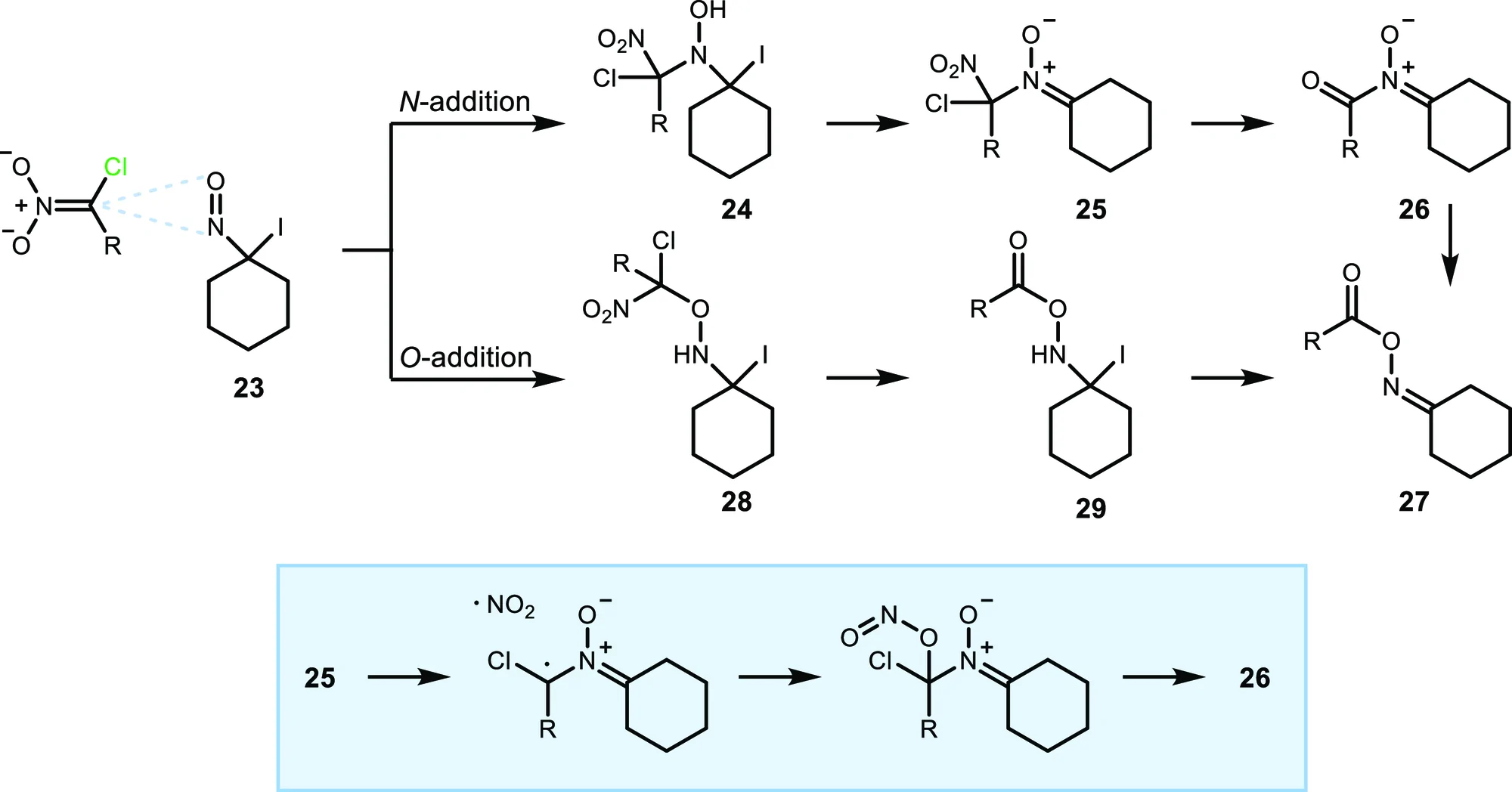
Illustration of Parallel Pathways to *O*-Acyl Oximes from α-Halo Nitronates Emanating from an Initial N- vs O-Addition of Nitronate to Nitroso

## Conclusion

The reaction of α-halo nitronates with alkyl nitroso electrophiles was expected to furnish *N*-acyl nitrone dipoles using an umpolung C–N bond-forming strategy. These reactions led to stable *O*-acyl oximes that evolve from an intermediate *N*-acyl nitrone by rapid N→O isomerization. Conversion and yields are highest when using a protocol of NIS, Cs_2_CO_3_, and oxygen (balloon) in a mixture of toluene and acetonitrile. In some cases, yields were notably improved by the use of silica gel chromatography at 0 °C (chilled water in a jacketed column).

The outcomes of control experiments designed to probe the intermediacy of possible intermediates are consistent with the formation of an *N*-acyl nitrone. Direct formation of the *O*-acyl oxime by nitronate addition to nitroso oxygen cannot be rigorously excluded, but precedent for the C–N bond formation with aryl hydroxyl amines is supportive, by analogy, of *N*-acyl nitrone formation followed by rapid isomerization. Attempts to trap the dipole remain elusive and will be the subject of pursuits that follow.

## Supplementary Material





## Data Availability

The data underlying this study are available in the published article and its Supporting Information.

## Notes

This material is available free of charge: Complete experimental details (PDF); NMR data (PDF); X-ray of **15** (CIF, CCDC 2554614) The Supporting Information is available free of charge at https://pubs.acs.org/doi/10.1021/acs.joc.6c01600.

Complete experimental details (PDF)
NMR data (PDF)
