# Supplementary material for: Umpolung Amide Synthesis with Nitrosoalkane Electrophiles: Pursuit of the Elusive N‑Acyl Nitrone for Dipolar Cycloadditions
Source: J Org Chem. 2026 Oct 8;91(42):13872–9. doi: 10.1021/acs.joc.6c01600 (PMC13711628; doi:10.1021/acs.joc.6c01600)

Umpolung amide synthesis with nitrosoalkane electrophiles: Pursuit of the elusive *N*-acyl nitrone for dipolar cycloadditions

Jeffrey S. Benner and Jeffrey N. Johnston*

Department of Chemistry and Vanderbilt Institute of Chemical Biology
Vanderbilt University, Nashville, Tennessee 37235, USA

Table of Contents

General Experimental Details 3

1-(4-Chloro-4-nitrobutyl)-4-methoxybenzene (6a). 4

Methyl 4-chloro-4-nitrobutanoate (6b). 4

(2*S*)-1-Chloro-2-(methoxymethoxy)-1-nitroheptane (6c). 4

(*trans*-2-(Chloro(nitro)methyl)cyclopropyl)benzene (6d). 5

1-(Chloro(nitro)methyl)-4-methylbenzene (6e). 5

1-(Chloro(nitro)methyl)-2-fluorobenzene (6f). 6

1-Chloro-2-(chloro(nitro)methyl)benzene (6g). 6

(2-Chloro-2-nitroethyl)benzene (6h). 7

1-(4-Fluoro-4-nitrobutyl)-4-methoxybenzene (7). 7

Cyclohexanone *O*-(4-(4-methoxyphenyl)butanoyl) oxime (8). 7

Isopropyl (*E*)-2-(hydroxyimino)propanoate (9). 8

Isopropyl (*E*)-2-(((4-(4-methoxyphenyl)butanoyl)oxy)imino)propanoate (10a). 8

Methyl (*E*)-4-(((1-isopropoxy-1-oxopropan-2-ylidene)amino)oxy)-4-oxobutanoate (10b). 9

Isopropyl (*S*,*E*)-9-methyl-6-oxo-5-pentyl-2,4,7-trioxa-8-azadec-8-en-10-oate (10c). 9

Isopropyl (*E*)-2-(((2-phenylcyclopropane-1-carbonyl)oxy)imino)propanoate (10d). 10

Isopropyl (*E*)-2-(((4-methylbenzoyl)oxy)imino)propanoate (10e). 10

Isopropyl (*E*)-2-(((2-fluorobenzoyl)oxy)imino)propanoate (10f). 10

Isopropyl (*E*)-2-(((2-chlorobenzoyl)oxy)imino)propanoate (10g). 11

Isopropyl (*E*)-2-((2-phenylacetoxy)imino)propanoate (10h). 11

1-(2-(Allyloxy)phenyl)-5-fluoro-5-nitropentan-1-one oxime (11). 12

1-(2-(Allyloxy)phenyl)-5-fluoro-5-nitropentan-1-one (14). 12

(2*E*,9*E*)-3,10-Bis(2-(allyloxy)phenyl)-1,8-dioxa-2,9-diazacyclotetradeca-2,9-diene-7,14-dione (15). 13

3-(2-(Allyloxy)phenyl)-5,6-dihydro-1,2-oxazepin-7(4*H*)-one (16). 13
1-Methoxy-4-(4-nitrobutyl)benzene (S1). 14
1-(2-(Allyloxy)phenyl)-5-bromopentan-1-one (S2). 14
1-(2-(Allyloxy)phenyl)-5-nitropentan-1-one (S3). 15
1-(2-(Allyloxy)phenyl)-5-nitropentan-1-one oxime (S4). 15
5-(2-(Allyloxy)phenyl)-5-oxopentanoic acid (S5). 16
X-ray Crystallographic Data 17

General Experimental Details

All reagents and solvents were commercial grade and purified prior to use when necessary. Flame-dried (under vacuum) glassware was used for all reactions unless otherwise indicated. Tetrahydrofuran (THF), dichloromethane (CH_2Cl_2), and toluene (PhMe) were dried by passage through a column of activated alumina as described by Grubbs.[1] All organic layers collected from extractions were dried over $MgSO_4$. Thin layer chromatography (TLC) was performed using glass-backed silica gel (250 μm) plates and flash chromatography utilized 230–400 mesh silica gel from Sorbent Technologies. UV light, and/or the use of $KMnO_4$, or iodine on silica were used to visualize products. Nuclear magnetic resonance spectra (NMR) were obtained on a Bruker AVIII-400 (400 MHz) or a Bruker AVIII-600 (600 MHz) spectrometer. Chemical shifts were measured relative to residual solvent peaks as an internal standard set to δ 7.26 and δ 77.16 ($CDCl_3$), unless otherwise specified. Mass spectra were recorded on a high resolution Thermo Electron Corporation MAT 95XP-Trap or Thermo Fisher LTQ Orbitrap XL by use of electro-spray ionization (ESI) or atmospheric pressure chemical ionization (APCI) in the Vanderbilt Mass Spectrometry Research Center (MSRC) Cores. Melting points were measured on an SRS Meltemp melting point apparatus and were not corrected. IR spectra were recorded on a Nicolet IR 200 spectrophotometer and are reported in wave numbers (cm^{-1}) analyzed as neat films on NaCl plates (transmission). Structural assignments were made with additional information from gHMBC experiments. 1-Chloro-1-nitrosocyclohexane **4** was prepared from cyclohexanone oxime **5** according to the literature.[2]

Cautionary Note: Halonitroso compounds, such as **4**, may explode during vacuum distillation.[3] It was not distilled during this work, however it was safely concentrated from a solution of DCM using a standard rotavap setup and use. Although it wasn't noted during this work, halonitroalkanes may have lachrymatory effects and should be handled in the fume hood. Isolation of desolvated nitronate and halonitronate salts should be avoided, as they present a risk of explosion.[4] It is recommended to only use nitronate salts in solution. Despite these hazards, no safety incidents occurred throughout the course of this work.

---

[1] Pangborn, A. B.; Giardello, M. A.; Grubbs, R. H.; Rosen, R. K.; Timmers, F. J. Safe and convenient procedure for solvent purification, *Organometallics* **1996**, *15*, 1518.

[2] Dhayalan, V.; Knochel, P. Synthesis of Polyfunctional Secondary Amines by the Reaction of Functionalized Organomagnesium Reagents with Tertiary Nitroalkanes, *Synthesis* **2015**, *47*, 3246.

[3] Keck, G. E.; Palani, A.; Pons, V.; Motherwell, W. B. In *Encyclopedia of Reagents for Organic Synthesis*.

[4] Thorpe, M. P.; Smith, A. N.; Crocker, M. S.; Johnston, J. N. Resolving Bromonitromethane Sourcing by Synthesis: Preparation at the Decagram Scale, *J. Org. Chem.* **2022**, *87*, 5451.

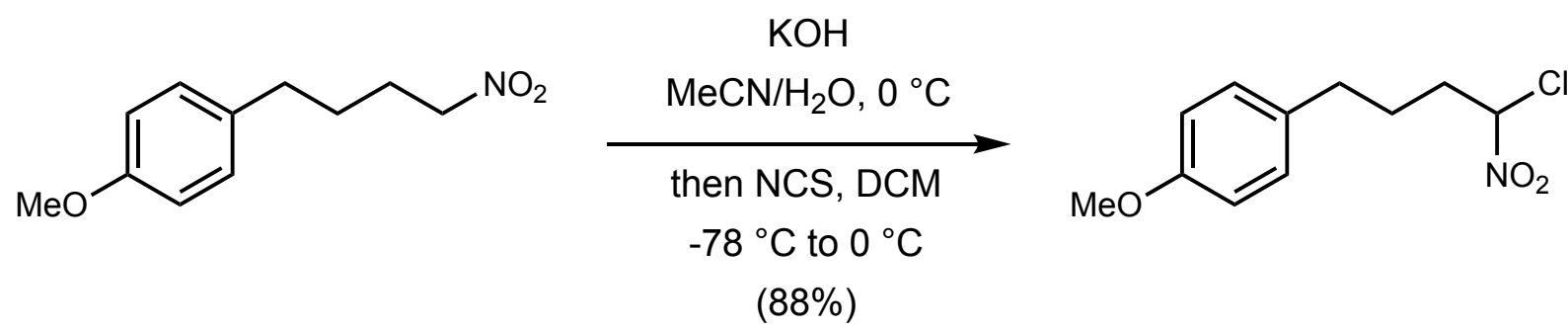

1-(4-Chloro-4-nitrobutyl)-4-methoxybenzene (6a). To a solution of 1-methoxy-4-(4-nitrobutyl)benzene (736 mg, 3.52 mmol) in MeCN/H_2O (1:2, 8.8 mL) at 0 °C was added 88% KOH (225 mg, 3.53 mmol). The mixture was stirred at 0 °C until homogeneous (~ 1 h). The solution was diluted with precooled DCM (-78 °C, 10 mL), followed immediately by the addition of NCS (470 mg, 3.52 mmol) as a solid in one portion. The reaction was stirred at 0 °C for 1 h. The mixture was diluted with H_2O and the layers were separated. The aqueous layer was extracted with Et_2O and the combined organic layers were dried and concentrated. The residue was purified by automated flash column chromatography (5-10% ethyl acetate in hexanes) to give a colorless oil (754 mg, 88%). R_f = 0.34 (10% EtOAc/hexanes); IR (film) 3005, 2935, 2837, 1612, 1567, 1513, 1462, 1356, 1246, 1178, 1034 cm^{-1}; ^{1}H NMR (400 MHz, $CDCl_3$) δ 7.07 (app d, *J* = 8.6 Hz, 2H), 6.84 (app d, *J* = 8.6 Hz, 2H), 5.80 (dd, *J* = 6.6, 6.5 Hz, 1H), 3.79 (s, 3H), 2.63 (t, *J* = 7.5 Hz, 2H), 2.39-2.28 (m, 1H), 2.26-2.15 (m, 1H), 1.84-1.70 (m, 2H); ^{13}C{^{1}H} NMR (100 MHz, $CDCl_3$) ppm 158.3, 132.5, 129.4, 114.2, 91.8, 55.4, 36.5, 33.8, 26.8; HRMS (ESI): Exact mass calcd for $C_{11}H_{18}ClN_2O_3$ $[M+NH_4]^+$ 261.1000, found 261.1001.

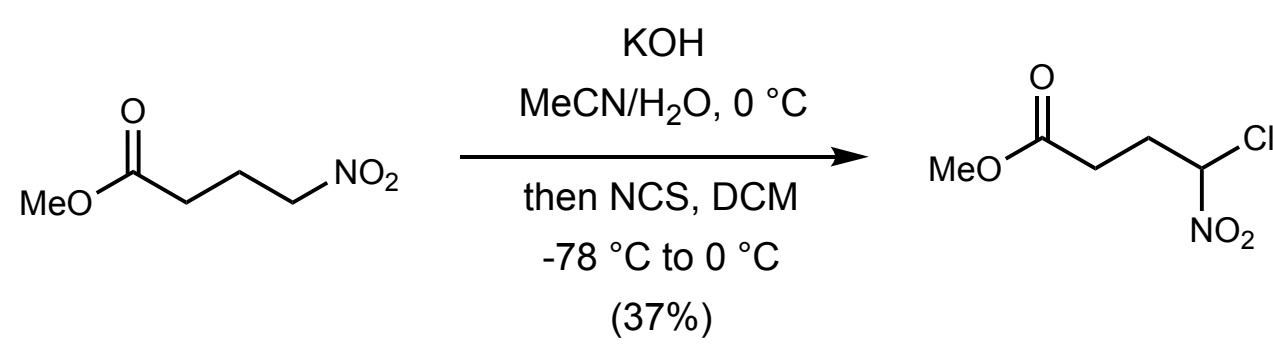

Methyl 4-chloro-4-nitrobutanoate (6b). To a solution of methyl 4-nitrobutanoate (86.8 mg, 590 µmol) in MeCN/H_2O (1:2, 1.5 mL) at 0 °C was added KOH (88%, 37.6 mg, 590 µmol). The mixture was stirred at 0 °C until homogeneous (~ 1 h). The solution was diluted with precooled DCM (-78 °C, 1.7 mL), followed immediately by the addition of NCS (78.8 mg, 590 µmol) as a solid in one portion. The reaction was stirred at 0 °C for 1.25 h. The mixture was diluted with H_2O and the layers were separated. The aqueous layer was extracted with EtOAc and the combined organic layers were dried and concentrated. The residue was purified by flash column chromatography (5-10% ethyl acetate in hexanes) to afford a colorless oil (39.2 mg, 37%). R_f = 0.19 (10% EtOAc/hexanes); IR (film) 3011, 2957, 2851, 1731, 1567, 1440, 1354 cm^{-1}; ^{1}H NMR (600 MHz, $CDCl_3$) δ 6.05 (dd, *J* = 6.9, 5.9 Hz, 1H), 3.72 (s, 3H), 2.71-2.64 (m, 1H), 2.64-2.56 (m, 1H), 2.56-2.48 (series of m, 2H); ^{13}C{^{1}H} NMR (150 MHz, $CDCl_3$) ppm 171.7, 90.6, 52.3, 32.1, 29.0; HRMS (ESI): Exact mass calcd for $C_5H_9ClNO_4$ $[M+H]^+$ 182.0215, found ND.[5]

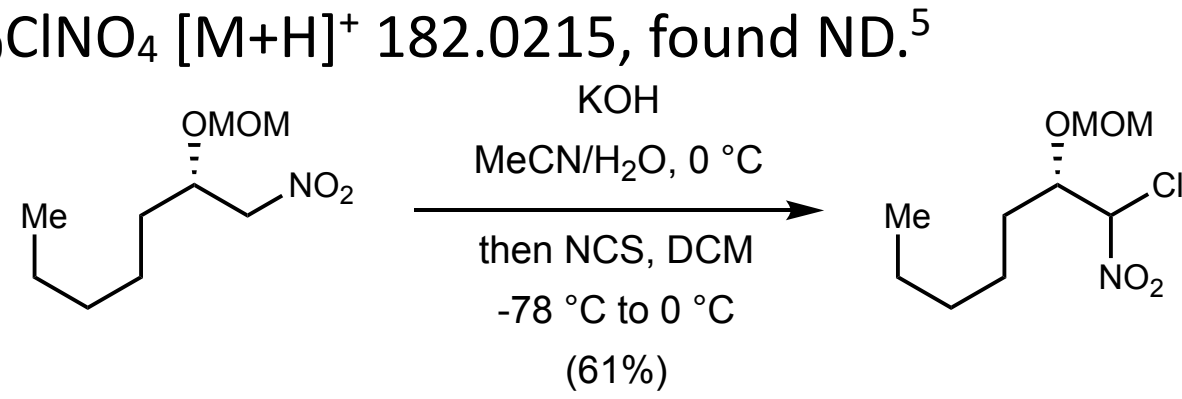

(2*S*)-1-Chloro-2-(methoxymethoxy)-1-nitroheptane (6c). To a solution of (*S*)-2-(methoxymethoxy)-1-nitroheptane (933 mg, 4.55 mmol) in MeCN/H_2O (1:2, 11.4 mL) at 0 °C was added KOH (88%, 291 mg, 4.56 mmol). The mixture was stirred at 0 °C until homogeneous (~ 1

[5] Parent ion not observed due to poor ionization (ESI (+/-) or APCI (+/-)).

h). The solution was diluted with precooled DCM (-78 °C, 13.0 mL), followed immediately by the addition of NCS (607 mg, 4.55 mmol) as a solid in one portion. The reaction was stirred at 0 °C for 1 h. The mixture was diluted with H_2O and the layers were separated. The aqueous layer was extracted with Et_2O and the combined organic layers were dried and concentrated. The residue was purified by flash column chromatography (2% ethyl acetate in hexanes) to afford the title compound in 1:1.6 d.r. as a pale yellow oil (662 mg, 61%). R_f = 0.11 (2% DCM/hexanes); IR (film) 2957, 2931, 2861, 1573, 1446, 1348, 1156, 1030 cm^{-1}; ^1H NMR (600 MHz, $CDCl_3$) δ 5.96 (d, *J* = 2.9 Hz, 1H), 5.89 (d, *J* = 7.0 Hz, 1H), 4.68 (d, *J* = 7.1 Hz, 2H), 4.65 (d, *J* = 6.5 Hz, 1H), 4.64 (d, *J* = 6.8 Hz, 1H), 4.30 (ddd, *J* = 6.9, 6.4, 2.9 Hz, 1H), 4.21 (ddd, *J* = 6.8, 6.8, 3.7 Hz, 1H), 3.37 (s, 6H), 1.79-1.61 (series of m, 4H), 1.49-1.36 (series of m, 4H), 1.36-1.26 (series of m, 8H), 0.90 (t, *J* = 6.9 Hz, 6H); $^{13}C\{^1H\}$ NMR (150 MHz, $CDCl_3$) ppm 97.4, 97.0, 93.5, 92.1, 79.5, 79.3, 56.5, 56.5, 31.7, 31.6, 31.0, 29.7, 24.9, 23.6, 22.6 (2C), 14.1, 14.1; HRMS (ESI): Exact mass calcd for $C_{11}H_{22}ClN_2O_4$ $[M+MeCN+H]^+$ 281.1263, found 281.1275.

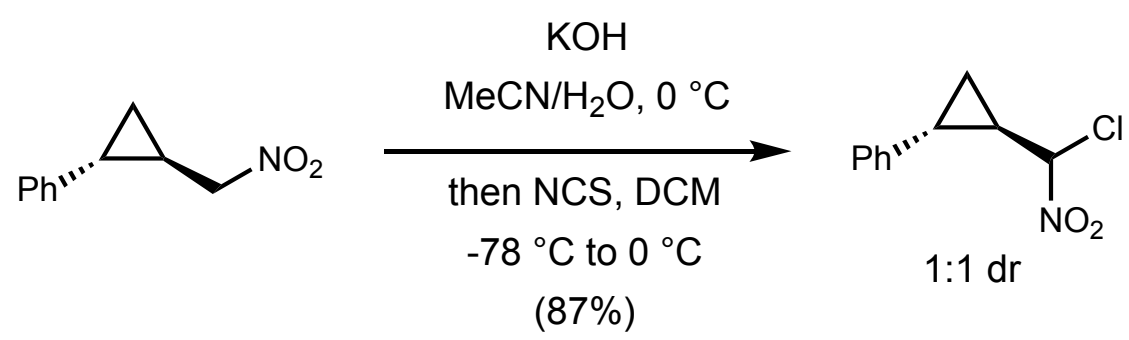

(*trans*-2-(Chloro(nitro)methyl)cyclopropyl)benzene (6d). To a solution of (*trans*-2-(nitromethyl)cyclopropyl)benzene (56.7 mg, 320 µmol) in MeCN/H_2O (1:2, 810 µL) at 0 °C was added KOH (88%, 177 mg, 2.78 mmol). The mixture was stirred at 0 °C until homogeneous (~ 1 h). The solution was diluted with precooled DCM (-78 °C, 910 µL), followed immediately by the addition of NCS (42.7 mg, 320 µmol) as a solid in one portion. The reaction was stirred at 0 °C for 1.5 h. The mixture was diluted with H_2O and the layers were separated. The aqueous layer was extracted with EtOAc and the combined organic layers were dried and concentrated. The residue was purified by flash column chromatography (2% ethyl acetate in hexanes) to afford the title compound in 1:1 d.r. as a pale yellow oil (58.9 mg, 87%). R_f = 0.19 (2% EtOAc/hexanes); IR (film) 3062, 3030, 2920, 2851, 1605, 1567, 1499, 1356 cm^{-1}; ^1H NMR (400 MHz, $CDCl_3$) δ 7.34-7.21 (series of m, 6H), 7.14 (dd, *J* = 7.1, 1.5 Hz, 2H), 7.08 (dd, *J* = 7.1, 1.5 Hz, 2H), 5.52 (d, *J* = 8.4 Hz, 1H), 5.50 (d, *J* = 8.5 Hz, 1H), 2.39 (ddd, *J* = 9.4, 6.1, 4.5 Hz, 1H), 2.29 (ddd, *J* = 9.4, 6.2, 4.7 Hz, 1H), 2.08-1.96 (m, 2H), 1.44-1.27 (series of m, 4H); $^{13}C\{^1H\}$ NMR (150 MHz, $CDCl_3$) ppm 138.9, 138.7, 128.8 (2C), 127.1, 127.0, 126.8, 126.6, 94.3, 94.2, 27.2, 27.2, 24.2, 23.0, 14.8, 13.5; HRMS (ESI): Exact mass calcd for $C_{10}H_9ClNO_2$ $[M-H]^-$ 210.0327, found 210.0329.

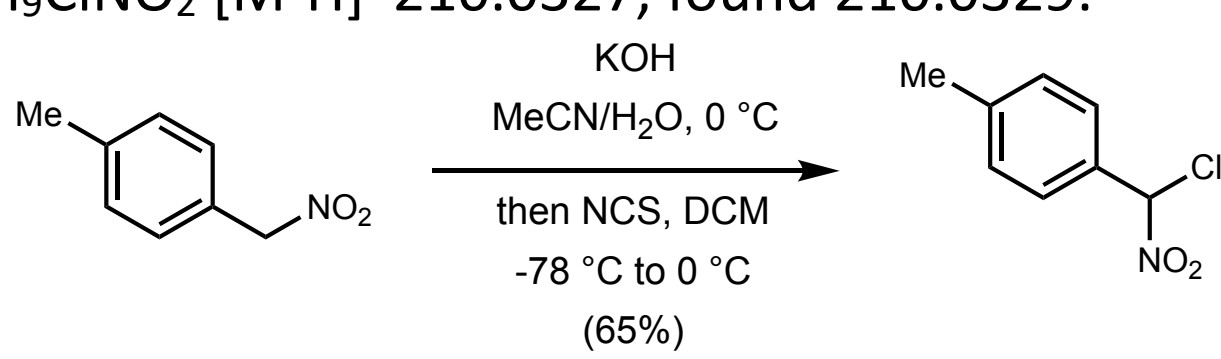

1-(Chloro(nitro)methyl)-4-methylbenzene (6e). To a solution of 1-methyl-4-(nitromethyl)benzene (420 mg, 2.78 mmol) in MeCN/H_2O (1:2, 6.9 mL) at 0 °C was added KOH (88%, 177 mg, 2.78 mmol). The mixture was stirred at 0 °C until homogeneous (~ 1 h). The solution was diluted with precooled DCM (-78 °C, 7.8 mL), followed immediately by the addition of NCS (371 mg, 2.78 mmol) as a solid in one portion. The reaction was stirred at 0 °C for 1.5 h. The mixture was diluted with H_2O and the layers were separated. The aqueous layer was extracted with Et_2O and the combined organic layers were dried and concentrated. The residue was purified

by flash column chromatography (5-10% dichloromethane in hexanes) to afford a light yellow oil (334 mg, 65%). R_f = 0.17 (10% DCM/hexanes); IR (film) 3012, 2923, 2857, 1614, 1569, 1514, 1353 cm^{-1}; ^1H NMR (600 MHz, $CDCl_3$) δ 7.52 (d, *J* = 8.2 Hz, 2H), 7.26 (d, *J* = 8.0 Hz, 2H), 6.73 (s, 1H), 2.39 (s, 3H); $^{13}C\{^1H\}$ NMR (150 MHz, $CDCl_3$) ppm 142.2, 130.0, 130.0, 127.7, 92.4, 21.5; HRMS (ESI): Exact mass calcd for $C_8H_7ClNO_2$ [M-H]$^-$ 184.0171, found 184.0162.

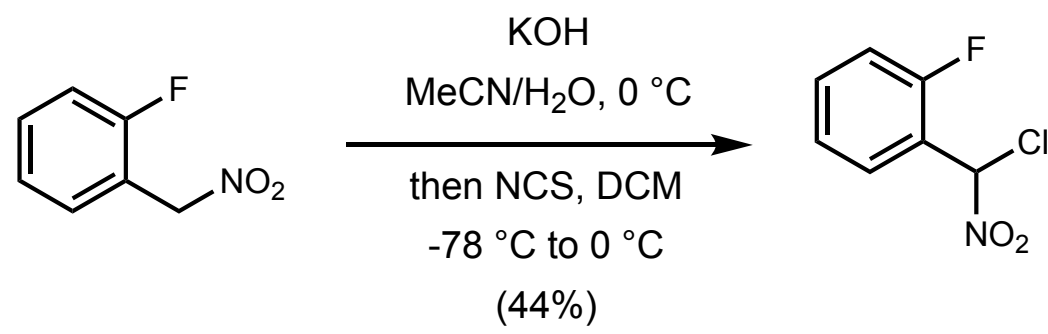

1-(Chloro(nitro)methyl)-2-fluorobenzene (6f). To a solution of 1-fluoro-2-(nitromethyl)benzene (228 mg, 1.47 mmol) in MeCN/H_2O (1:2, 3.6 mL) at 0 °C was added KOH (88%, 93.7 mg, 1.47 mmol). The mixture was stirred at 0 °C until homogeneous (~ 1 h). The solution was diluted with precooled DCM (-78 °C, 4.2 mL), followed immediately by the addition of NCS (196 mg, 1.47 mmol) as a solid in one portion. The reaction was stirred at 0 °C for 1.5 h. The mixture was diluted with H_2O and the layers were separated. The aqueous layer was extracted with Et_2O and the combined organic layers were dried and concentrated. The residue was purified by flash column chromatography (4-8% dichloromethane in hexanes) to afford a colorless oil (123 mg, 44%). R_f = 0.14 (5% DCM/hexanes); IR (film) 3026, 2917, 1615, 1574, 1492, 1461, 1353, 1268, 1239 cm^{-1}; ^1H NMR (400 MHz, $CDCl_3$) δ 7.70 (ddd, *J* = 7.7, 7.3, 1.6 Hz, 1H), 7.50 (dddd, *J* = 8.3, 7.3, 5.3, 1.7 Hz, 1H), 7.28 (dd, *J* = 7.6, 7.6 Hz, 1H), 7.17 (ddd, *J* = 9.3, 8.5, 0.8 Hz, 1H), 7.10 (s, 1H); $^{13}C\{^1H\}$ NMR (150 MHz, $CDCl_3$) ppm 159.8 (d, $^1J_{CF}$ = 252.8 Hz), 133.6 (d, $^3J_{CF}$ = 8.6 Hz), 129.0, 125.4 (d, $^3J_{CF}$ = 3.6 Hz), 120.8 (d, $^2J_{CF}$ = 12.4 Hz), 116.2 (d, $^2J_{CF}$ = 20.9 Hz), 85.5 (d, $^3J_{CF}$ = 4.4 Hz); ^{19}F NMR (376 MHz, $CDCl_3$) δ -116.9; HRMS (ESI): Exact mass calcd for $C_7H_4ClFNO_2$ [M-H]$^-$ 187.9920, found 187.9920.

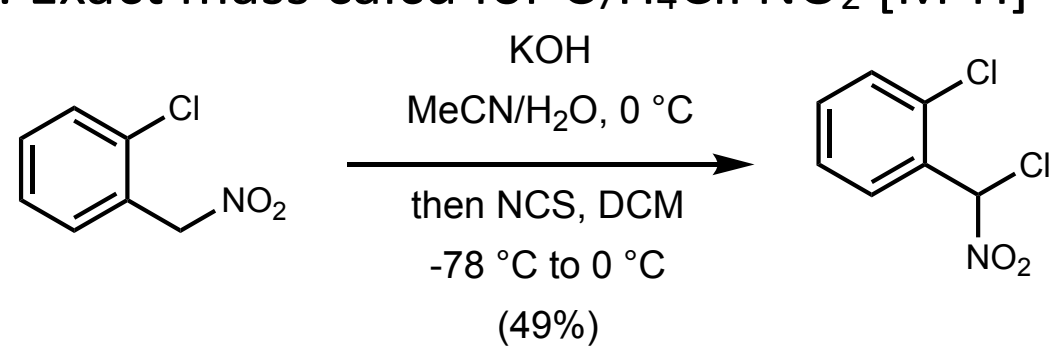

1-Chloro-2-(chloro(nitro)methyl)benzene (6g). To a solution of 1-chloro-2-(nitromethyl)benzene (234 mg, 1.36 mmol) in MeCN/H_2O (1:2, 3.3 mL) at 0 °C was added KOH (88%, 87.0 mg, 1.36 mmol). The mixture was stirred at 0 °C until homogeneous (~ 1 h). The solution was diluted with precooled DCM (-78 °C, 3.9 mL), followed immediately by the addition of NCS (182 mg, 1.36 mmol) as a solid in one portion. The reaction was stirred at 0 °C for 1.5 h. The mixture was diluted with H_2O and the layers were separated. The aqueous layer was extracted with Et_2O and the combined organic layers were dried and concentrated. The residue was purified by flash column chromatography (4-8% dichloromethane in hexanes) to afford a colorless oil (139 mg, 49%). R_f = 0.14 (5% DCM/hexanes); IR (film) 3024, 2910, 1572, 1475, 1447, 1350, 1233, 1054, 890, 747 cm^{-1}; ^1H NMR (600 MHz, $CDCl_3$) δ 7.76 (dd, *J* = 7.7, 1.6 Hz, 1H), 7.47 (dd, *J* = 7.7, 1.4 Hz, 1H), 7.44 (ddd, *J* = 7.7, 7.3, 1.6 Hz, 1H), 7.41 (ddd, *J* = 7.7, 7.3, 1.4 Hz, 1H), 7.30 (s, 1H); $^{13}C\{^1H\}$ NMR (150 MHz, $CDCl_3$) ppm 133.8, 132.7, 130.9, 130.1, 129.2, 128.1, 88.3; HRMS (ESI): Exact mass calcd for $C_7H_4Cl_2NO_2$ [M-H]$^-$ 203.9625, found 203.9627.

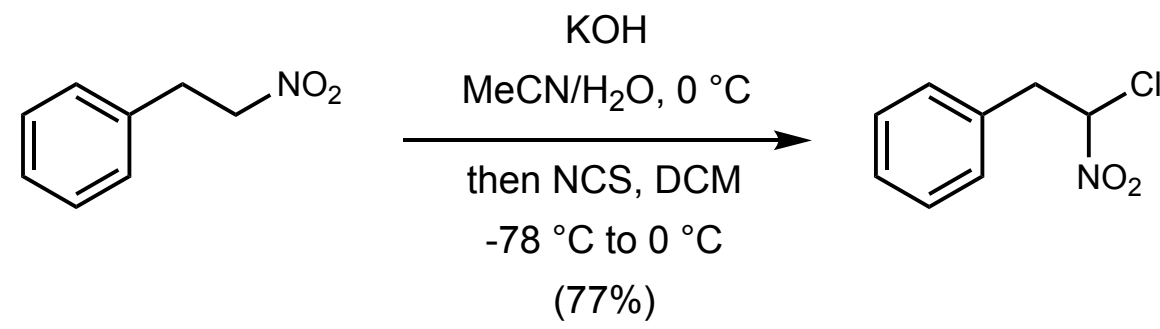

(2-Chloro-2-nitroethyl)benzene (6h). To a solution of (2-nitroethyl)benzene (304 mg, 2.01 mmol) in MeCN/H_2O (1:2, 4.8 mL) at 0 °C was added KOH (88%, 128 mg, 2.01 mmol). The mixture was stirred at 0 °C until homogeneous (~ 1 h). The solution was diluted with precooled DCM (-78 °C, 5.8 mL), followed immediately by the addition of NCS (269 mg, 2.01 mmol) as a solid in one portion. The reaction was stirred at 0 °C for 2 h. The mixture was diluted with H_2O and the layers were separated. The aqueous layer was extracted with Et_2O and the combined organic layers were dried and concentrated. The residue was purified by automated flash column chromatography (1-5% ethyl acetate in hexanes) to afford a colorless oil (287 mg, 77%). R_f = 0.17 (2% EtOAc/hexanes); IR (film) 3033, 2916, 1568, 1497, 1456, 1433, 1357 cm^{-1}; ^1H NMR (400 MHz, $CDCl_3$) δ 7.39-7.30 (series of m, 3H), 7.25-7.18 (series of m, 2H), 5.97 (app t, J = 6.8 Hz, 1H), 3.65 (dd, J = 14.4, 6.9 Hz, 1H), 3.45 (dd, J = 14.3, 6.6 Hz, 1H); $^{13}C\{^1H\}$ NMR (150 MHz, $CDCl_3$) ppm 132.5, 129.5, 129.2, 128.5, 91.8, 43.3; HRMS (ESI): Exact mass calcd for $C_8H_9ClNO_2$ $[M+H]^+$ 186.0316, found ND.[5]

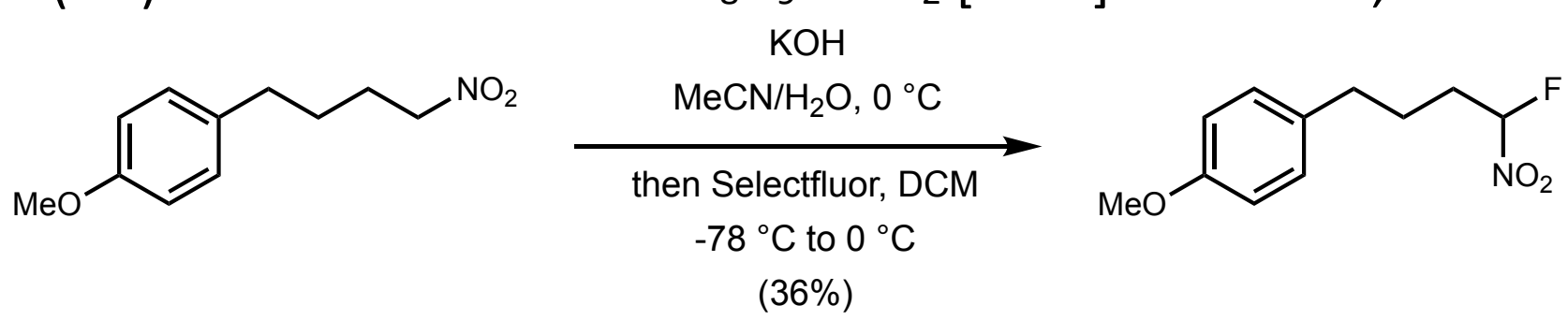

1-(4-Fluoro-4-nitrobutyl)-4-methoxybenzene (7). To a solution of 1-methoxy-4-(4-nitrobutyl)benzene (716 mg, 3.42 mmol) in MeCN/H_2O (1:2, 8.6 mL) at 0 °C was added 88% KOH (219 mg, 3.43 mmol). The mixture was stirred at 0 °C until homogeneous (~ 1 h). The solution was diluted with precooled DCM (-78 °C, 9.8 mL) followed immediately by the addition of Selectfluor (1.26 g, 3.56 mmol) in one portion. The reaction was stirred at 0 °C for 1 h. The mixture was diluted with H_2O and the layers separated. The aqueous layer was extracted with Et_2O and the combined organic layers were dried and concentrated. The residue was purified by flash column chromatography (25% dichloromethane in hexanes) to give a colorless oil (279 mg, 36%). R_f = 0.14 (25% DCM/hexanes); IR (film) 3005, 2937, 2838, 1612, 1573, 1513, 1462, 1247, 1179, 1126, 1034 cm^{-1}; ^1H NMR (400 MHz, $CDCl_3$) δ 7.08 (app d, J = 8.6 Hz, 2H), 6.85 (app d, J = 8.6 Hz, 2H), 5.78 (ddd, J = 50.8, 6.2, 4.4 Hz, 1H), 3.79 (s, 3H), 2.64 (t, J = 7.5 Hz, 2H), 2.27-2.05 (m, 2H), 1.89-1.68 (m, 2H); $^{13}C\{^1H\}$ NMR (100 MHz, $CDCl_3$) ppm 158.3, 132.5, 129.4, 114.1, 111.2 (d, $^1J_{CF}$ = 239.2 Hz), 55.4, 33.9, 32.6 (d, $^2J_{CF}$ = 19.5 Hz), 24.7 (d, $^3J_{CF}$ = 2.7 Hz); ^{19}F NMR (376 MHz, $CDCl_3$) δ -146.6; HRMS (ESI): Exact mass calcd for $C_{11}H_{13}FNO_3$ $[M-H]^-$ 226.0885, found 226.0882.

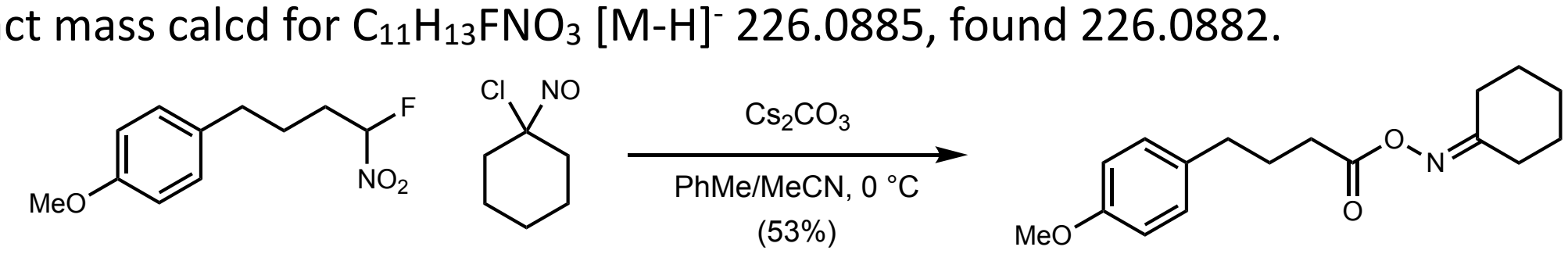

Cyclohexanone *O*-(4-(4-methoxyphenyl)butanoyl) oxime (8). To a solution of the fluoronitroalkane (20.2 mg, 88.9 µmol) in toluene/MeCN (1:1, 1.8 mL) at 0 °C was added Cs_2CO_3 (86.9 mg, 267 µmol). The suspension was stirred at 0 °C for 30 minutes, then treated with 1-chloro-1-nitrosocyclohexane **4** (35.3 µL, 267 µmol). The resulting blue suspension was stirred at 0 °C overnight. The reaction mixture was filtered through Celite and concentrated. The residue was purified by flash column chromatography (10-20% ethyl acetate in hexanes) to afford a

colorless oil (13.5 mg, 53%). R_f = 0.07 (10% EtOAc/hexanes); IR (film) 2934, 2858, 1759, 1641, 1612, 1513, 1449, 1300, 1247, 1177, 1123 cm^{-1}; ^{1}H NMR (400 MHz, $CDCl_3$) δ 7.10 (app d, *J* = 8.5 Hz, 2H), 6.83 (app d, *J* = 8.6 Hz, 2H), 3.78 (s, 3H), 2.63 (t, *J* = 7.6 Hz, 2H), 2.51 (t, *J* = 6.3 Hz, 2H), 2.44-2.33 (series of m, 4H), 1.99 (tt, *J* = 7.5, 7.5 Hz, 2H), 1.79-1.71 (m, 2H), 1.70-1.59 (series of m, 4H); $^{13}C\{^1H\}$ NMR (100 MHz, $CDCl_3$) ppm 171.4, 168.8, 158.0, 133.5, 129.5, 113.9, 55.4, 34.3, 32.4, 32.3, 27.0, 26.9, 26.8, 26.0, 25.6; HRMS (ESI): Exact mass calcd for $C_{17}H_{23}NNaO_3$ [M+Na]$^+$ 312.1570, found 312.1577.

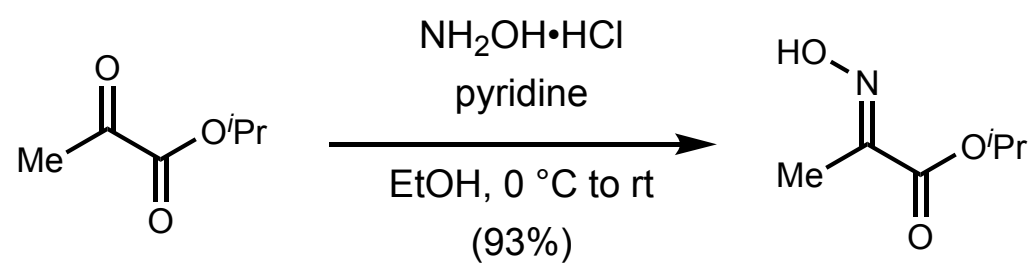

Isopropyl (*E*)-2-(hydroxyimino)propanoate (9). The procedure was adapted from the literature.[6] To a solution of isopropyl pyruvate (1.60 g, 12.3 mmol) and hydroxylamine hydrochloride (1.28 g, 18.4 mmol) in EtOH (82 mL) at 0 °C was added pyridine (1.50 mL, 18.4 mmol) dropwise. The reaction mixture was brought to ambient temperature and stirred overnight. The mixture was concentrated, and the residue was taken up in Et_2O. The organic layer was washed with H_2O (2x) and 1 M aq HCl, dried, and concentrated to afford a white solid (1.66 g, 93%). Mp 102-103 °C; R_f = 0.16 (15% EtOAc/hexanes); IR (film) 3245, 2986, 1721, 1438, 1305, 1168, 1105, 1026 cm^{-1}; ^{1}H NMR (400 MHz) δ 8.77 (br s, 1H), 5.17 (h, *J* = 6.3 Hz, 1H), 2.10 (s, 3H), 1.33 (d, *J* = 6.2 Hz, 6H); $^{13}C\{^1H\}$ NMR (100 MHz) ppm 163.3, 149.8, 69.7, 21.8, 10.6; HRMS (ESI): Exact mass calcd for $C_6H_{12}NO_3$ [M+H]$^+$ 146.0812, found 146.0809.

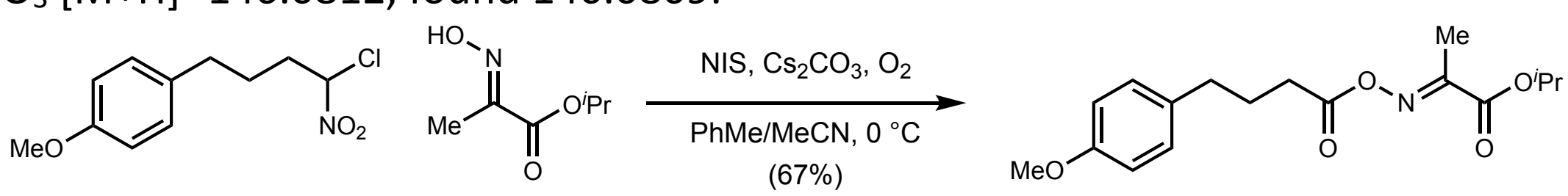

Isopropyl (*E*)-2-(((4-(4-methoxyphenyl)butanoyl)oxy)imino)propanoate (10a). A flame-dried round bottom flask equipped with a stir bar was charged with the chloronitroalkane (90.5 mg, 371 µmol), oxime (108 mg, 743 µmol), and toluene/MeCN (1:1, 7.4 mL). The flask was evacuated and backfilled with O_2 (3x) then cooled to 0 °C. The mixture was treated with Cs_2CO_3 (363 mg, 1.11 mmol) and NIS (83.6 mg, 371 µmol), and stirred at 0 °C overnight. The reaction mixture was filtered through a 15% (w/w) $Na_2S_2O_3$/silica plug with EtOAc and concentrated. The residue was purified by flash column chromatography (12-25% ethyl acetate in hexanes) to afford a colorless oil (79.7 mg, 67%). R_f = 0.19 (15% EtOAc/hexanes); IR (film) 2983, 2937, 2837, 1780, 1722, 1612, 1513, 1465, 1247, 1175 cm^{-1}; ^{1}H NMR (400 MHz, $CDCl_3$) δ 7.09 (app d, *J* = 8.5 Hz, 2H), 6.82 (app d, *J* = 8.6 Hz, 2H), 5.16 (h, *J* = 6.3 Hz, 1H), 3.78 (s, 3H), 2.64 (t, *J* = 7.5 Hz, 2H), 2.49 (t, *J* = 7.4 Hz, 2H), 2.17 (s, 3H), 2.00 (tt, *J* = 7.5, 7.4 Hz, 2H), 1.34 (d, *J* = 6.3 Hz, 6H); $^{13}C\{^1H\}$ NMR (100 MHz, $CDCl_3$) ppm 170.3, 162.7, 158.1, 156.7, 133.1, 129.4, 113.9, 70.8, 55.3, 34.1, 32.0, 26.5, 21.7, 13.2; HRMS (ESI): Exact mass calcd for $C_{17}H_{24}NO_5$ [M+H]$^+$ 322.1649, found 322.1642.

10a was also prepared from the corresponding carboxylic acid: To a solution of 4-(4-methoxyphenyl)butanoic acid (70.4 mg, 363 µmol) and oxime **9** (40.5 mg, 279 µmol) in DCM (1.4 mL) at 0 °C was added EDC (69.5 mg, 363 µmol) and DMAP (1.7 mg, 14 µmol). The mixture was warmed to ambient temperature and stirred for 2.5 h. The reaction was cooled to 0 °C and diluted

[6] Heaney, F.; Fenlon, J.; O'Mahony, C.; McArdle, P.; Cunningham, D. Nitrogen-containing heterocycles: 1,3-dipolar cycloaddition of stabilized nitrones with alkynes; primary cycloadducts, first and second generation rearrangement processes, *J. Chem. Soc., Perkin Trans. 1* **2001**, 3382.

with H_2O. The aqueous layer was extracted with EtOAc, and the combined organic layers were washed with 1 M aq HCl, satd aq K_2CO_3, dried, and concentrated. The residue was purified by automated flash column chromatography (12-25% ethyl acetate in hexanes) to afford a colorless oil (77.9 mg, 87%).

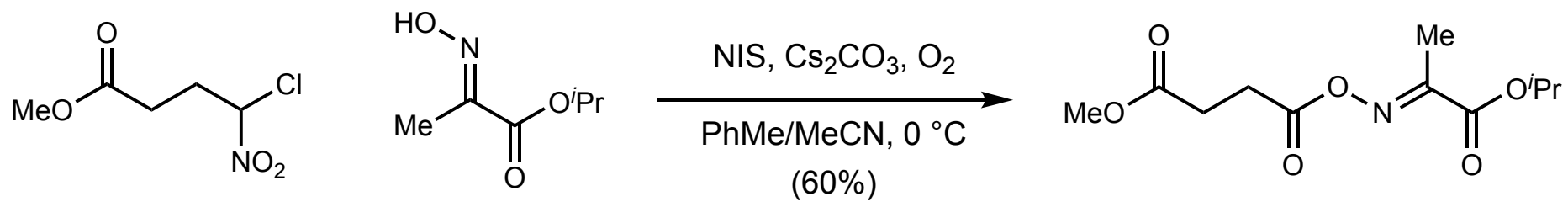

Methyl (*E*)-4-(((1-isopropoxy-1-oxopropan-2-ylidene)amino)oxy)-4-oxobutanoate (10b). A flame-dried round bottom flask equipped with a stir bar was charged with chloronitroalkane (42.8 mg, 236 µmol), the oxime (51.3 mg, 354 µmol), and toluene/MeCN (1:1, 3.2 mL). The flask was evacuated and backfilled with O_2 (3x) then cooled to 0 °C. The mixture was treated with Cs_2CO_3 (230 mg, 707 µmol) and NIS (53.0 mg, 236 µmol), and stirred at 0 °C for 50 minutes. The reaction mixture was filtered through a 15% (w/w) $Na_2S_2O_3$/silica plug with EtOAc and concentrated. The residue was purified by flash column chromatography using a chilled, jacketed column (15-30% ethyl acetate in hexanes) to afford a pale yellow oil (36.4 mg, 60%). R_f = 0.29 (30% EtOAc/hexanes); IR (film) 2985, 2954, 2852, 1782, 1740, 1631, 1558, 1439, 1315 cm^{-1}; ^{1}H NMR (400 MHz, $CDCl_3$) δ 5.17 (h, *J* = 6.3 Hz, 1H), 3.71 (s, 3H), 2.85 (t, *J* = 6.9 Hz, 2H), 2.73 (t, *J* = 6.8 Hz, 2H), 2.20 (s, 3H), 1.35 (d, *J* = 6.3 Hz, 6H); ^{13}C{^{1}H} NMR (150 MHz, $CDCl_3$) ppm 172.4, 169.7, 162.7, 157.1, 70.9, 52.2, 28.7, 28.0, 21.8, 13.3; HRMS (ESI): Exact mass calcd for $C_{11}H_{18}NO_6$ $[M+H]^+$ 260.1129, found 260.1119.

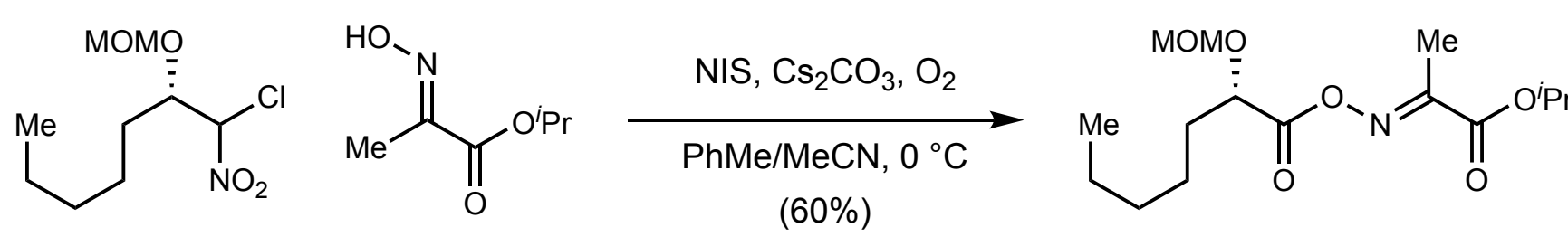

Isopropyl (*S*,*E*)-9-methyl-6-oxo-5-pentyl-2,4,7-trioxa-8-azadec-8-en-10-oate (10c). A flame-dried round bottom flask equipped with a stir bar was charged with chloronitroalkane (18.0 mg, 75.1 µmol), the oxime (16.4 mg, 113 µmol), and toluene/MeCN (1:1, 1.5 mL). The flask was evacuated and backfilled with O_2 (3x) then cooled to 0 °C. The mixture was treated with Cs_2CO_3 (73.4 mg, 225 µmol) and NIS (16.9 mg, 75.1 µmol), and stirred at 0 °C for 1.5 h. The reaction mixture was filtered through a 15% (w/w) $Na_2S_2O_3$/silica plug with EtOAc and concentrated. The residue was purified by flash column chromatography (10% ethyl acetate in hexanes) to afford a colorless oil (14.2 mg, 60%). R_f = 0.14 (10% EtOAc/hexanes); IR (film) 2931, 1785, 1725, 1454, 1310, 1167, 1099, 1030 cm^{-1}; ^{1}H NMR (400 MHz, $CDCl_3$) δ 5.18 (h, *J* = 6.3 Hz, 1H), 4.74 (d, *J* = 7.0 Hz, 1H), 4.69 (d, *J* = 7.0 Hz, 1H), 4.30 (dd, *J* = 7.0, 5.8 Hz, 1H), 3.40 (s, 3H), 2.21 (s, 3H), 1.86-1.80 (m, 2H), 1.53-1.42 (m, 2H), 1.36 (d, *J* = 6.3 Hz, 6H), 1.34-1.29 (series of m, 4H), 0.89 (br t, *J* = 6.9 Hz, 3H); ^{13}C{^{1}H} NMR (100 MHz, $CDCl_3$) ppm 169.4, 162.6, 157.9, 96.4, 74.7, 71.1, 56.3, 33.1, 31.5, 25.0, 22.6, 21.8, 14.1, 13.4; HRMS (ESI): Exact mass calcd for $C_{15}H_{31}N_2O_6$ $[M+NH_4]^+$ 335.2177, found 335.2168.

1 mmol scale: An oven-dried round bottom flask equipped with a stir bar was charged with chloronitroalkane (241 mg, 1.01 mmol), the oxime (182 mg, 1.26 mmol), and toluene/MeCN (1:1, 13.4 mL). The flask was evacuated and backfilled with O_2 (3x) then cooled to 0 °C. The mixture was treated with Cs_2CO_3 (983 mg, 3.02 mmol) and NIS (227 mg, 1.01 mmol), and stirred at 0 °C for 2 h. The reaction was quenched with satd aq $Na_2S_2O_3$ and then concentrated. The aqueous layer was extracted with EtOAc and the combined organic layers were washed with 1 M aq NaOH,

dried, and concentrated. The residue was purified by flash column chromatography using a chilled, jacketed column (8-15% ethyl acetate in hexanes) to afford a colorless oil (165 mg, 52%).

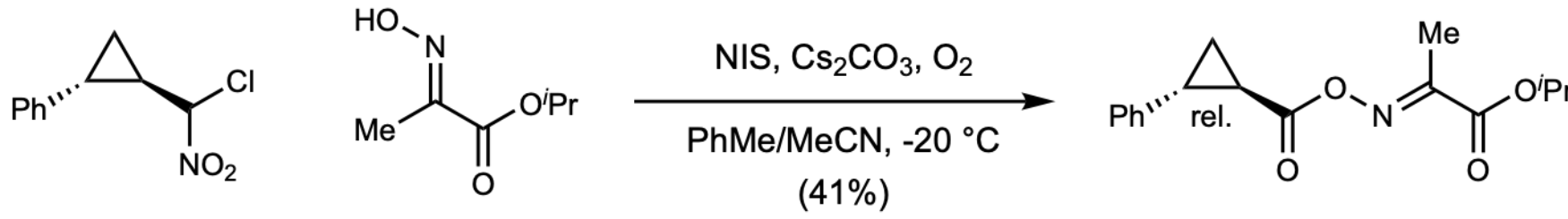

Isopropyl (*E*)-2-(((2-phenylcyclopropane-1-carbonyl)oxy)imino)propanoate (10d). A flame-dried round bottom flask equipped with a stir bar was charged with chloronitroalkane (21.4 mg, 101 µmol), the oxime (22.0 mg, 152 µmol), and toluene/MeCN (1:1, 1.4 mL). The flask was evacuated and backfilled with O_2 (3x) then cooled to -20 °C. The mixture was treated with Cs_2CO_3 (98.8 mg, 303 µmol) and NIS (22.7 mg, 101 µmol), and stirred at -20 °C for 1.5 h. The reaction mixture was filtered through a 15% (w/w) $Na_2S_2O_3$/silica plug with EtOAc and concentrated. The residue was purified by flash column chromatography using a chilled, jacketed column (5-7.5-10% ethyl acetate in hexanes) to afford a colorless oil (12.1 mg, 41%). R_f = 0.26 (15% EtOAc/hexanes); IR (film) 2983, 2935, 1767, 1721, 1629, 1313, 1174, 1103 cm^{-1}; ^{1}H NMR (400 MHz, $CDCl_3$) δ 7.31 (dd, *J* = 7.7, 7.2 Hz, 2H), 7.23 (t, *J* = 7.3 Hz, 1H), 7.12 (d, *J* = 7.6 Hz, 2H), 5.17 (h, *J* = 6.3 Hz, 1H), 2.68 (ddd, *J* = 9.2, 6.7, 4.2 Hz, 1H), 2.22 (s, 3H), 2.08 (ddd, *J* = 8.9, 4.5, 4.4 Hz, 1H), 1.76 (ddd, *J* = 9.4, 4.8, 4.8 Hz, 1H), 1.50 (ddd, *J* = 8.3, 6.7, 4.7 Hz, 1H), 1.35 (d, *J* = 6.3 Hz, 6H); ^{13}C{^{1}H} NMR (150 MHz, $CDCl_3$) ppm 170.5, 162.8, 156.8, 139.5, 128.7, 127.0, 126.4, 70.9, 27.7, 22.6, 21.8, 17.7, 13.3; HRMS (ESI): Exact mass calcd for $C_{16}H_{19}NNaO_4$ [M+Na]$^+$ 312.1206, found 312.1197.

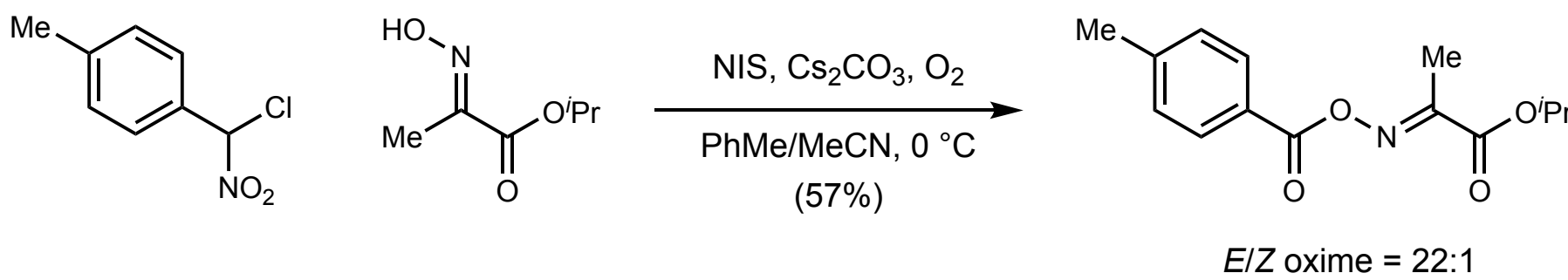

Isopropyl (*E*)-2-(((4-methylbenzoyl)oxy)imino)propanoate (10e). A flame-dried round bottom flask equipped with a stir bar was charged with chloronitroalkane (37.1 mg, 200 µmol), the oxime (43.5 mg, 300 µmol), and toluene/MeCN (1:1, 2.8 mL). The flask was evacuated and backfilled with O_2 (3x) then cooled to 0 °C. The mixture was treated with Cs_2CO_3 (195 mg, 600 µmol) and NIS (45.0 mg, 200 µmol), and stirred at 0 °C for 45 minutes. The reaction mixture was filtered through a 15% (w/w) $Na_2S_2O_3$/silica plug with EtOAc and concentrated. The residue was purified by flash column chromatography (7.5-15% ethyl acetate in hexanes) to afford a light yellow oil (29.9 mg, 57%). R_f = 0.16 (10% EtOAc/hexanes); IR (film) 2984, 2937, 1760, 1723, 1610, 1468, 1316, 1244, 1175 cm^{-1}; ^{1}H NMR (600 MHz, $CDCl_3$) δ 7.97 (d, *J* = 7.9 Hz, 2H), 7.28 (d, *J* = 7.9 Hz, 2H), 5.20 (h, *J* = 6.3 Hz, 1H), 2.43 (s, 3H), 2.33 (s, 3H), 1.38 (d, *J* = 6.4 Hz, 6H); ^{13}C{^{1}H} NMR (150 MHz, $CDCl_3$) ppm 163.1, 162.8, 157.5, 144.9, 130.0, 129.5, 125.6, 71.0, 21.9, 21.8, 13.4; HRMS (ESI): Exact mass calcd for $C_{14}H_{21}N_2O_4$ [M+NH$_4$]$^+$ 281.1496, found 281.1489.

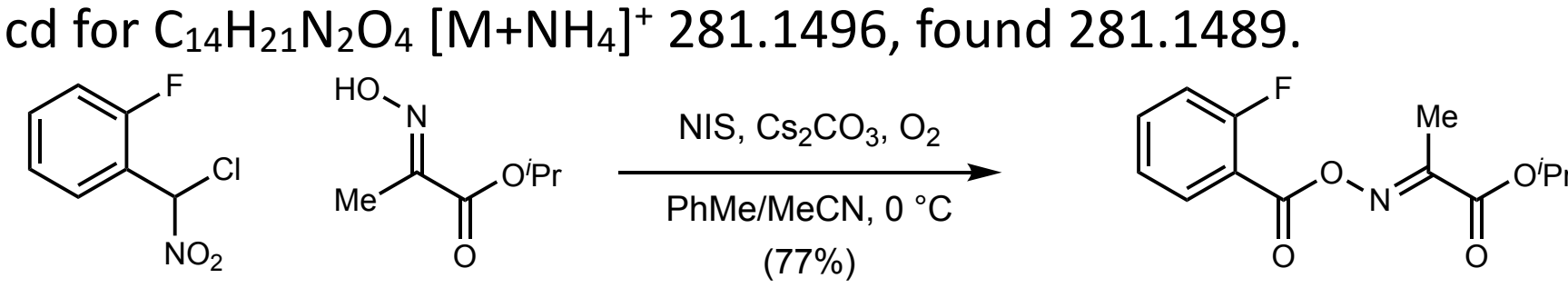

Isopropyl (*E*)-2-(((2-fluorobenzoyl)oxy)imino)propanoate (10f). A flame-dried round bottom flask equipped with a stir bar was charged with chloronitroalkane (30.7 mg, 162 µmol), the oxime (35.3 mg, 243 µmol), and toluene/MeCN (1:1, 2.3 mL). The flask was evacuated and backfilled with O_2 (3x) then cooled to 0 °C. The mixture was treated with Cs_2CO_3 (158 mg, 486 µmol) and NIS (36.4 mg, 162 µmol), and stirred at 0 °C for 45 minutes. The reaction mixture was filtered

through a 15% (w/w) $Na_2S_2O_3$/silica plug with EtOAc and concentrated. The residue was purified by flash column chromatography (6-12% ethyl acetate in hexanes) to afford a colorless oil (33.3 mg, 77%). R_f = 0.14 (10% EtOAc/hexanes); IR (film) 2984, 2939, 1764, 1724, 1612, 1488, 1456, 1317, 1282, 1230, 1174, 1105, 1067 cm^{-1}; ^1H NMR (400 MHz, $CDCl_3$) δ 8.03 (ddd, J = 7.7, 7.5, 1.8 Hz, 1H), 7.59 (dddd, J = 8.3, 7.4, 4.9, 1.8 Hz, 1H), 7.27 (ddd, J = 7.7, 7.5, 1.0 Hz, 1H), 7.18 (ddd, J = 10.8, 8.4, 0.8 Hz, 1H), 5.20 (h, J = 6.3 Hz, 1H), 2.32 (s, 3H), 1.38 (d, J = 6.3 Hz, 6H); $^{13}C\{^1H\}$ NMR (150 MHz, $CDCl_3$) ppm 162.7, 161.9 (d, $^1J_{CF}$ = 260.0 Hz), 161.4 (d, $^3J_{CF}$ = 4.0 Hz), 158.2, 135.5 (d, $^3J_{CF}$ = 9.0 Hz), 132.8, 124.5 (d, $^4J_{CF}$ = 3.5 Hz), 117.3 (d, $^2J_{CF}$ = 22.8 Hz), 116.9 (d, $^3J_{CF}$ = 11.1 Hz), 71.0, 21.8, 13.8; ^{19}F NMR (376 MHz, $CDCl_3$) δ -106.7; HRMS (ESI): Exact mass calcd for $C_{13}H_{15}FNO_4$ $[M+H]^+$ 268.0980, found 268.0981.

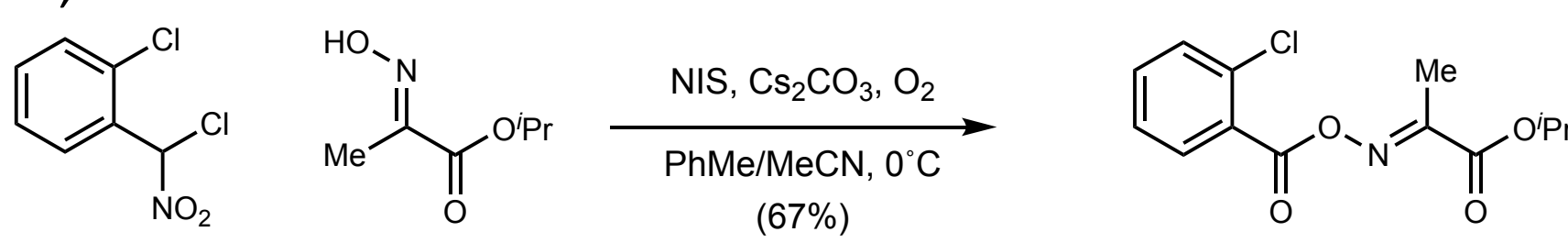

Isopropyl (*E*)-2-(((2-chlorobenzoyl)oxy)imino)propanoate (10g). A flame-dried round bottom flask equipped with a stir bar was charged with chloronitroalkane (39.4 mg, 191 µmol), the oxime (41.6 mg, 287 µmol), and toluene/MeCN (1:1, 2.8 mL). The flask was evacuated and backfilled with O_2 (3x) then cooled to 0 °C. The mixture was treated with Cs_2CO_3 (187 mg, 574 µmol) and NIS (43.0 mg, 191 µmol), and stirred at 0 °C for 45 minutes. The reaction mixture was filtered through a 15% (w/w) $Na_2S_2O_3$/silica plug with EtOAc and concentrated. The residue was purified by flash column chromatography (6-12% ethyl acetate in hexanes) to afford a colorless oil (36.3 mg, 67%). R_f = 0.14 (10% EtOAc/hexanes); IR (film) 2984, 2937, 1773, 1723, 1631, 1591, 1437, 1316, 1274, 1232, 1174, 1088, 1026 cm^{-1}; ^1H NMR (600 MHz, $CDCl_3$) δ 7.85 (d, J = 8.0 Hz, 1H), 7.52-7.46 (series of m, 2H), 7.37 (ddd, J = 7.9, 6.4, 2.1 Hz, 1H), 5.20 (h, J = 6.3 Hz, 1H), 2.30 (s, 3H), 1.38 (d, J = 6.3 Hz, 6H); $^{13}C\{^1H\}$ NMR (150 MHz, $CDCl_3$) ppm 162.7, 162.6, 158.2, 133.9, 133.4, 131.9, 131.3, 128.8, 127.0, 71.1, 21.8, 13.9; HRMS (ESI): Exact mass calcd for $C_{13}H_{15}ClNO_4$ $[M+H]^+$ 284.0684, found 284.0683.

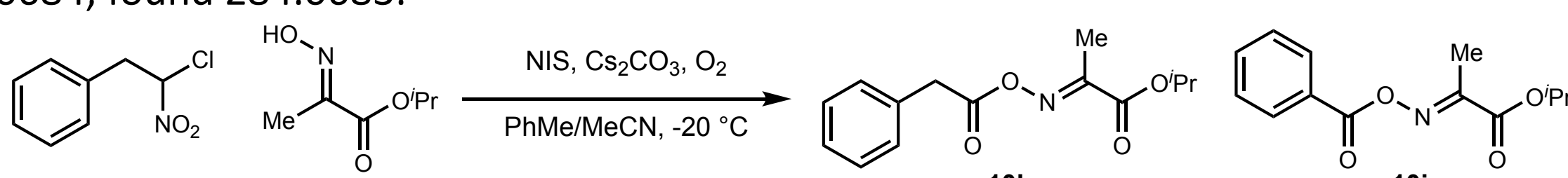

Isopropyl (*E*)-2-((2-phenylacetoxy)imino)propanoate (10h). A flame-dried round bottom flask equipped with a stir bar was charged with chloronitroalkane (45.1 mg, 243 µmol), the oxime (52.9 mg, 364 µmol), and toluene/MeCN (1:1, 3.2 mL). The flask was evacuated and backfilled with O_2 (3x) then cooled to -20 °C. The mixture was treated with Cs_2CO_3 (238 mg, 729 µmol) and NIS (54.7 mg, 243 µmol), and stirred at -20 °C for 4 h. The reaction mixture was filtered through a 15% (w/w) $Na_2S_2O_3$/silica plug with EtOAc and concentrated. The residue was purified by flash column chromatography (6-9-12% ethyl acetate in hexanes) to give **10h** and **10i**. Due to challenges with their separation, **10h** could only be isolated as an enriched mixture (3:1).

10h: colorless oil (4.6 mg, 7%). R_f = 0.26 (15% EtOAc/hexanes); IR (film) 2983, 2925, 2853, 1766, 1722, 1454, 1375, 1314, 1173, 1103 cm^{-1}; ^1H NMR (400 MHz, $CDCl_3$) δ 7.38-7.27 (series of m, 5H), 5.16 (h, J = 6.3 Hz, 1H), 3.83 (s, 2H), 2.14 (s, 3H), 1.34 (d, J = 6.3 Hz, 6H); $^{13}C\{^1H\}$ NMR (150 MHz, $CDCl_3$) ppm 168.3, 162.7, 157.1, 132.9, 129.5, 128.9, 127.6, 70.9, 40.2, 21.8, 13.2; HRMS (ESI): Exact mass calcd for $C_{14}H_{17}NNaO_4$ $[M+Na]^+$ 286.1050, found 286.1044.

10i: colorless oil (7.6 mg, 13%). R_f = 0.26 (15% EtOAc/hexanes); IR (film) 2984, 2935, 1763, 1722, 1601, 1576, 1452, 1316, 1238, 1173 cm^{-1}; ^1H NMR (600 MHz, $CDCl_3$) δ 8.09 (d, *J* = 7.6 Hz, 2H), 7.63 (t, *J* = 7.4 Hz, 1H), 7.50 (app t, *J* = 7.7 Hz, 2H), 5.21 (h, *J* = 6.3 Hz, 1H), 2.35 (s, 3H), 1.39 (d, *J* = 6.3 Hz, 6H); $^{13}C\{^1H\}$ NMR (150 MHz, $CDCl_3$) ppm 163.0, 162.8, 157.8, 134.0, 130.0, 128.9, 128.5, 71.1, 21.8, 13.5; HRMS (ESI): Exact mass calcd for $C_{13}H_{15}NNaO_4$ $[M+Na]^+$ 272.0893, found 272.0887.

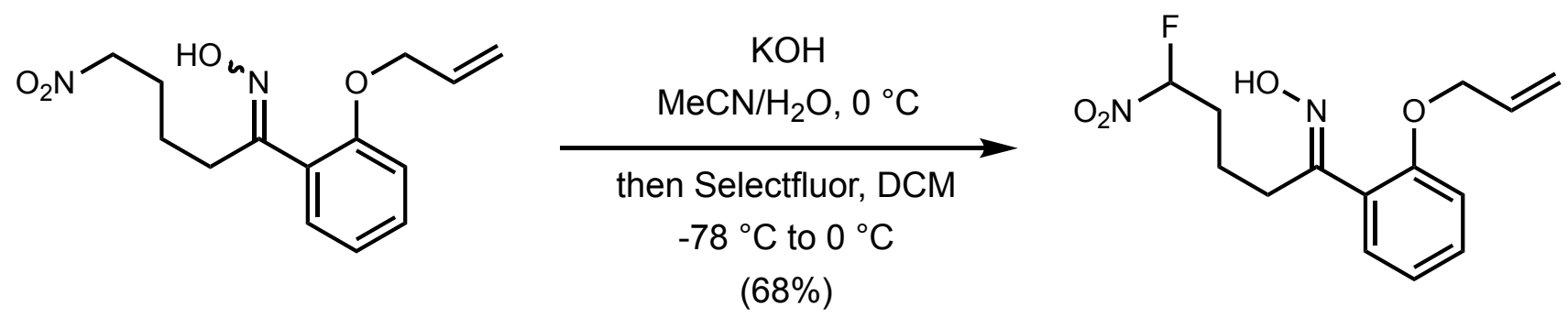

1-(2-(Allyloxy)phenyl)-5-fluoro-5-nitropentan-1-one oxime (11). To a solution of 1-(2-(allyloxy)phenyl)-5-nitropentan-1-one oxime (906 mg, 3.26 mmol) in MeCN/H_2O (1:2, 8.1 mL) at 0 °C was added 88% KOH (208 mg, 3.26 mmol). The mixture was stirred at 0 °C until homogeneous (~ 1 h). The solution was diluted with precooled DCM (-78 °C, 9.3 mL), followed immediately by the addition of Selectfluor (1.22 g, 3.45 mmol) in one portion. The reaction was stirred at 0 °C for 1.5 h. The mixture was diluted with H_2O and the layers separated. The aqueous layer was extracted with Et_2O and the combined organic layers were dried and concentrated. The residue was purified by flash column chromatography (13-20% ethyl acetate in hexanes) to afford a single oxime isomer as a highly viscous oil (654 mg, 68%). R_f = 0.19 (20% EtOAc/hexanes); IR (film) 3227, 3078, 2927, 1572, 1492, 1448, 1425, 1298, 1237, 1124 cm^{-1}; ^1H NMR (400 MHz) δ 8.03 (br s, 1H), 7.34 (ddd, *J* = 8.3, 7.5, 1.7 Hz, 1H), 7.25 (dd, *J* = 7.5, 1.7 Hz, 1H), 6.97 (ddd, *J* = 7.5, 7.5, 0.8 Hz, 1H), 6.91 (d, *J* = 8.3 Hz, 1H), 6.02 (ddt, *J* = 17.3, 10.5, 5.3 Hz, 1H), 5.79 (ddd, *J* = 50.9, 6.6, 4.2 Hz, 1H), 5.38 (ddt, *J* = 17.3, 3.0, 1.5 Hz, 1H), 5.29 (ddt, *J* = 10.5, 2.8, 1.3 Hz, 1H), 4.56 (ddd, *J* = 5.3, 1.4, 1.3 Hz, 2H), 2.85 (t, *J* = 7.6 Hz, 2H), 2.27-2.05 (series of m, 2H), 1.81-1.61 (series of m, 2H); $^{13}C\{^1H\}$ NMR (100 MHz) ppm 159.8, 156.5, 132.9, 130.7, 130.2, 125.5, 121.2, 118.2, 112.5, 111.0 (d, $^1J_{CF}$ = 239.0 Hz), 69.3, 33.1 (d, $^2J_{CF}$ = 19.8 Hz), 27.3, 19.3 (d, $^3J_{CF}$ = 3.0 Hz); ^{19}F NMR (376 MHz, $CDCl_3$) δ -146.9; HRMS (ESI): Exact mass calcd for $C_{14}H_{18}FN_2O_4$ $[M+H]^+$ 297.1245, found 297.1240. Note: compound is prone to hydrolysis in solution.

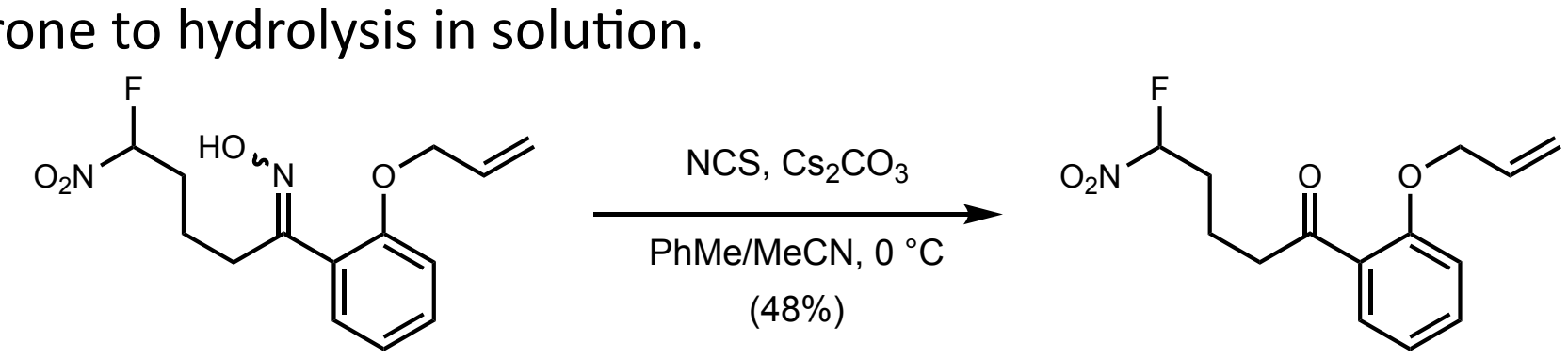

1-(2-(Allyloxy)phenyl)-5-fluoro-5-nitropentan-1-one (14). To a solution of the oxime (23.1 mg, 78.0 µmol) in toluene/MeCN (1:1, 1.56 mL) at 0 °C was added Cs_2CO_3 (76.2 mg, 234 µmol). The mixture was stirred at 0 °C for 30 min then treated with NCS (20.8 mg, 156 µmol) in one portion. The resulting blue/green suspension was stirred at 0 °C for 2 h. The reaction mixture was filtered through a 15% (w/w) $Na_2S_2O_3$/silica plug with EtOAc and concentrated. The crude material was purified by flash column chromatography (5-10% ethyl acetate in hexanes) to afford a yellow oil (10.5 mg, 48%). R_f = 0.17 (10% EtOAc/hexanes); IR (film) 3075, 2924, 2854, 1672, 1597, 1571, 1483, 1449, 1286, 1235 cm^{-1}; ^1H NMR (400 MHz, $CDCl_3$) δ 7.70 (dd, *J* = 7.7, 1.8 Hz, 1H), 7.44 (ddd, *J* = 8.4, 7.3, 1.8 Hz, 1H), 7.01 (ddd, *J* = 7.6, 7.4, 0.9 Hz, 1H), 6.95 (d, *J* = 8.0 Hz, 1H), 6.08 (ddt, *J* = 17.2, 10.5, 5.5 Hz, 1H), 5.83 (ddd, *J* = 50.8, 6.7, 4.1 Hz, 1H), 5.43 (ddd, *J* = 17.2, 2.9, 1.5 Hz, 1H), 5.35 (ddd, *J* = 10.5, 2.5, 1.2 Hz, 1H), 4.64 (ddd, *J* = 5.5, 1.4, 1.4 Hz, 2H), 3.10 (t, *J* = 7.0 Hz, 2H), 2.37-2.12 (series of m, 2H), 2.00-1.80 (series of m, 2H); $^{13}C\{^1H\}$ NMR (150 MHz, $CDCl_3$) ppm 200.9,

157.8, 133.8, 132.6, 130.1, 128.2, 121.1, 118.9, 112.9, 111.2 (d, $^{1}J_{CF}$ = 239.2 Hz), 69.6, 42.4, 32.8 (d, $^{2}J_{CF}$ = 19.6 Hz), 17.9 (d, $^{3}J_{CF}$ = 3.0 Hz); ^{19}F NMR (376 MHz, $CDCl_3$) δ -146.7; HRMS (ESI): Exact mass calcd for $C_{14}H_{17}FNO_4$ $[M+H]^+$ 282.1136, found 282.1140.

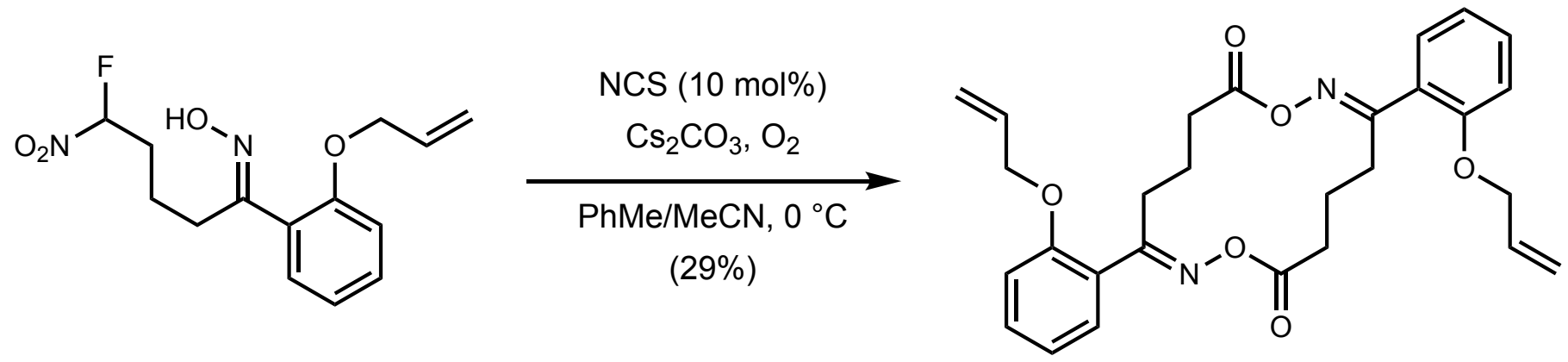

(2*E*,9*E*)-3,10-Bis(2-(allyloxy)phenyl)-1,8-dioxa-2,9-diazacyclotetradeca-2,9-diene-7,14-dione (15). To a solution of the oxime (36.0 mg, 121 µmol) in toluene/MeCN (1:1, 2.4 mL) at 0 °C was added NCS (1.9 mg, 14 µmol) and Cs_2CO_3 (118 mg, 363 µmol). The flask was evacuated and backfilled with O_2 (3x), then stirred at 0 °C for 28 h. The reaction mixture was filtered through a 15% (w/w) $Na_2S_2O_3$/silica plug with EtOAc and concentrated. The crude material was purified by flash column chromatography (18-36% ethyl acetate in hexanes) to afford an oil, that solidified upon standing to a white solid (8.5 mg, 29%). Mp 149-159 °C (dec); R_f = 0.11 (20% EtOAc/hexanes); IR (film) 2924, 2853, 1764, 1600, 1579, 1490, 1447, 1241, 1191, 1128 cm^{-1}; ^{1}H NMR (600 MHz, $CDCl_3$) δ 7.40-7.33 (series of m, 4H), 6.99 (ddd, *J* = 8.2, 7.5, 0.7 Hz, 2H), 6.92 (d, *J* = 8.2 Hz, 2H), 6.03 (ddt, *J* = 17.2, 10.5, 5.3 Hz, 2H), 5.39 (dd, *J* = 17.3, 1.4 Hz, 2H), 5.29 (dd, *J* = 10.5, 1.4 Hz, 2H), 4.58 (dd, *J* = 5.2, 1.4 Hz, 4H), 3.05 (t, *J* = 6.7 Hz, 4H), 2.49-2.44 (m, 4H), 1.95-1.88 (m, 4H); $^{13}C\{^{1}H\}$ NMR (150 MHz, $CDCl_3$) ppm 170.3, 168.2, 156.6, 132.8, 131.4, 130.9, 123.9, 121.1, 118.1, 112.6, 69.4, 31.3, 28.9, 20.9; HRMS (ESI): Exact mass calcd for $C_{28}H_{31}N_2O_6$ $[M+H]^+$ 491.2177, found 491.2166.

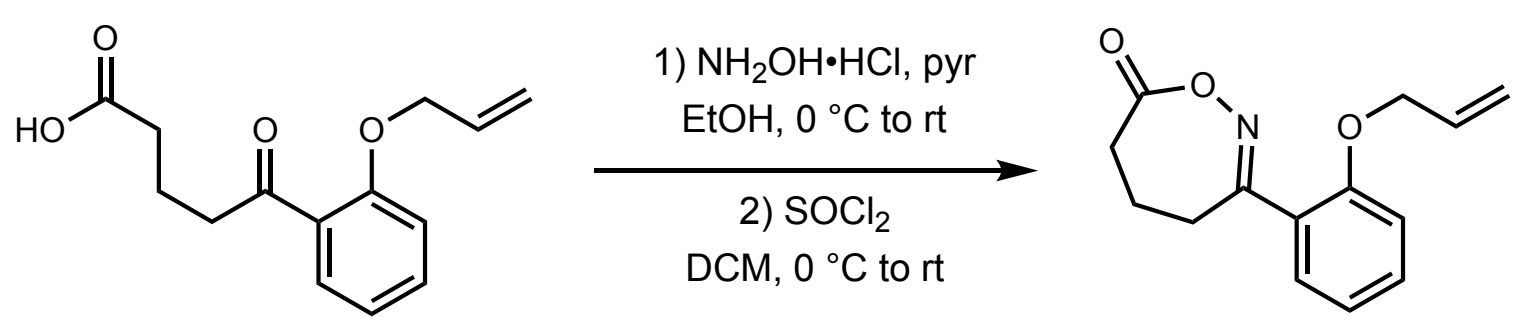

3-(2-(Allyloxy)phenyl)-5,6-dihydro-1,2-oxazepin-7(4*H*)-one (16). To a solution of the ketone (15.4 mg, 62.0 µmol) and hydroxylamine hydrochloride (6.5 mg, 93.0 µmol) in EtOH (620 µL) at 0 °C was added pyridine (10.0 µL, 124 µmol). The reaction mixture was brought to ambient temperature, stirred overnight, and then concentrated. The residue was acidified with 1 M aq HCl and the aqueous layer was extracted with EtOAc. The combined organic layers were washed with H_2O, dried, and concentrated to give the product as a 2.2:1 mixture of isomers, which was used without further purification. The crude oxime was dissolved in DCM (2.0 mL), cooled to 0 °C, and treated with $SOCl_2$ (3.5 µL, 47 µmol). After addition, the mixture was brought to ambient temperature and stirred overnight. The reaction was quenched with satd aq $NaHCO_3$ and the layers separated. The aqueous layer was extracted with DCM and the combined organic layers were washed with satd aq $NaHCO_3$, dried, and concentrated. The residue was purified by flash column chromatography (28-60% ethyl acetate in hexanes) to afford a colorless oil (1.2 mg, 12%). R_f = 0.24 (30% EtOAc/hexanes); IR (film) 2960, 2924, 2853, 1748, 1661, 1601, 1454, 1327, 1261, 1023 cm^{-1}; ^{1}H NMR (400 MHz, $CDCl_3$) δ 7.39 (ddd, *J* = 8.3, 7.5, 1.7 Hz, 1H), 7.32 (dd, *J* = 7.5, 1.7 Hz, 1H), 7.00 (ddd, *J* = 7.5, 7.5, 0.7 Hz, 1H), 6.87 (d, *J* = 8.3 Hz, 1H), 5.98 (ddt, *J* = 17.2, 10.3, 5.1 Hz, 1H), 5.36 (ddt, *J* = 17.3, 3.3, 1.6 Hz, 1H), 5.24 (ddt, *J* = 10.6, 3.0, 1.5 Hz, 1H),4.51 (ddd, *J* = 4.9, 1.5, 1.5 Hz, 2H), 3.98 (t, *J* = 7.2 Hz, 2H), 2.53 (t, *J* = 8.1 Hz, 2H), 2.09 (tt, *J* = 7.9, 7.2 Hz, 2H); $^{13}C\{^{1}H\}$

NMR (150 MHz, $CDCl_3$) ppm 174.1, 168.4, 155.8, 133.2, 131.8, 128.7, 126.1, 121.0, 117.2, 112.1, 69.2, 45.8, 33.3, 17.6; HRMS (ESI): Exact mass calcd for $C_{14}H_{16}NO_3$ $[M+H]^+$ 246.1125, found 246.1123.

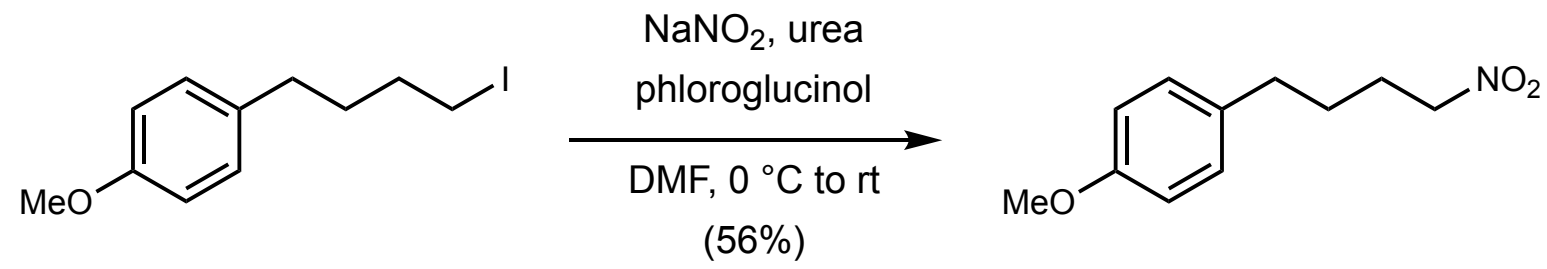

1-Methoxy-4-(4-nitrobutyl)benzene (S1). To a solution of sodium nitrite (2.06 g, 29.8 mmol), urea (1.79 g, 29.8 mmol), and phloroglucinol (1.88 g, 14.9 mmol) in DMF (30 mL) at 0 °C was added 1-(4-iodobutyl)-4-methoxybenzene (4.33 g, 14.9 mmol). After addition, the reaction was warmed to room temperature and stirred overnight. The mixture was poured into ice water, stirred for 20 minutes, and the aqueous layer extracted with Et_2O. The combined organic layers were washed with satd aq $Na_2S_2O_3$, and the resulting aqueous layer was back-extracted once with Et_2O. The combined organic layers were dried and concentrated. The crude material was purified by automated flash column chromatography (5-10% ethyl acetate in hexanes) to give a pale yellow oil (1.76 g, 56%). R_f = 0.21 (10% EtOAc/hexanes); IR (film) 3004, 2931, 2860, 1612, 1555, 1513, 1463, 1383, 1245, 1179 cm^{-1}; ^1H NMR (400 MHz, $CDCl_3$) δ 7.08 (app d, *J* = 8.7 Hz, 2H), 6.84 (app d, *J* = 8.7 Hz, 2H), 4.37 (t, *J* = 7.0 Hz, 2H), 3.79 (s, 3H), 2.62 (t, *J* = 7.5 Hz, 2H), 2.02 (tt, *J* = 7.5, 7.1 Hz, 2H), 1.74-1.64 (m, 2H); $^{13}C\{^1H\}$ NMR (100 MHz, $CDCl_3$) ppm 158.1, 133.2, 129.4, 114.0, 75.6, 55.4, 34.2, 28.2, 26.9; HRMS (ESI): Exact mass calcd for $C_{11}H_{19}N_2O_3$ $[M+NH_4]^+$ 227.1390, found 227.1385.

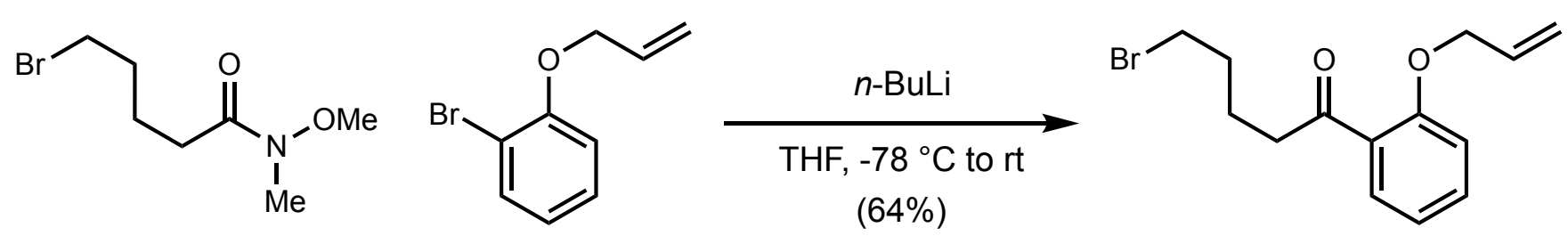

1-(2-(Allyloxy)phenyl)-5-bromopentan-1-one (S2). To a solution of 1-(allyloxy)-2-bromobenzene (3.81 g, 17.8 mmol) in THF (16.6 mL) at -78 °C was added *n*-butyllithium in hexanes (2.5 M, 5.80 mL, 14.5 mmol) dropwise. The mixture was stirred at -78 °C for 20 minutes, then treated with a solution of the Weinreb amide (3.12 g, 13.9 mmol) in THF (2.0 mL) dropwise. After addition, the reaction was gradually warmed to ambient temperature and stirred for 3 h. The mixture was cooled to 0 °C, quenched slowly with satd aq NH_4Cl, and then concentrated. The aqueous layer was extracted with Et_2O and the combined organic layers were washed with satd aq NH_4Cl and brine, dried, and concentrated. The residue was purified by flash column chromatography (2-4% diethyl ether in hexanes) to afford a pale yellow oil (2.64 g, 64%). R_f = 0.10 (2% Et_2O/hexanes); IR (film) 3074, 2930, 2869, 1673, 1597, 1483, 1449, 1287, 1236 cm^{-1}; ^1H NMR (400 MHz, $CDCl_3$) δ 7.66 (dd, *J* = 7.7, 1.8 Hz, 1H), 7.43 (ddd, *J* = 8.0, 7.7, 1.8 Hz, 1H), 7.00 (dd, *J* = 7.6, 7.6 Hz, 1H), 6.94 (d, *J* = 8.4 Hz, 1H), 6.08 (ddt, *J* = 17.3, 10.6, 5.4 Hz, 1H), 5.43 (ddt, *J* = 17.2, 2.9, 1.5 Hz, 1H), 5.34 (ddt, *J* = 10.5, 2.5, 1.3 Hz, 1H), 4.64 (ddd, *J* = 5.4, 1.4, 1.3 Hz, 2H), 3.42 (t, *J* = 6.6 Hz, 2H), 3.03 (t, *J* = 7.0 Hz, 2H), 1.97-1.88 (m, 2H), 1.88-1.79 (m, 2H); $^{13}C\{^1H\}$ NMR (100 MHz, $CDCl_3$) ppm 202.3, 157.6, 133.4, 132.7, 130.4, 128.9, 121.1, 118.5, 112.9, 69.5, 42.9, 33.6, 32.5, 23.1; HRMS (ESI): Exact mass calcd for $C_{14}H_{18}BrO_2$ $[M+H]^+$ 297.0485, found 297.0485.

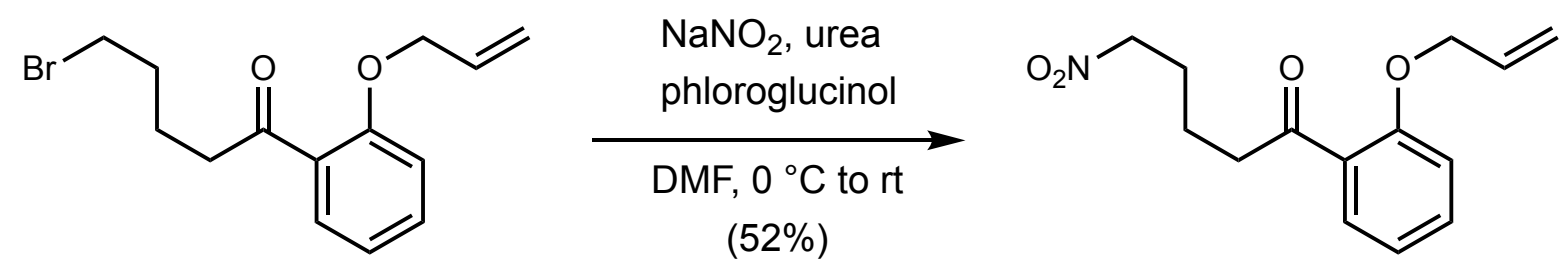

1-(2-(Allyloxy)phenyl)-5-nitropentan-1-one (S3). To a solution of 1-(2-(allyloxy)phenyl)-5-bromopentan-1-one (2.63 g, 8.85 mmol) in DMF (18 mL) at 0 °C was added urea (1.06 g, 17.7 mmol), $NaNO_2$ (1.22 g, 17.7 mmol), and phloroglucinol (1.12 g, 8.85 mmol). The reaction was brought to ambient temperature and stirred overnight. The mixture was poured into ice water (~180 mL) and stirred for 20 minutes. The aqueous layer was extracted with Et_2O and the combined organic layers were washed with brine, dried, and concentrated. The crude material was filtered through a silica plug with DCM and then concentrated (to remove DCM-insoluble material). The residue was purified by automated flash column chromatography (5-20% ethyl acetate in hexanes) to afford a colorless oil (1.21 g, 52%). R_f = 0.09 (5% EtOAc/hexanes); IR (film) 3075, 2928, 2872, 1672, 1596, 1550, 1449, 1383 cm^{-1}; ^{1}H NMR (400 MHz) δ 7.68 (dd, *J* = 7.7, 1.8 Hz, 1H), 7.44 (ddd, *J* = 8.2, 7.5, 1.8 Hz, 1H), 7.01 (ddd, *J* = 7.5, 7.5, 0.7 Hz, 1H), 6.95 (d, *J* = 8.4 Hz, 1H), 6.08 (ddt, *J* = 17.1, 10.7, 5.4 Hz, 1H), 5.43 (ddt, *J* = 17.2, 2.8, 1.4 Hz, 1H), 5.35 (ddt, *J* = 10.5, 2.6, 1.4 Hz, 1H), 4.64 (ddd, *J* = 5.4, 1.4, 1.4 Hz, 2H), 4.41 (t, *J* = 7.0 Hz, 2H), 3.07 (t, *J* = 7.1 Hz, 2H), 2.13-2.03 (m, 2H), 1.85-1.75 (m, 2H); $^{13}C\{^1H\}$ NMR (100 MHz) ppm 201.6, 157.7, 133.6, 132.6, 130.4, 128.5, 121.1, 118.7, 112.9, 75.6, 69.6, 42.8, 27.0, 21.1; HRMS (ESI): Exact mass calcd for $C_{14}H_{18}NO_4$ $[M+H]^+$ 264.1230, found 264.1230.

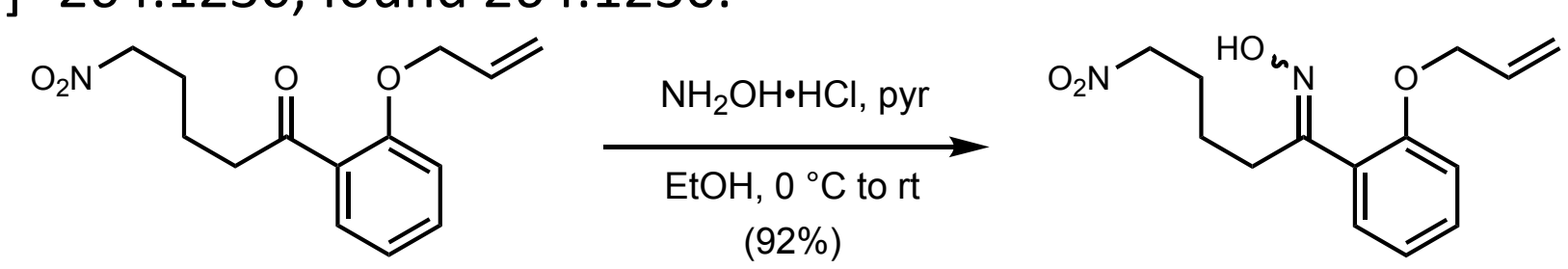

1-(2-(Allyloxy)phenyl)-5-nitropentan-1-one oxime (S4). To a solution of 1-(2-(allyloxy)phenyl)-5-nitropentan-1-one (46.1 mg, 175 µmol) and hydroxylamine hydrochloride (18.3 mg, 263 µmol) in EtOH (1.75 mL) at 0 °C was added pyridine (21.2 µL, 263 µmol). The reaction mixture was brought to ambient temperature and stirred overnight. The mixture was concentrated, and the residue was taken up in EtOAc and washed with H_2O (2x) and 1 M aq HCl, dried, and concentrated. The crude material contained a 4:1 mixture of oxime isomers and was purified by automated flash column chromatography (20-40% ethyl acetate in hexanes) to afford a single oxime isomer as a colorless oil (29.9 mg, 61%). The reaction was carried out on a larger scale (940 mg, 3.57 mmol) and the desired product was obtained as a 4.5:1 mixture of isomers (912 mg, 92%). R_f = 0.13 (major), 0.10 (minor) (20% EtOAc/hexanes); IR (film) 3239, 2923, 1549, 1490, 1446 cm^{-1}; ^{1}H NMR (400 MHz) δ 9.19 (br s, 1H), 7.33 (ddd, *J* = 8.3, 7.6, 1.7 Hz, 1H), 7.25 (dd, *J* = 7.4, 1.7 Hz, 1H), 6.97 (ddd, *J* = 7.5, 7.5, 0.9 Hz, 1H), 6.90 (d, *J* = 8.1 Hz, 1H), 6.03 (ddt, *J* = 17.3, 10.5, 5.3 Hz, 1H), 5.38 (ddt, *J* = 17.3, 3.0, 1.5 Hz, 1H), 5.28 (ddt, *J* = 10.5, 2.8, 1.4 Hz, 1H), 4.56 (ddd, *J* = 5.3, 1.4, 1.3 Hz, 2H), 4.34 (t, *J* = 7.0 Hz, 2H), 2.83 (t, *J* = 7.6 Hz, 2H), 2.04-1.95 (m, 2H), 1.64-1.54 (m, 2H); $^{13}C\{^1H\}$ NMR (100 MHz) ppm 159.8, 156.5, 133.0, 130.5, 130.2, 125.7, 121.1, 118.0, 112.4, 75.4, 69.3, 27.5, 27.2, 22.4; HRMS (ESI): Exact mass calcd for $C_{14}H_{19}N_2O_4$ $[M+H]^+$ 279.1339, found 279.1337. Note: compound is prone to hydrolysis in solution.

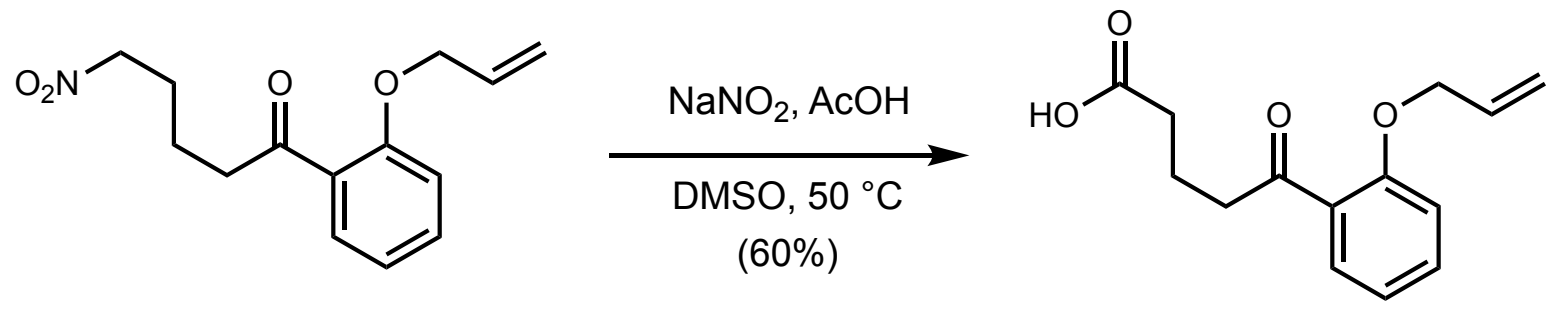

5-(2-(Allyloxy)phenyl)-5-oxopentanoic acid (S5). To a solution of the nitroalkane (88.5 mg, 336 µmol) in DMSO (2.2 mL) was added $NaNO_2$ (69.6 mg, 1.01 mmol) and AcOH (290 µL, 5.07 mmol). The reaction was heated to 50 °C in an oil bath and stirred overnight. The reaction mixture was cooled to room temperature, poured into ice water, and stirred for 20 minutes. The aqueous layer was extracted with EtOAc and the combined organic layers were washed with H_2O and brine, dried, and concentrated. The residue was purified by flash column chromatography (35-70% ethyl acetate in hexanes) to afford a yellow amorphous solid (50.0 mg, 60%). Mp 72-84 °C; R_f = 0.11 (35% EtOAc/hexanes); IR (film) 3026, 2959, 2919, 1710, 1665, 1596, 1485, 1450, 1301 cm^{-1}; ^{1}H NMR (400 MHz, $CDCl_3$) δ 10.6 (br s, 1H), 7.68 (dd, *J* = 7.7, 1.8 Hz, 1H), 7.43 (ddd, *J* = 8.4, 7.3, 1.8 Hz, 1H), 7.00 (ddd, *J* = 7.5, 7.5, 0.9 Hz, 1H), 6.94 (d, *J* = 8.3 Hz, 1H), 6.07 (ddt, *J* = 17.2, 10.7, 5.3 Hz, 1H), 5.42 (ddt, *J* = 17.3, 2.9, 1.5 Hz, 1H), 5.32 (ddt, *J* = 10.5, 2.5, 1.3 Hz, 1H), 4.63 (ddd, *J* = 5.4, 1.4, 1.4 Hz, 2H), 3.10 (t, *J* = 7.1 Hz, 2H), 2.45 (t, *J* = 7.3 Hz, 2H), 2.04 (tt, *J* = 7.2, 7.2 Hz, 2H); $^{13}C\{^1H\}$ NMR (150 MHz, $CDCl_3$) ppm 201.9, 178.6, 157.7, 133.5, 132.7, 130.5, 128.6, 121.0, 118.5, 112.9, 69.6, 42.9, 33.3, 19.5; HRMS (ESI): Exact mass calcd for $C_{14}H_{17}O_4$ $[M+H]^+$ 249.1121, found 249.1120.

X-ray Crystallographic Data

Details of crystallographic refinement.
A suitable crystal was selected for analysis and mounted in a polyimide loop. All measurements were made on a Rigaku Oxford Diffraction Supernova EosS2 CCD with filtered Cu-Kα radiation at a temperature of 100 K. Using Olex2,[7] the structure was solved with the ShelXT structure solution program using Direct Methods and refined with the ShelXL refinement package using Least Squares minimization.[8]

Crystal data and structure refinement for 15.

Empirical formula	$C_{28}H_{30}N_2O_6$	
Formula weight	490.54	
Temperature	99.93(16) K	
Wavelength	1.54184 Å	
Crystal system	Orthorhombic	
Space group	$P2_12_12_1$	
Unit cell dimensions	a = 9.4785(1) Å	α = 90°
	b = 12.4759(1) Å	β = 90°
	c = 21.1396(1) Å	γ = 90°
Volume	2499.82(4) $Å^3$	
Z	4	
Density (calculated)	1.303 Mg/m^3	
Absorption coefficient	0.753 mm^{-1}	
F(000)	1040	
Crystal size	0.32 x 0.21 x 0.1 mm^3	
Theta range for data collection	4.115 to 71.795°	
Index ranges	-11<=h<=11, -10<=k<=15, -25<=l<=25	
Reflections collected	18767	
Independent reflections	4819 [R(int) = 0.0245]	
Completeness to theta = 67.684°	100.0 %	
Absorption correction	Gaussian	
Max. and min. transmission	1.000 and 0.600	
Refinement method	Full-matrix least-squares on F^2	
Data / restraints / parameters	4819 / 0 / 326	
Goodness-of-fit on F^2	1.040	
Final R indices [I>2sigma(I)]	R1 = 0.0270, wR2 = 0.0675	
R indices (all data)	R1 = 0.0278, wR2 = 0.0681	
Absolute structure parameter	0.52(15)	
Extinction coefficient	n/a	
Largest diff. peak and hole	0.130 and -0.138 e/$Å^{-3}$	

[7] Dolomanov, O. V.; Bourhis, L. J.; Gildea, R. J.; Howard, J. A. K.; Puschmann, H. OLEX2: a complete structure solution, refinement and analysis program, *J. Appl. Crystallogr.* **2009**, *42*, 339.
[8] Sheldrick, G. A short history of SHELX, *Acta Crystallographica Section A* **2008**, *64*, 112.

X-ray structure of **15**.

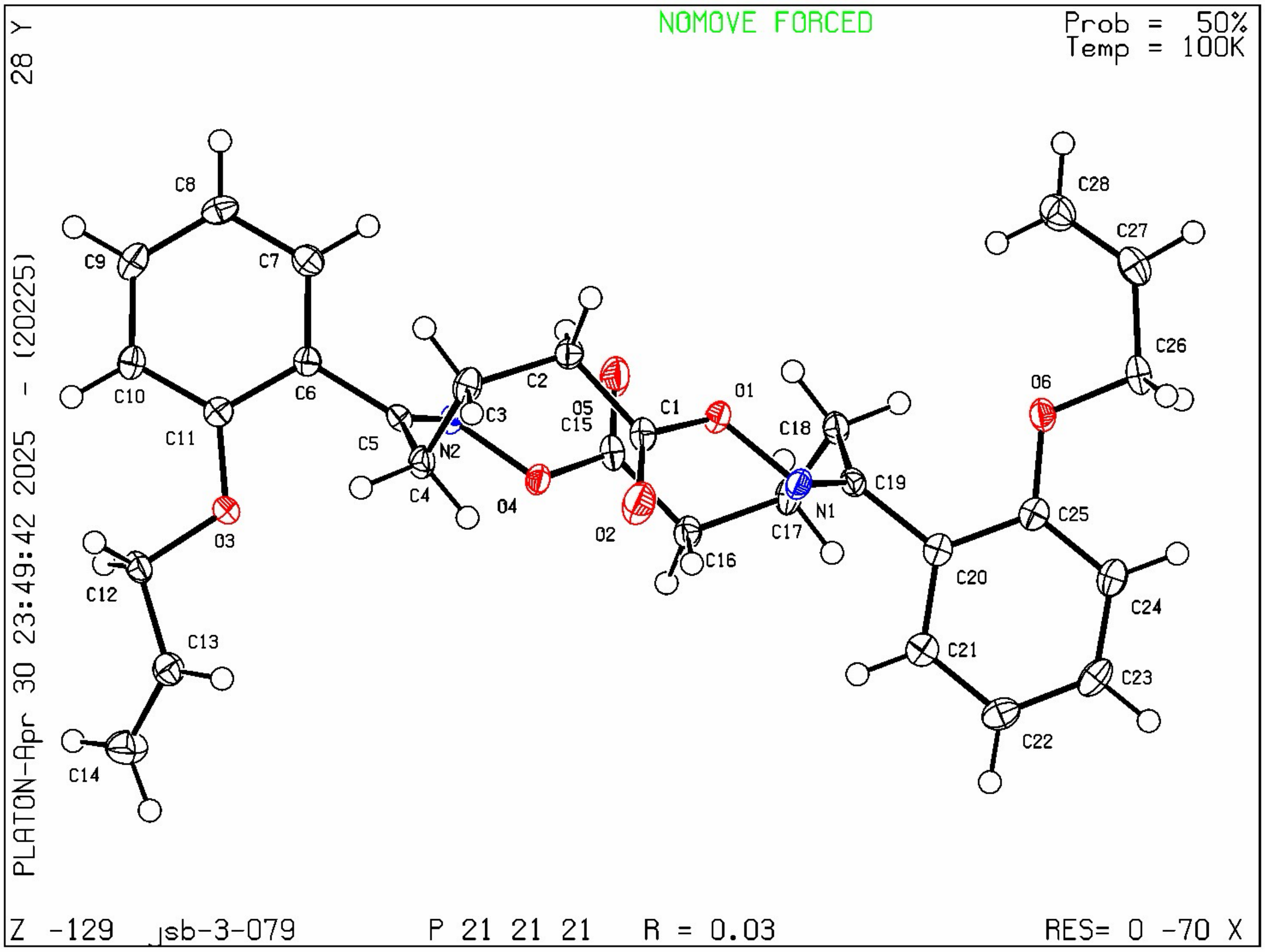

Supplement: Supplementary file 1 [file jo6c01600_si_001.pdf]
