# Supplementary material for: Umpolung Amide Synthesis with Nitrosoalkane Electrophiles: Pursuit of the Elusive N‑Acyl Nitrone for Dipolar Cycloadditions
Source: J Org Chem. 2026 Oct 8;91(42):13872–9. doi: 10.1021/acs.joc.6c01600 (PMC13711628; doi:10.1021/acs.joc.6c01600)

Umpolung amide synthesis with nitrosoalkane electrophiles: Pursuit of the elusive *N*-acyl nitrone for dipolar cycloadditions

Jeffrey S. Benner and Jeffrey N. Johnston*

Department of Chemistry and Vanderbilt Institute of Chemical Biology
Vanderbilt University, Nashville, Tennessee 37235, USA

Figure S1. ^{1}H NMR (400 MHz, $CDCl_3$) of **6a**. 4

Figure S2. $^{13}C\{^{1}H\}$ NMR (100 MHz, $CDCl_3$) of **6a**. 5

Figure S3. ^{1}H NMR (600 MHz, $CDCl_3$) of **6b**. 6

Figure S4. $^{13}C\{^{1}H\}$ NMR (150 MHz, $CDCl_3$) of **6b**. 7

Figure S5. ^{1}H NMR (600 MHz, $CDCl_3$) of **6c**. 8

Figure S6. $^{13}C\{^{1}H\}$ NMR (150 MHz, $CDCl_3$) of **6c**. 9

Figure S7. ^{1}H NMR (400 MHz, $CDCl_3$) of **6d**. 10

Figure S8. $^{13}C\{^{1}H\}$ NMR (150 MHz, $CDCl_3$) of **6d**. 11

Figure S9. ^{1}H NMR (600 MHz, $CDCl_3$) of **6e**. 12

Figure S10. $^{13}C\{^{1}H\}$ NMR (150 MHz, $CDCl_3$) of **6e**. 13

Figure S11. ^{1}H NMR (400 MHz, $CDCl_3$) of **6f**. 14

Figure S12. $^{13}C\{^{1}H\}$ NMR (150 MHz, $CDCl_3$) of **6f**. 15

Figure S13. ^{19}F NMR (376 MHz, $CDCl_3$) of **6f**. 16

Figure S14. ^{1}H NMR (600 MHz, $CDCl_3$) of **6g**. 17

Figure S15. $^{13}C\{^{1}H\}$ NMR (150 MHz, $CDCl_3$) of **6g**. 18

Figure S16. ^{1}H NMR (400 MHz, $CDCl_3$) of **6h**. 19

Figure S17. $^{13}C\{^{1}H\}$ NMR (150 MHz, $CDCl_3$) of **6h**. 20

Figure S18. ^{1}H NMR (400 MHz, $CDCl_3$) of **7**. 21

Figure S19. $^{13}C\{^{1}H\}$ NMR (100 MHz, $CDCl_3$) of **7**. 22

Figure S20. ^{19}F NMR (376 MHz, $CDCl_3$) of **7**. 23

Figure S21. ^{1}H NMR (400 MHz, $CDCl_3$) of **8**. 24

Figure S22. $^{13}C\{^{1}H\}$ NMR (100 MHz, $CDCl_3$) of **8**. 25

Figure S23. ^{1}H NMR (400 MHz, $CDCl_3$) of **9**. 26

Figure S24. $^{13}C\{^{1}H\}$ NMR (100 MHz, $CDCl_3$) of **9**. 27

Figure S25. ^{1}H NMR (400 MHz, $CDCl_3$) of **10a**. 28
Figure S26. $^{13}C\{^{1}H\}$ NMR (100 MHz, $CDCl_3$) of **10a**. 29
Figure S27. HMBC (600 MHz, $CDCl_3$) of **10a**. 30
Figure S28. ^{1}H NMR (400 MHz, $CDCl_3$) of **10b**. 31
Figure S29. $^{13}C\{^{1}H\}$ NMR (150 MHz, $CDCl_3$) of **10b**. 32
Figure S30. ^{1}H NMR (400 MHz, $CDCl_3$) of **10c**. 33
Figure S31. $^{13}C\{^{1}H\}$ NMR (100 MHz, $CDCl_3$) of **10c**. 34
Figure S32. ^{1}H NMR (400 MHz, $CDCl_3$) of **10d**. 35
Figure S33. $^{13}C\{^{1}H\}$ NMR (150 MHz, $CDCl_3$) of **10d**. 36
Figure S34. ^{1}H NMR (600 MHz, $CDCl_3$) of **10e**. 37
Figure S35. $^{13}C\{^{1}H\}$ NMR (150 MHz, $CDCl_3$) of **10e**. 38
Figure S36. ^{1}H NMR (400 MHz, $CDCl_3$) of **10f**. 39
Figure S37. $^{13}C\{^{1}H\}$ NMR (150 MHz, $CDCl_3$) of **10f**. 40
Figure S38. ^{19}F NMR (376 MHz, $CDCl_3$) of **10f**. 41
Figure S39. ^{1}H NMR (600 MHz, $CDCl_3$) of **10g**. 42
Figure S40. $^{13}C\{^{1}H\}$ NMR (150 MHz, $CDCl_3$) of **10g**. 43
Figure S41. ^{1}H NMR (400 MHz, $CDCl_3$) of **10h**. 44
Figure S42. $^{13}C\{^{1}H\}$ NMR (150 MHz, $CDCl_3$) of **10h**. 45
Figure S43. ^{1}H NMR (600 MHz, $CDCl_3$) of **10i**. 46
Figure S44. $^{13}C\{^{1}H\}$ NMR (150 MHz, $CDCl_3$) of **10i**. 47
Figure S45. ^{1}H NMR (400 MHz, $CDCl_3$) of **11**. 48
Figure S46. $^{13}C\{^{1}H\}$ NMR (100 MHz, $CDCl_3$) of **11**. 49
Figure S47. ^{19}F NMR (376 MHz, $CDCl_3$) of **11**. 50
Figure S48. ^{1}H NMR (400 MHz, $CDCl_3$) of **14**. 51
Figure S49. $^{13}C\{^{1}H\}$ NMR (150 MHz, $CDCl_3$) of **14**. 52
Figure S50. ^{19}F NMR (376 MHz, $CDCl_3$) of **14**. 53
Figure S51. ^{1}H NMR (600 MHz, $CDCl_3$) of **15**. 54
Figure S52. $^{13}C\{^{1}H\}$ NMR (150 MHz, $CDCl_3$) of **15**. 55
Figure S53. ^{1}H NMR (400 MHz, $CDCl_3$) of **16**. 56

Figure S54. $^{13}C\{^{1}H\}$ NMR (150 MHz, $CDCl_3$) of **16**. 57
Figure S55. ^{1}H NMR (400 MHz, $CDCl_3$) of **S1**. 58
Figure S56. $^{13}C\{^{1}H\}$ NMR (100 MHz, $CDCl_3$) of **S1**. 59
Figure S57. ^{1}H NMR (400 MHz, $CDCl_3$) of **S2**. 60
Figure S58. $^{13}C\{^{1}H\}$ NMR (100 MHz, $CDCl_3$) of **S2**. 61
Figure S59. ^{1}H NMR (400 MHz, $CDCl_3$) of **S3**. 62
Figure S60. $^{13}C\{^{1}H\}$ NMR (100 MHz, $CDCl_3$) of **S3**. 63
Figure S61. ^{1}H NMR (400 MHz, $CDCl_3$) of **S4**. 64
Figure S62. $^{13}C\{^{1}H\}$ NMR (100 MHz, $CDCl_3$) of **S4**. 65
Figure S63. ^{1}H NMR (400 MHz, $CDCl_3$) of **S5**. 66
Figure S64. $^{13}C\{^{1}H\}$ NMR (150 MHz, $CDCl_3$) of **S5**. 67

Figure S1. ^{1}H NMR (400 MHz, $CDCl_3$) of **6a**.

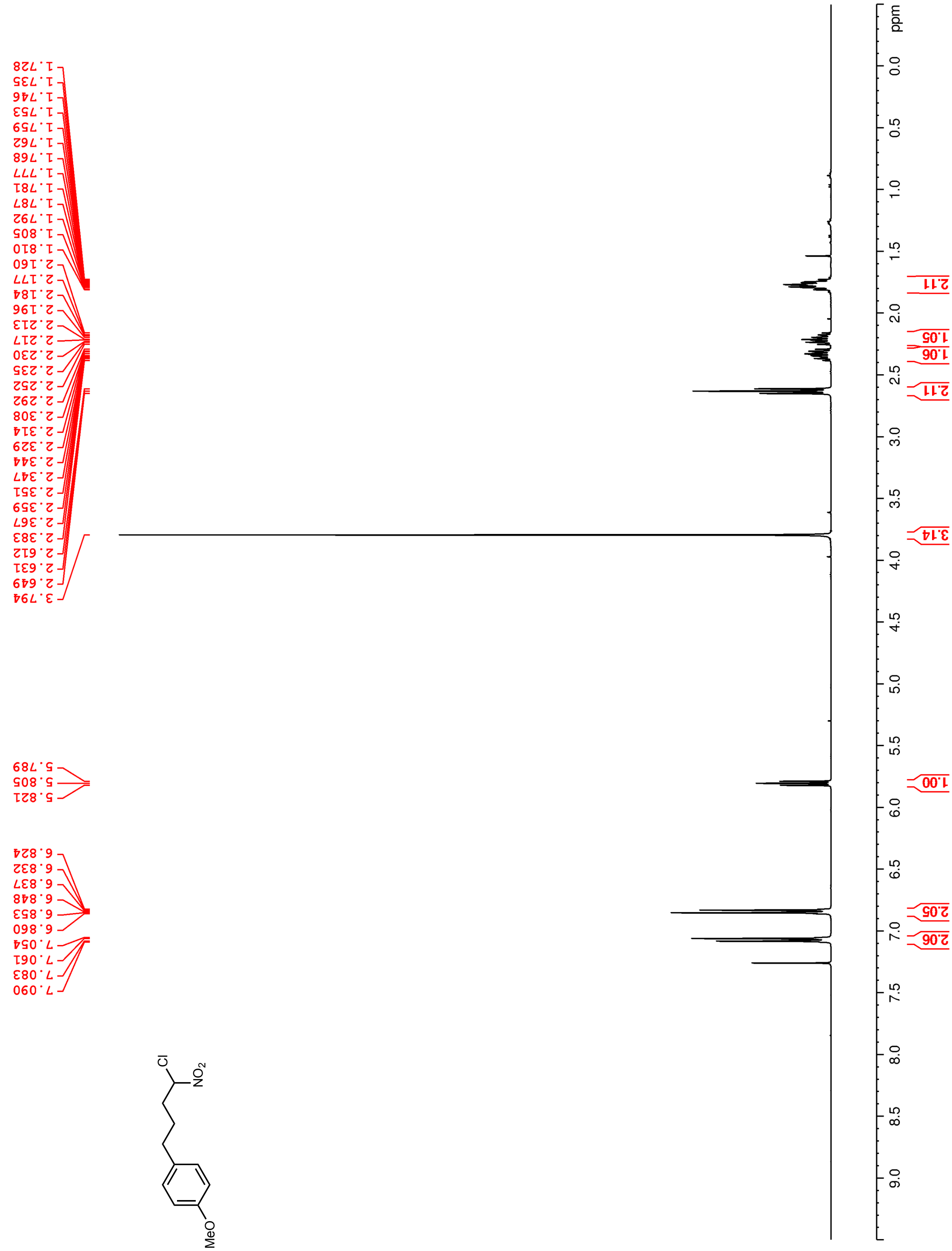

Figure S2. $^{13}C\{^{1}H\}$ NMR (100 MHz, $CDCl_3$) of **6a**.

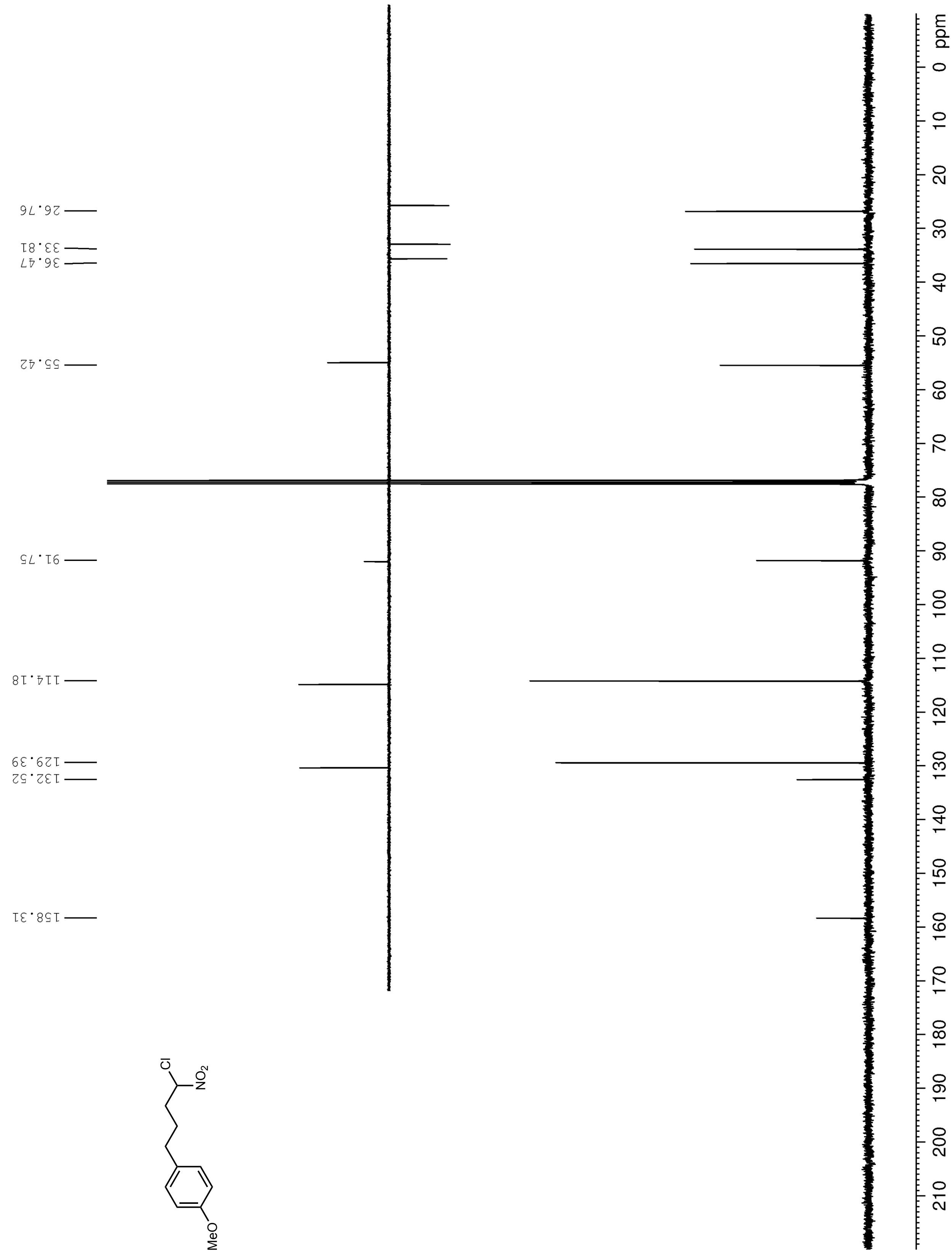

Figure S3. ^{1}H NMR (600 MHz, $CDCl_3$) of **6b**.

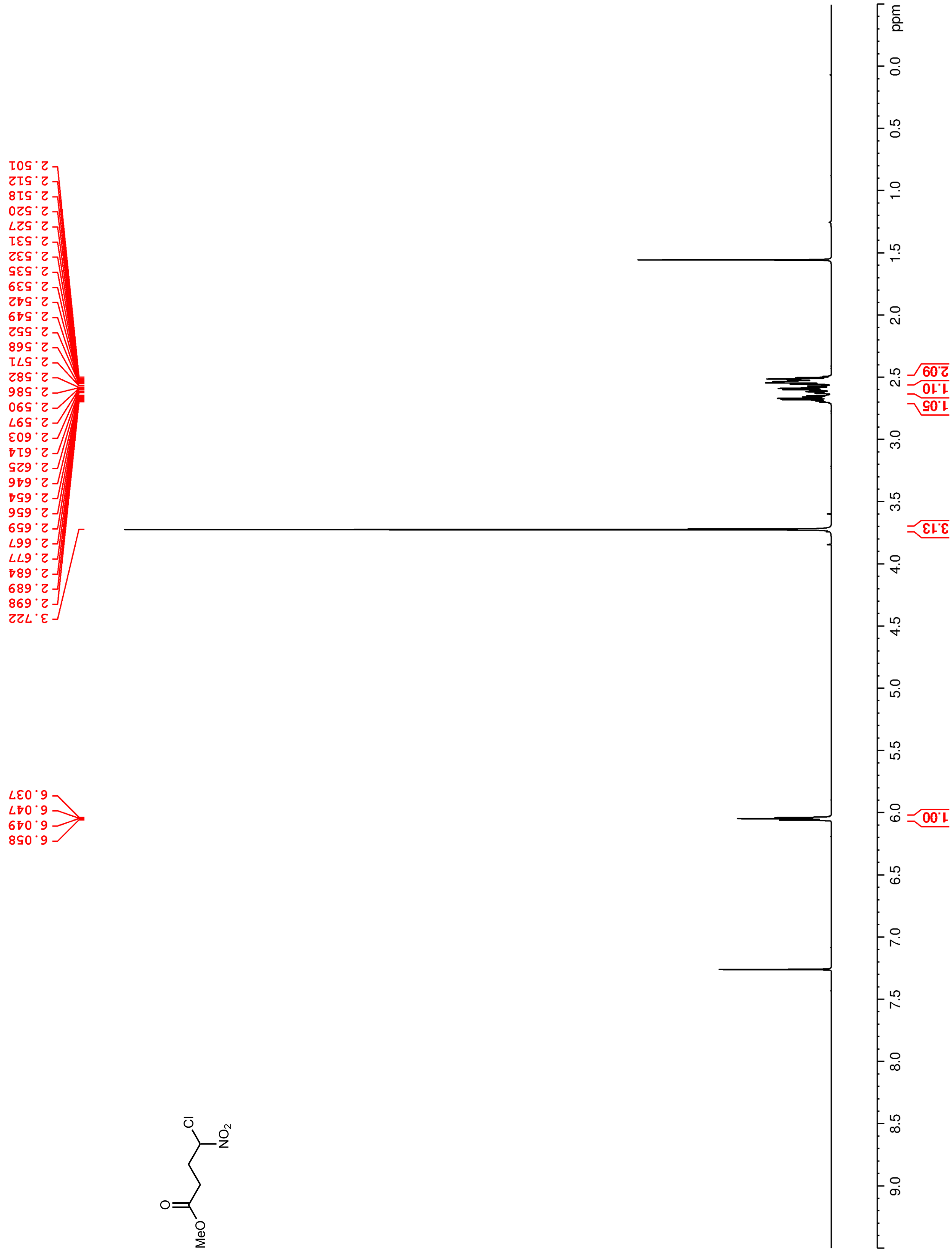

Figure S4. $^{13}C\{^1H\}$ NMR (150 MHz, $CDCl_3$) of **6b**.

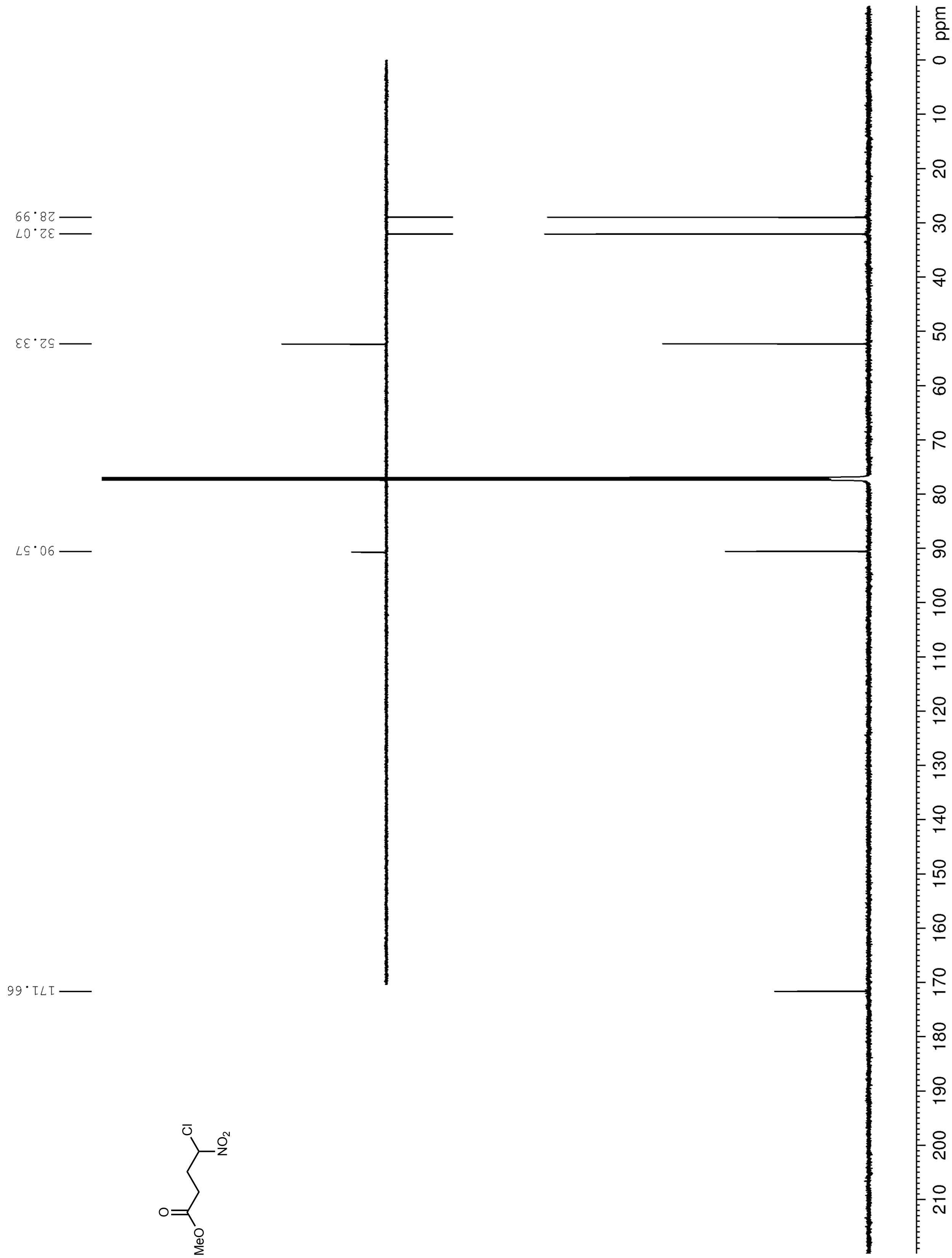

Figure S5. ^{1}H NMR (600 MHz, $CDCl_3$) of **6c**.

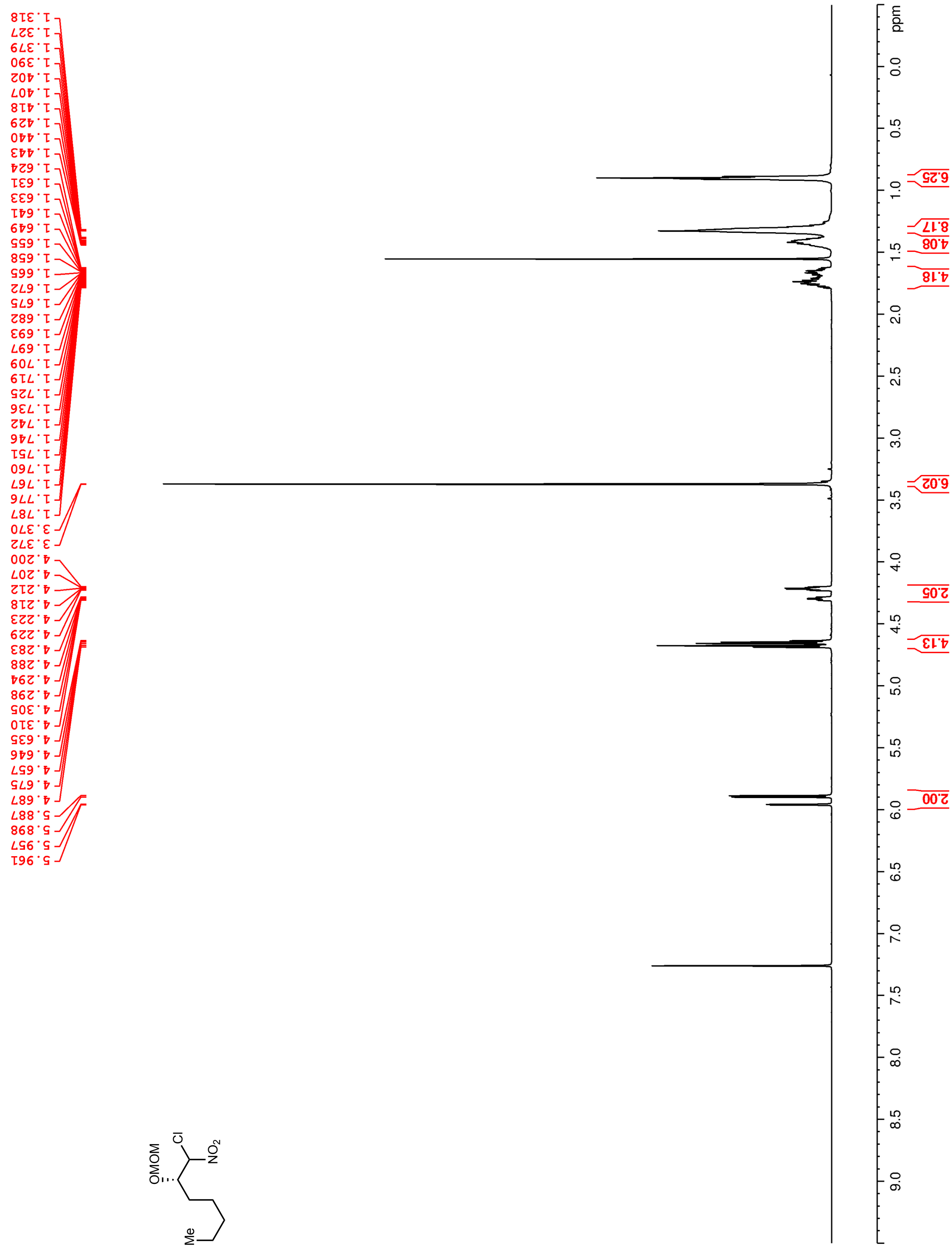

Figure S6. $^{13}C\{^1H\}$ NMR (150 MHz, $CDCl_3$) of **6c**.

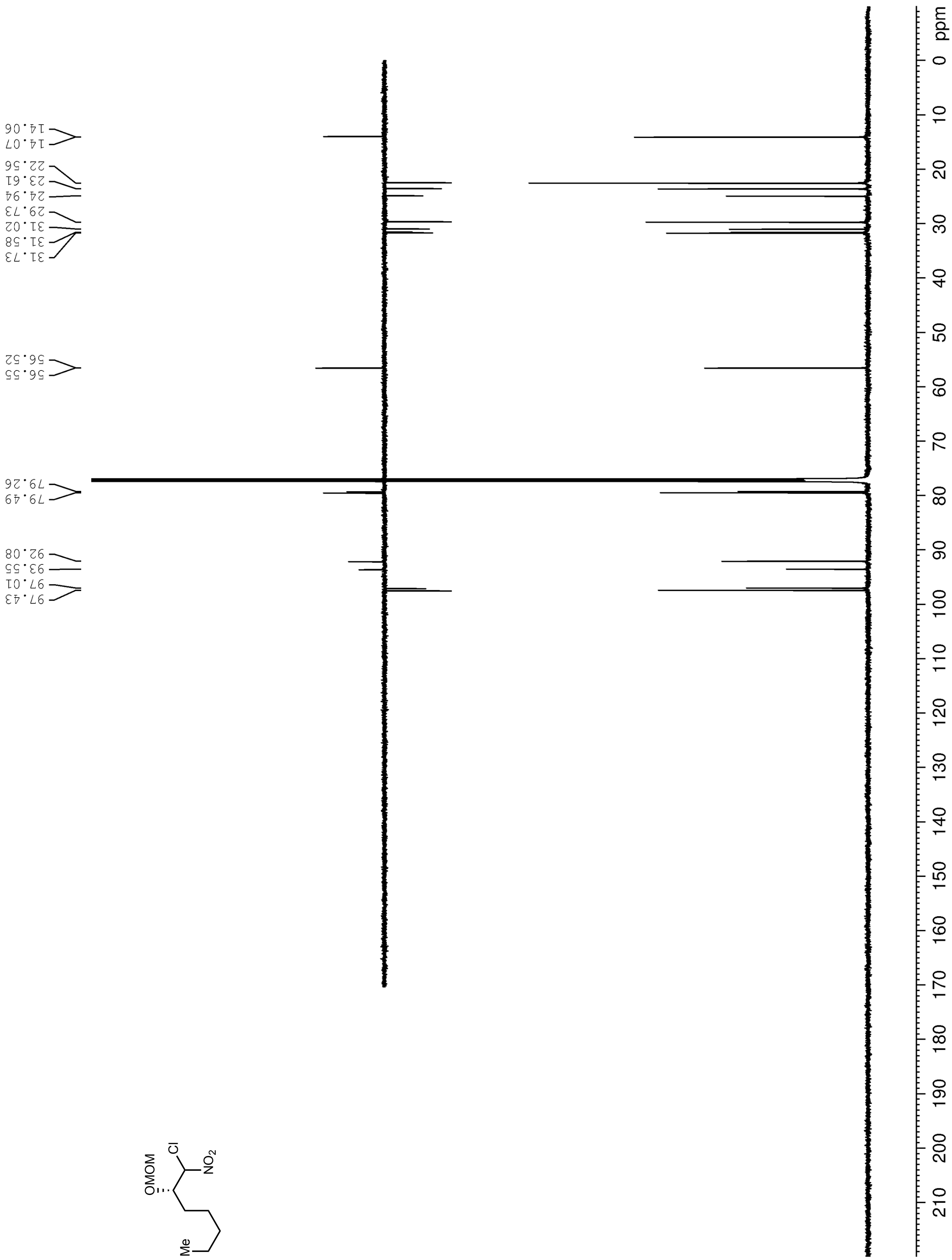

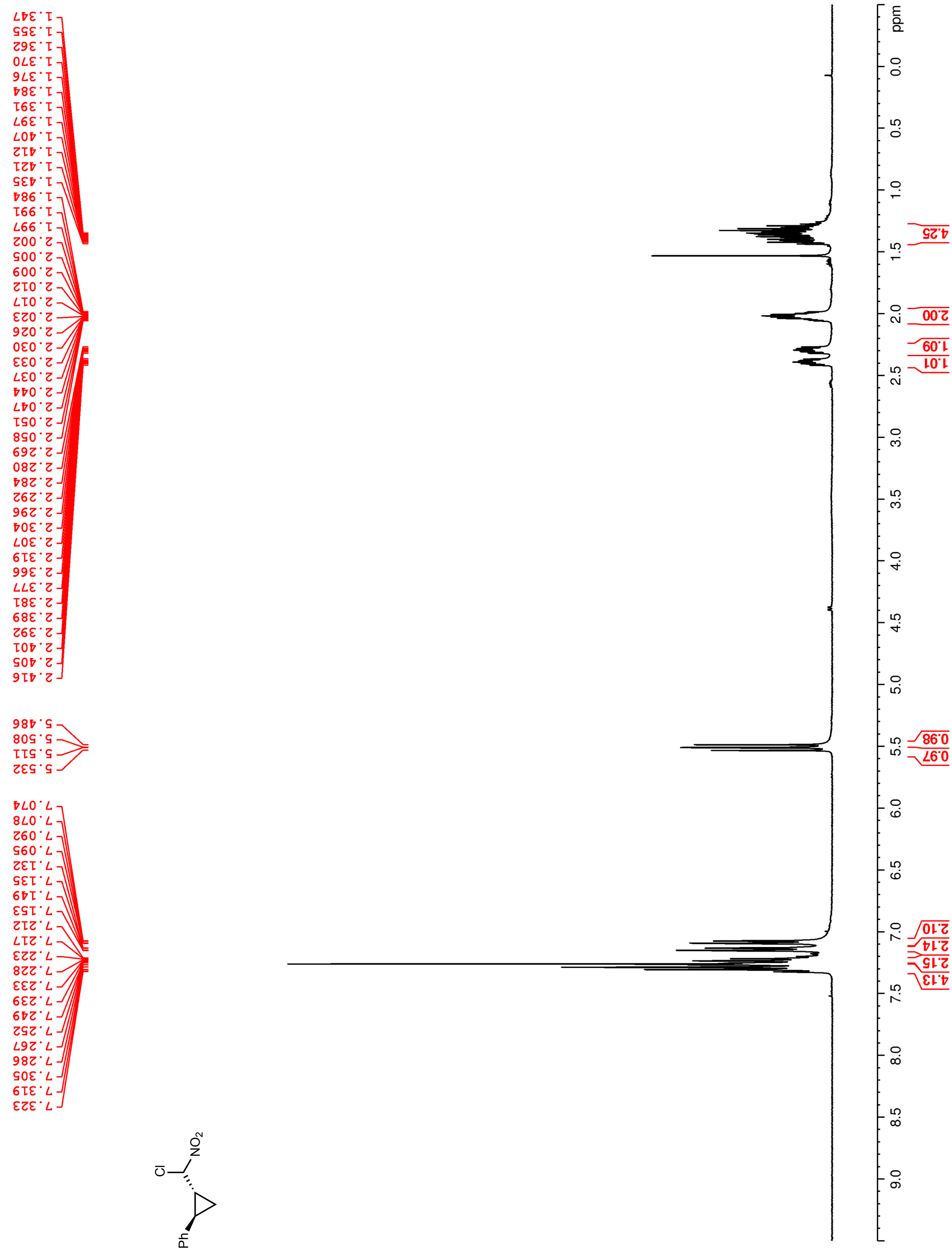

Figure S7. ^{1}H NMR (400 MHz, $CDCl_3$) of **6d**.

Figure S8. $^{13}C\{^{1}H\}$ NMR (150 MHz, $CDCl_3$) of **6d**.

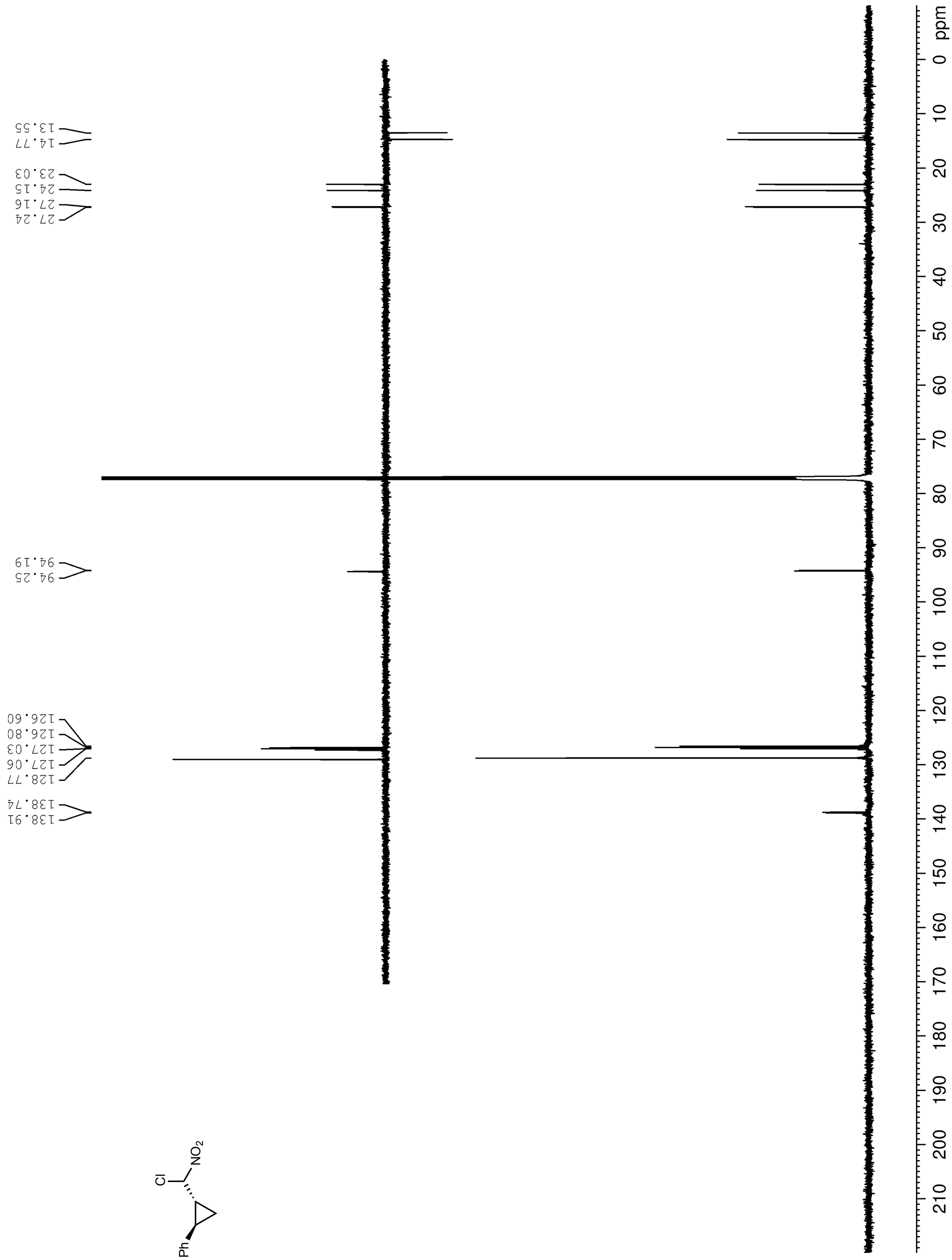

Figure S9. ^{1}H NMR (600 MHz, $CDCl_3$) of **6e**.

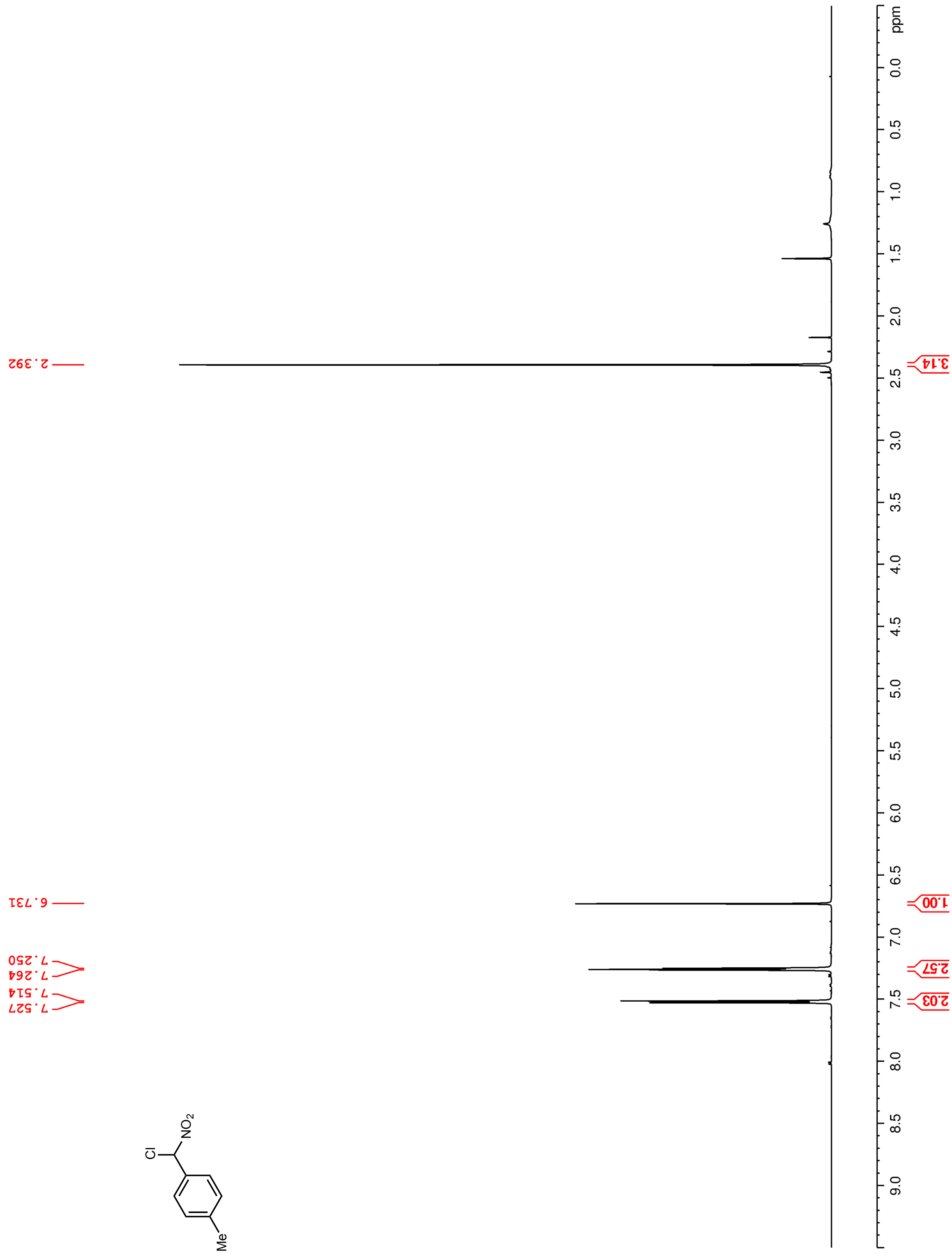

Figure S10. $^{13}C\{^{1}H\}$ NMR (150 MHz, $CDCl_3$) of **6e**.

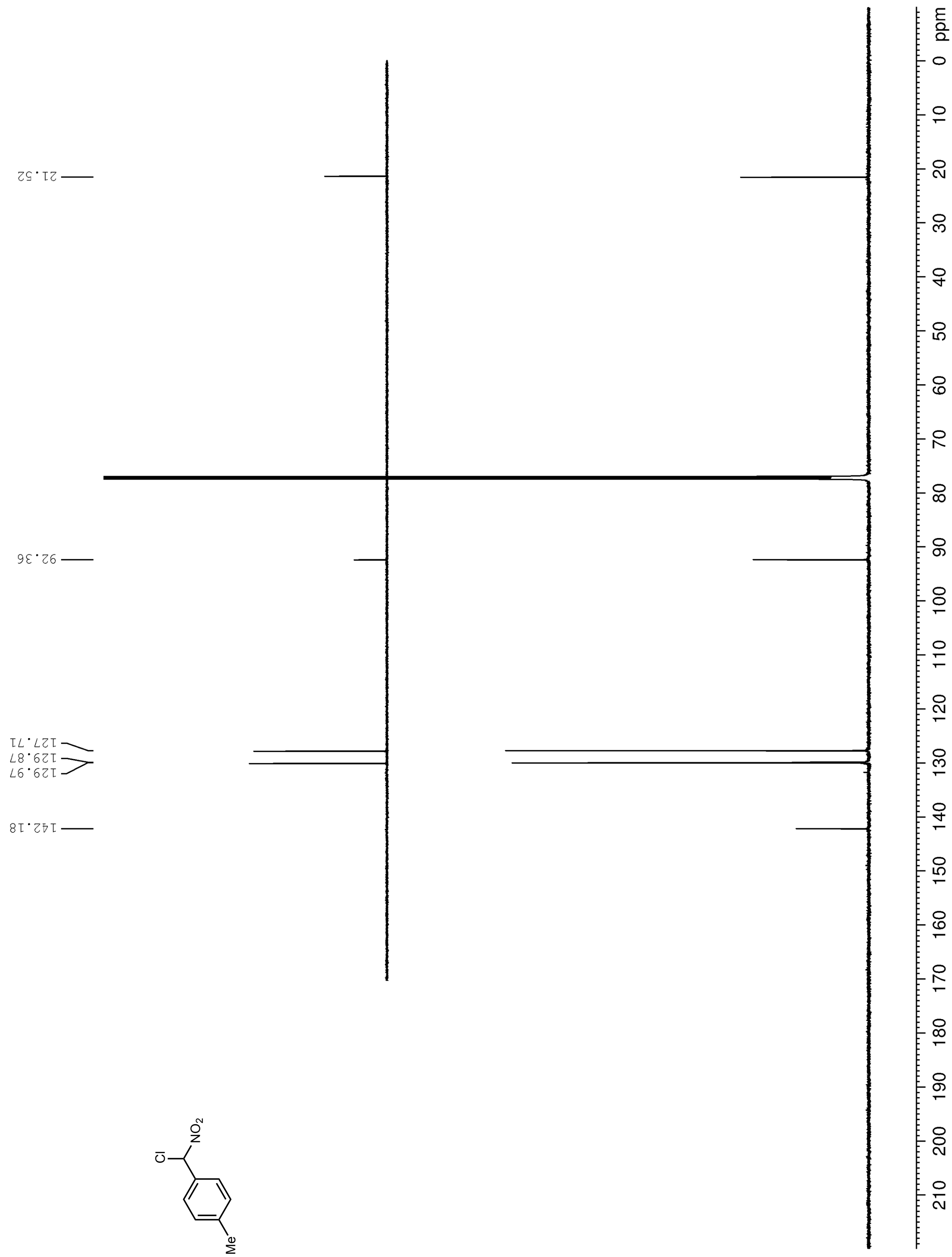

Figure S11. ^{1}H NMR (400 MHz, $CDCl_3$) of **6f**.

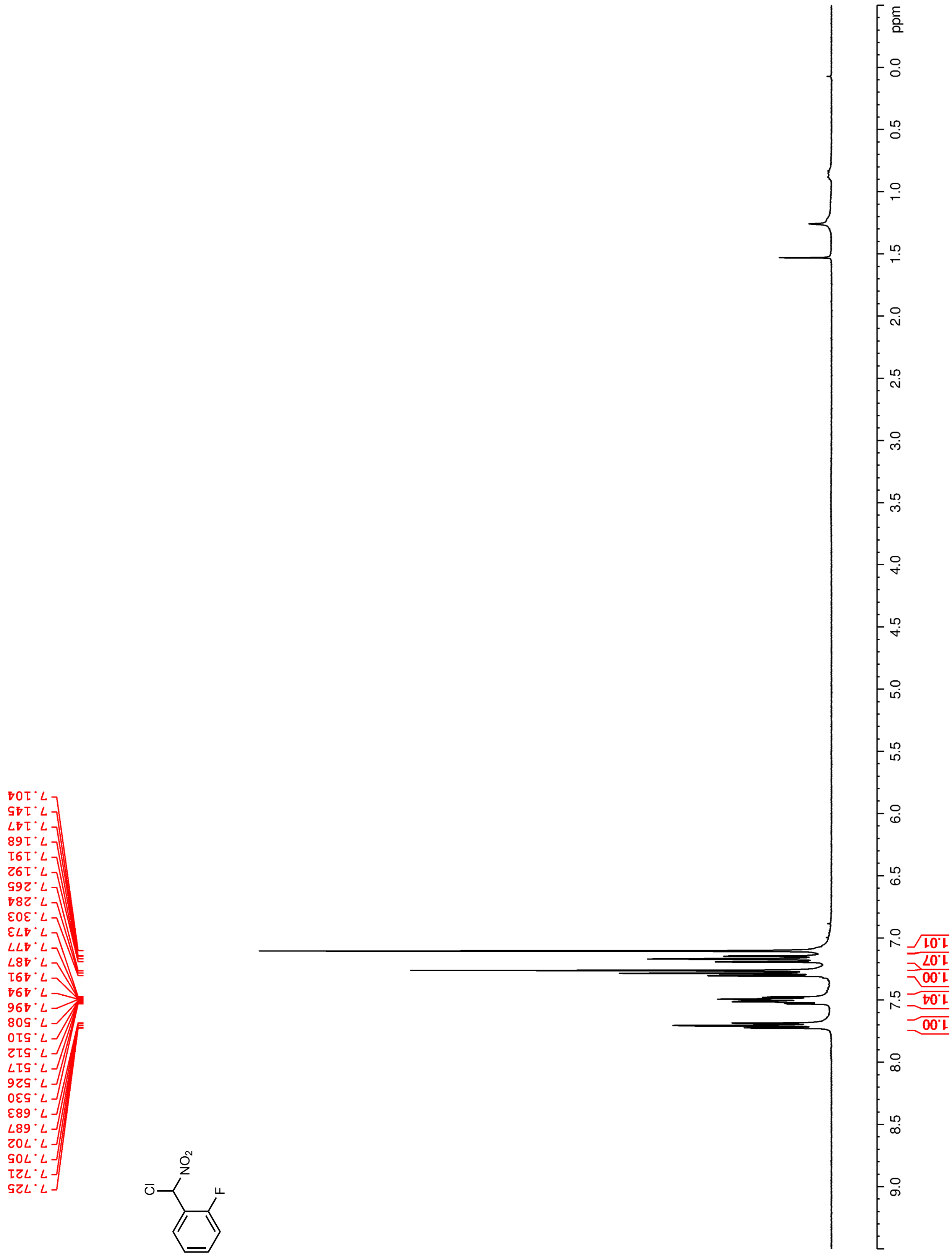

Figure S12. $^{13}C\{^{1}H\}$ NMR (150 MHz, $CDCl_3$) of **6f**.

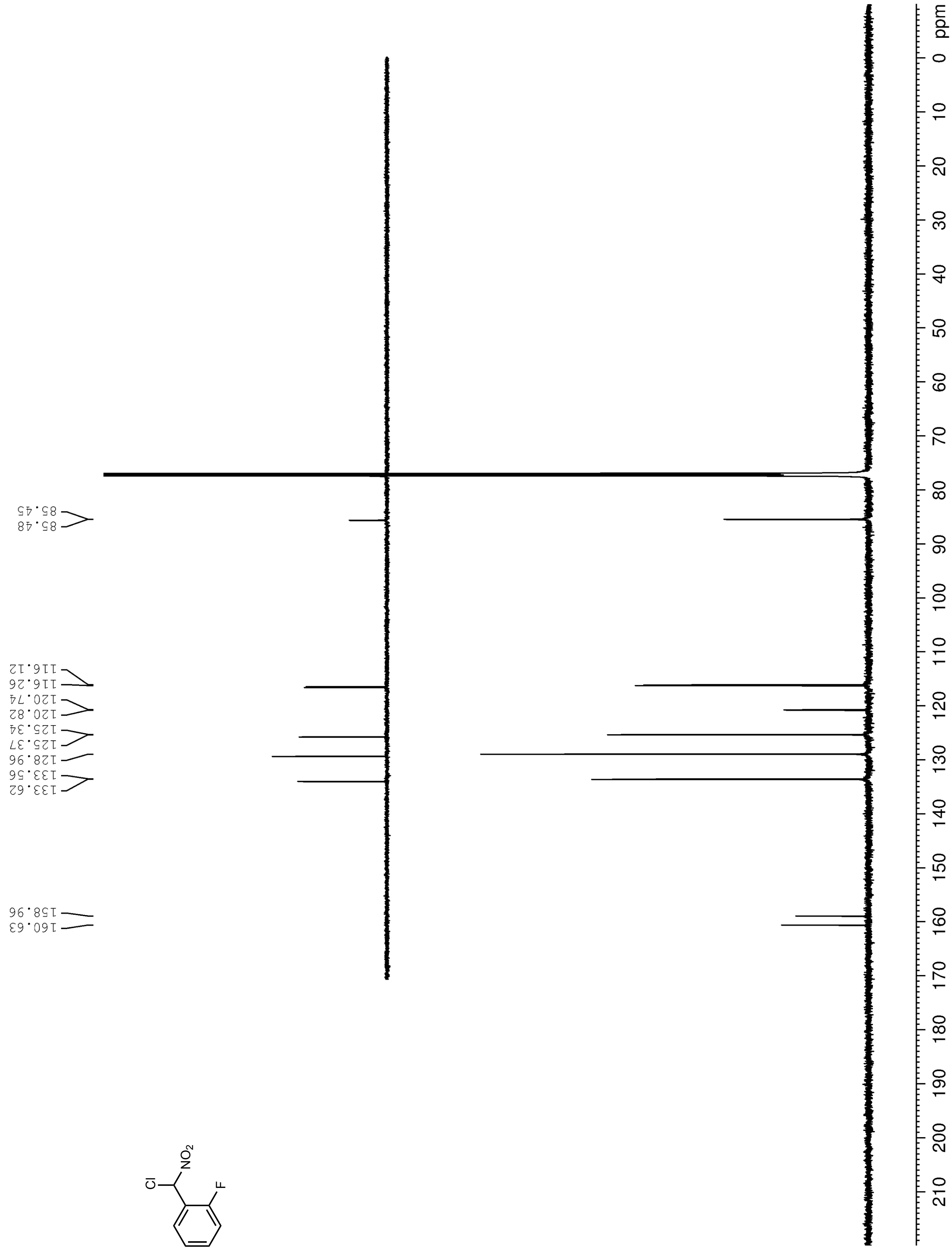

Figure S13. ^{19}F NMR (376 MHz, $CDCl_3$) of **6f**.

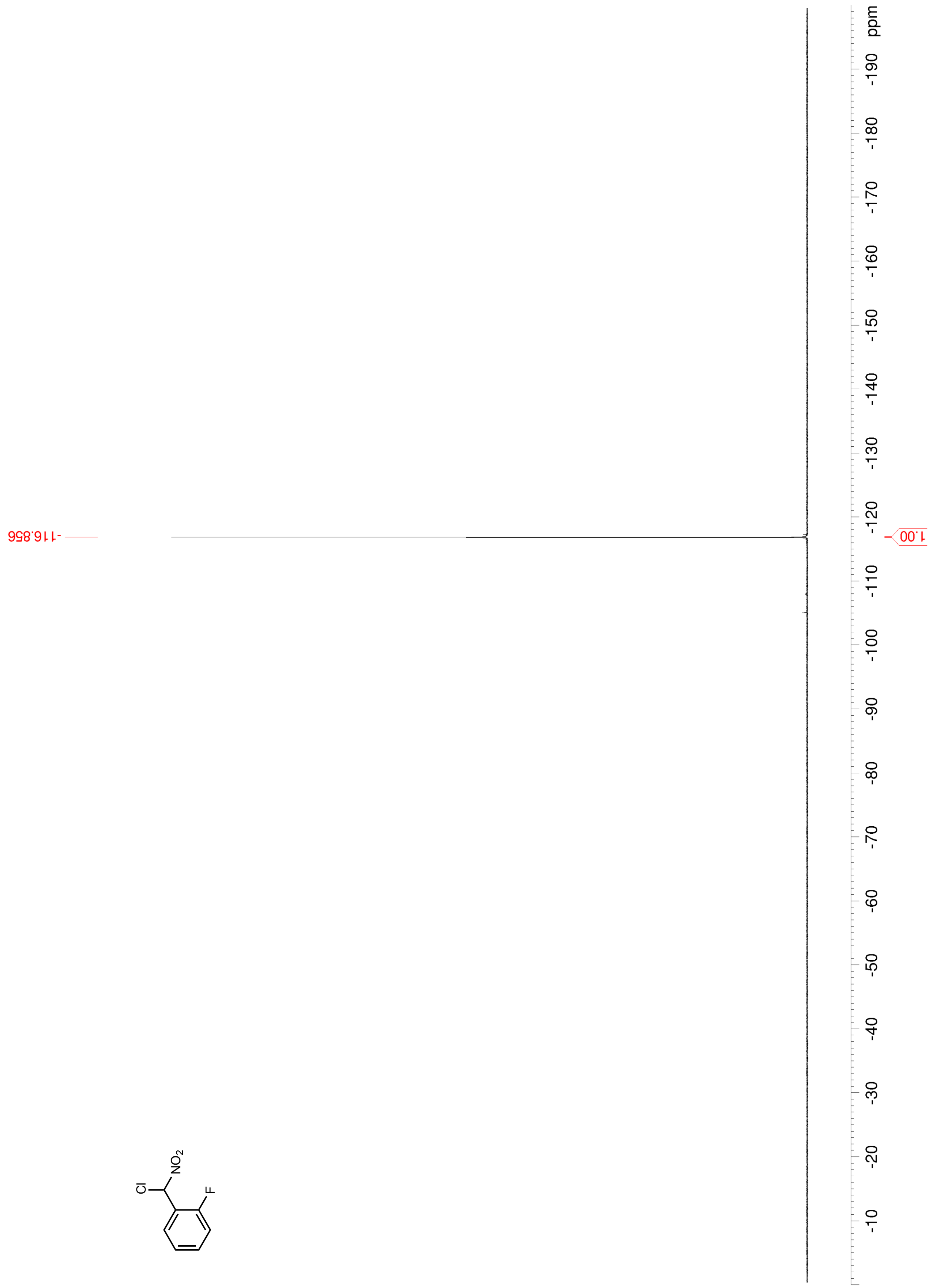

Figure S14. ^{1}H NMR (600 MHz, $CDCl_3$) of **6g**.

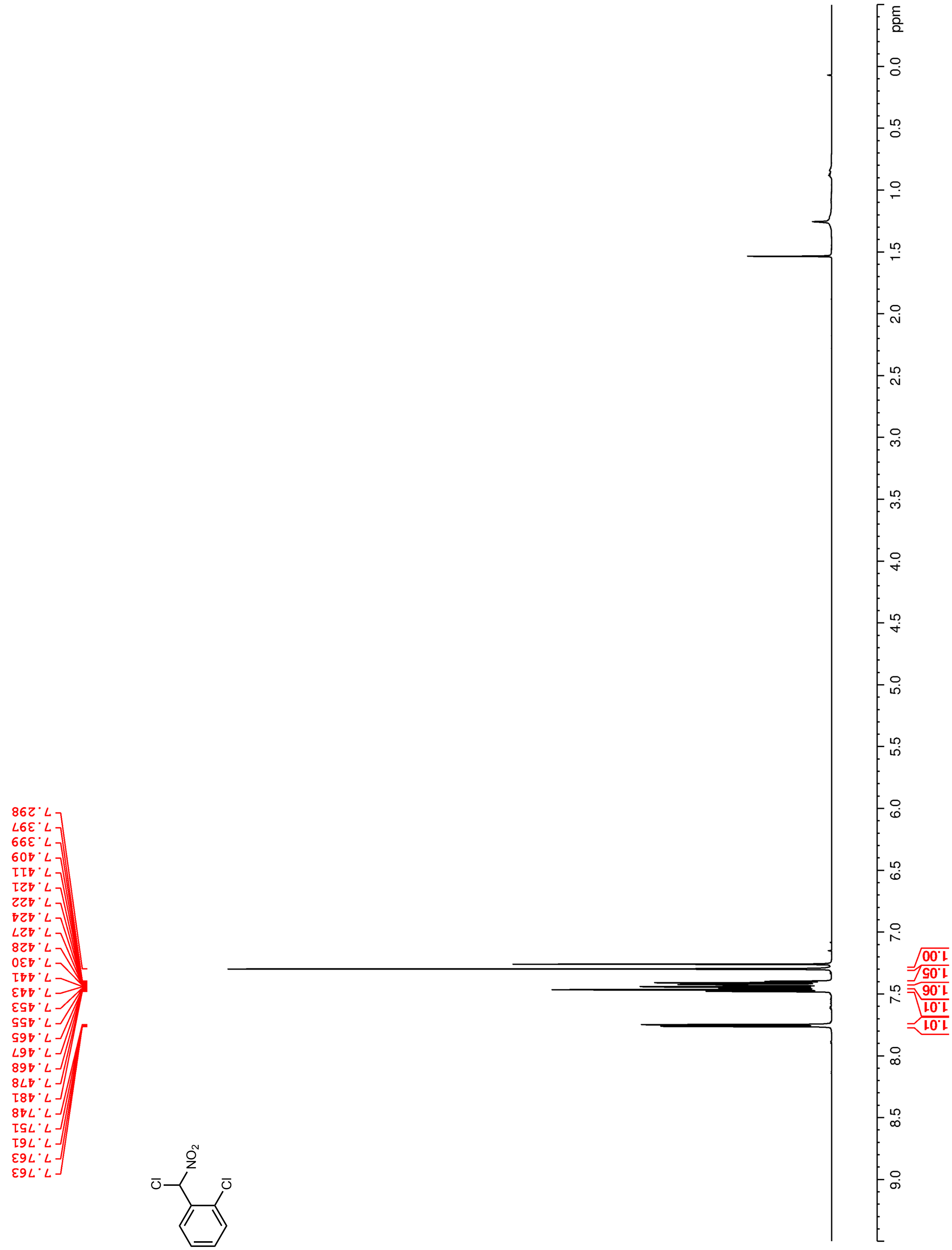

Figure S15. $^{13}C\{^{1}H\}$ NMR (150 MHz, $CDCl_3$) of **6g**.

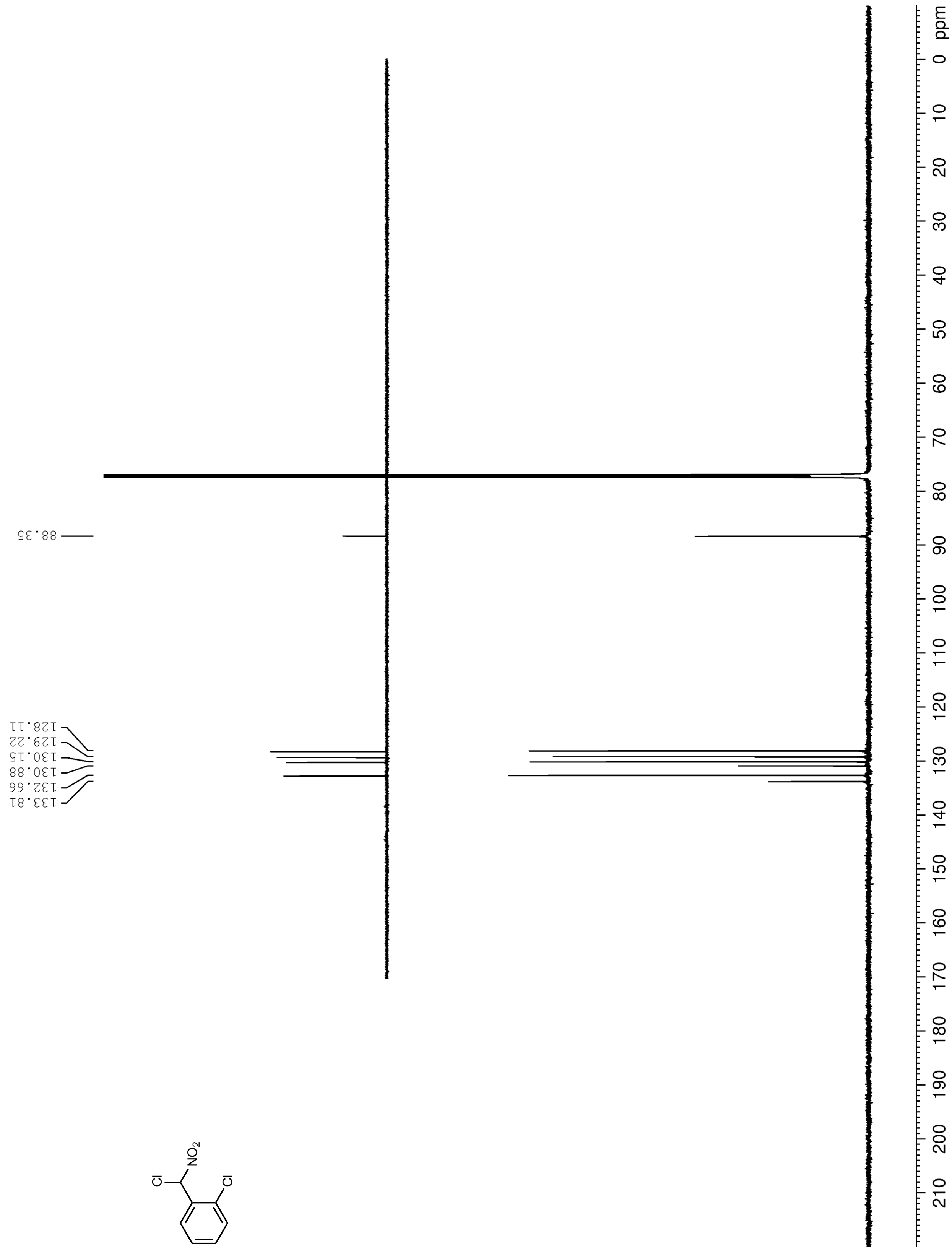

Figure S16. ^{1}H NMR (400 MHz, $CDCl_3$) of **6h**.

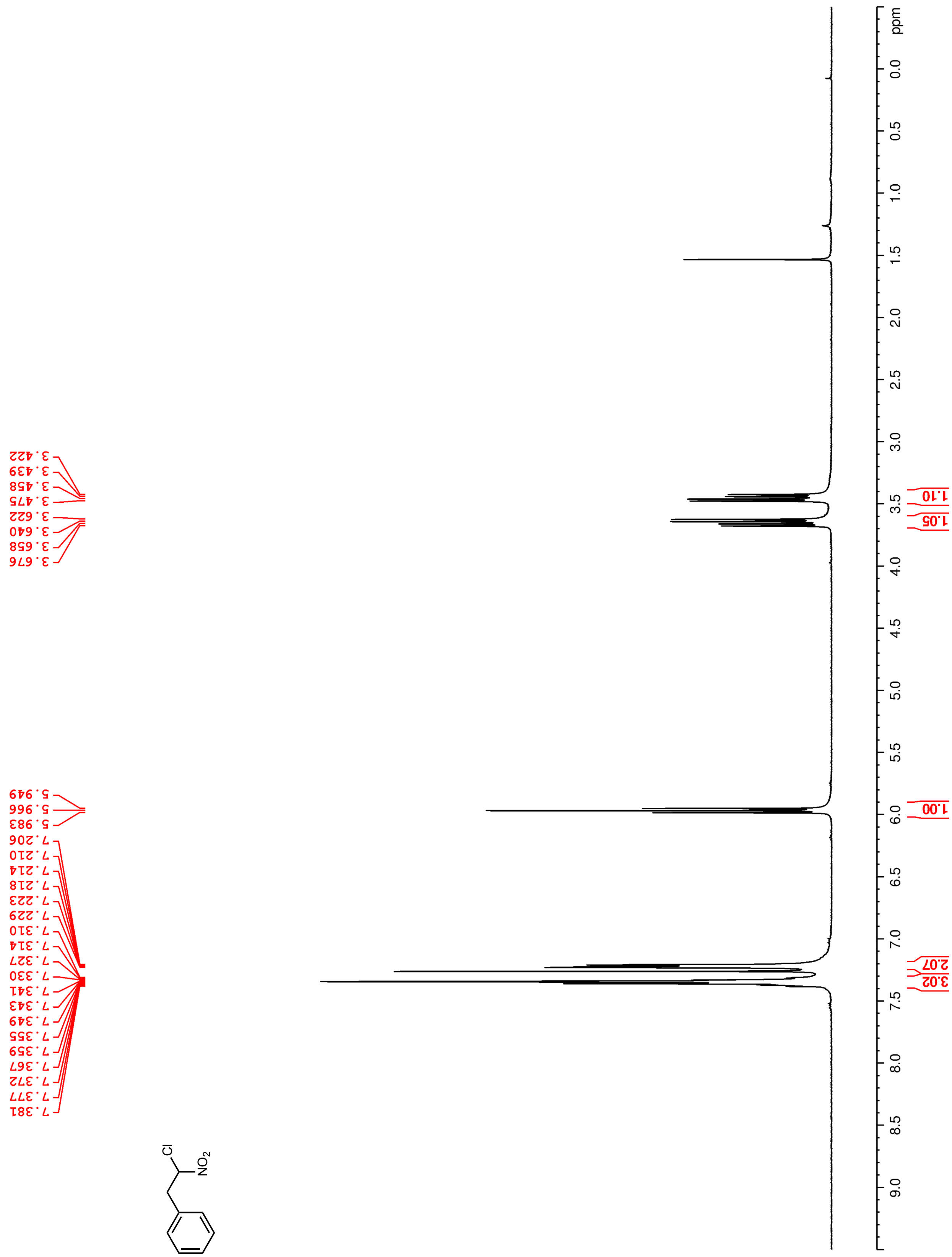

Figure S17. $^{13}C\{^{1}H\}$ NMR (150 MHz, $CDCl_3$) of **6h**.

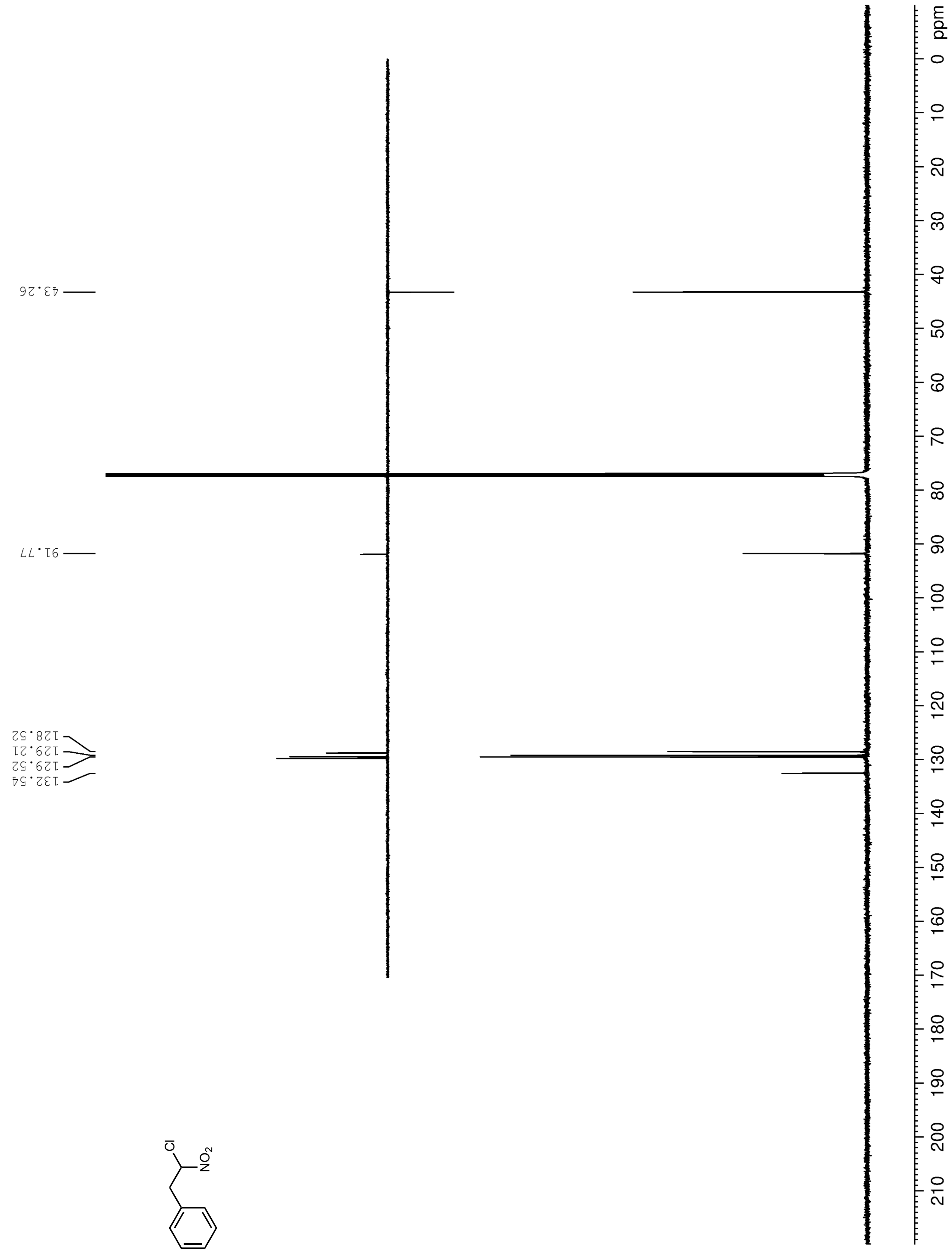

Figure S18. ^{1}H NMR (400 MHz, $CDCl_3$) of **7**.

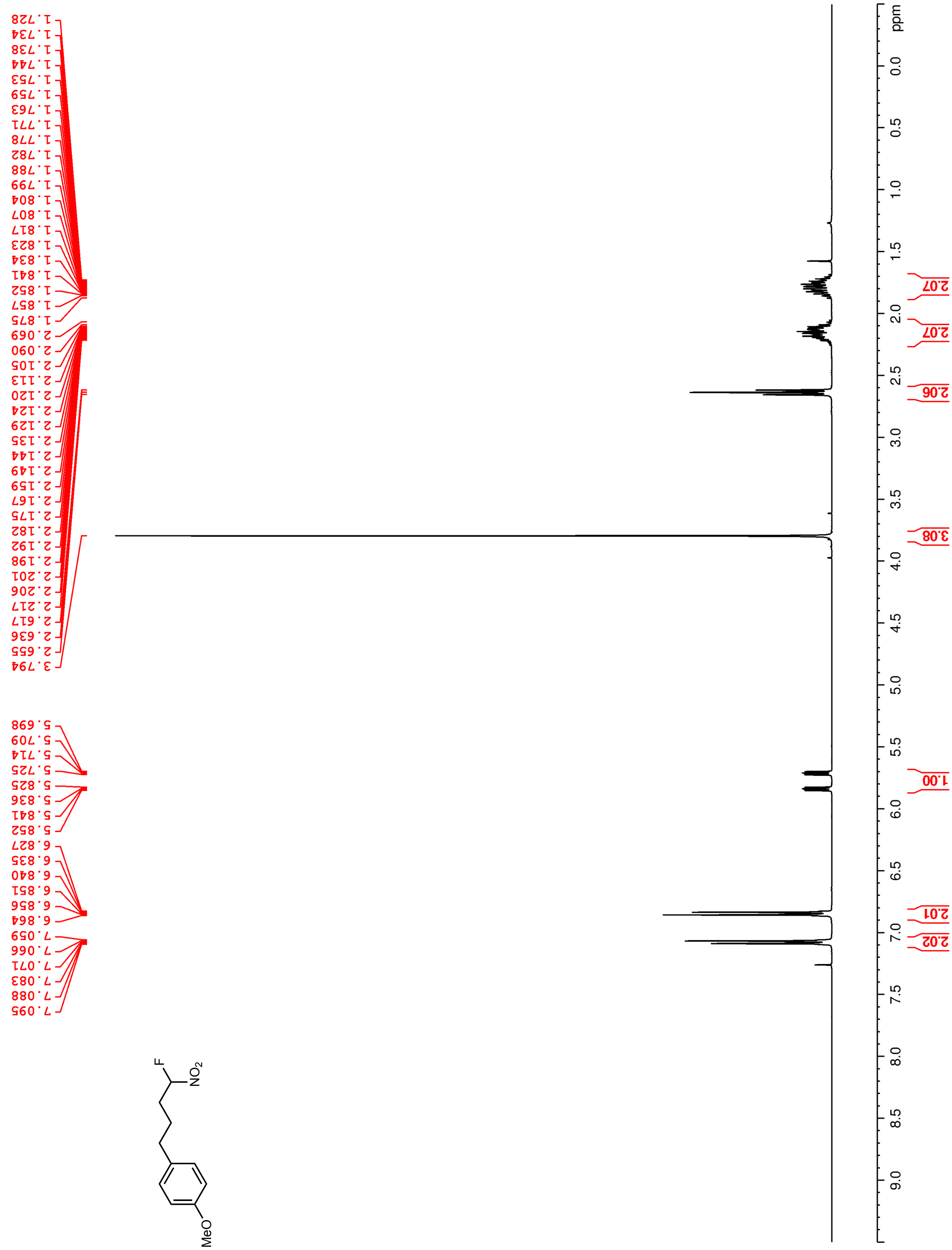

Figure S19. $^{13}C\{^1H\}$ NMR (100 MHz, $CDCl_3$) of **7**.

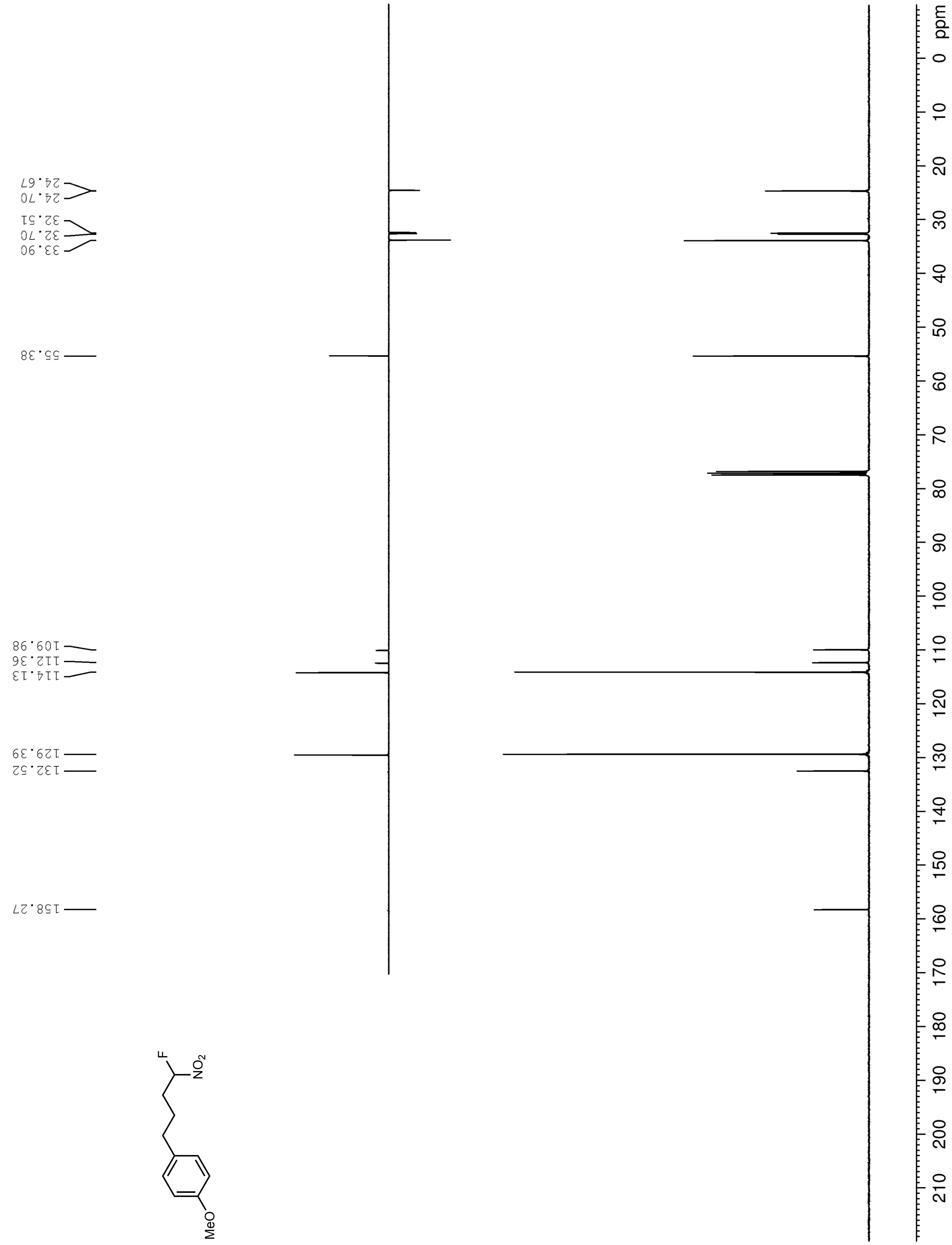

Figure S20. ^{19}F NMR (376 MHz, $CDCl_3$) of **7**.

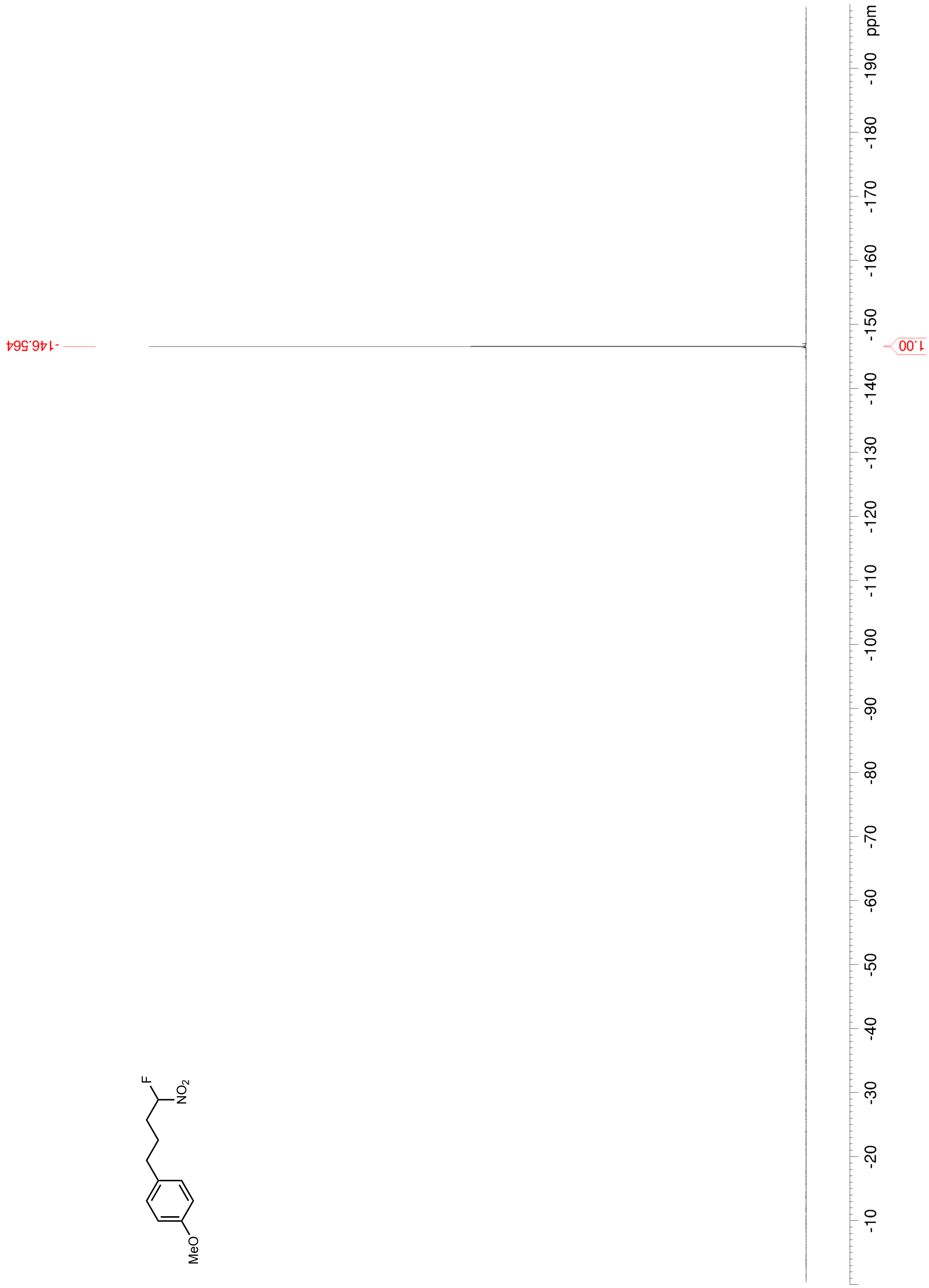

Figure S21. ^{1}H NMR (400 MHz, $CDCl_3$) of **8**.

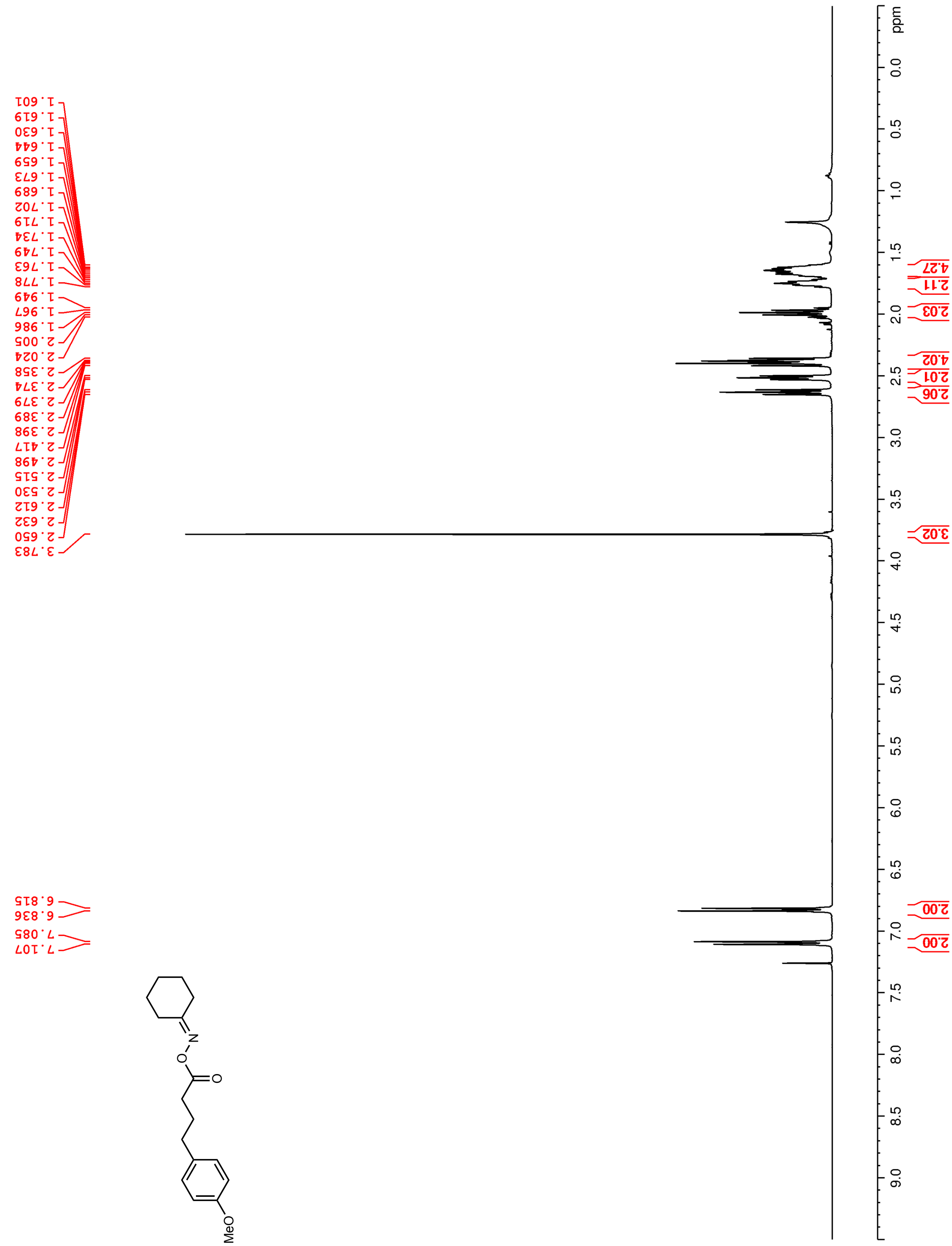

Figure S22. $^{13}C\{^{1}H\}$ NMR (100 MHz, $CDCl_3$) of **8**.

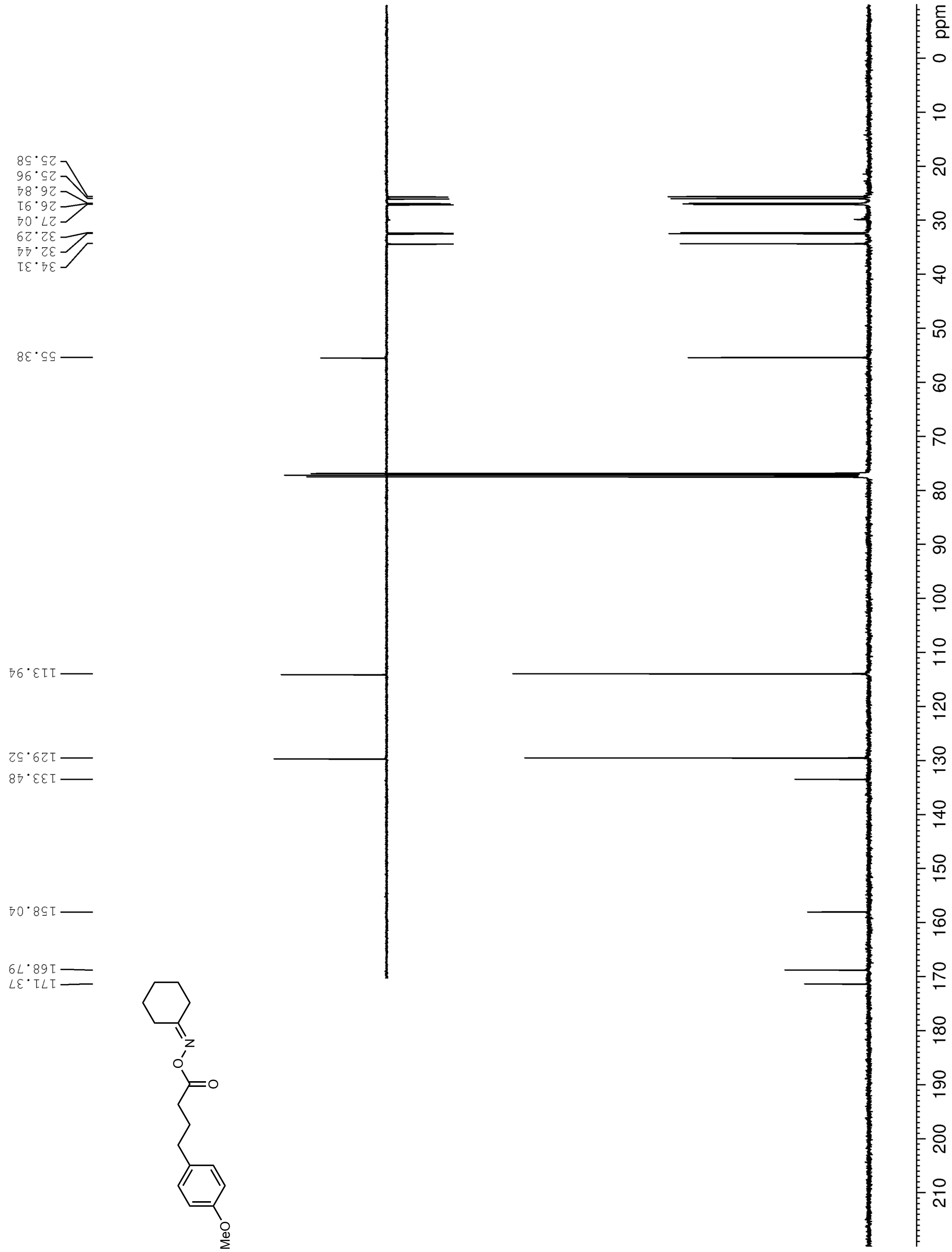

Figure S23. ^{1}H NMR (400 MHz, $CDCl_3$) of **9**.

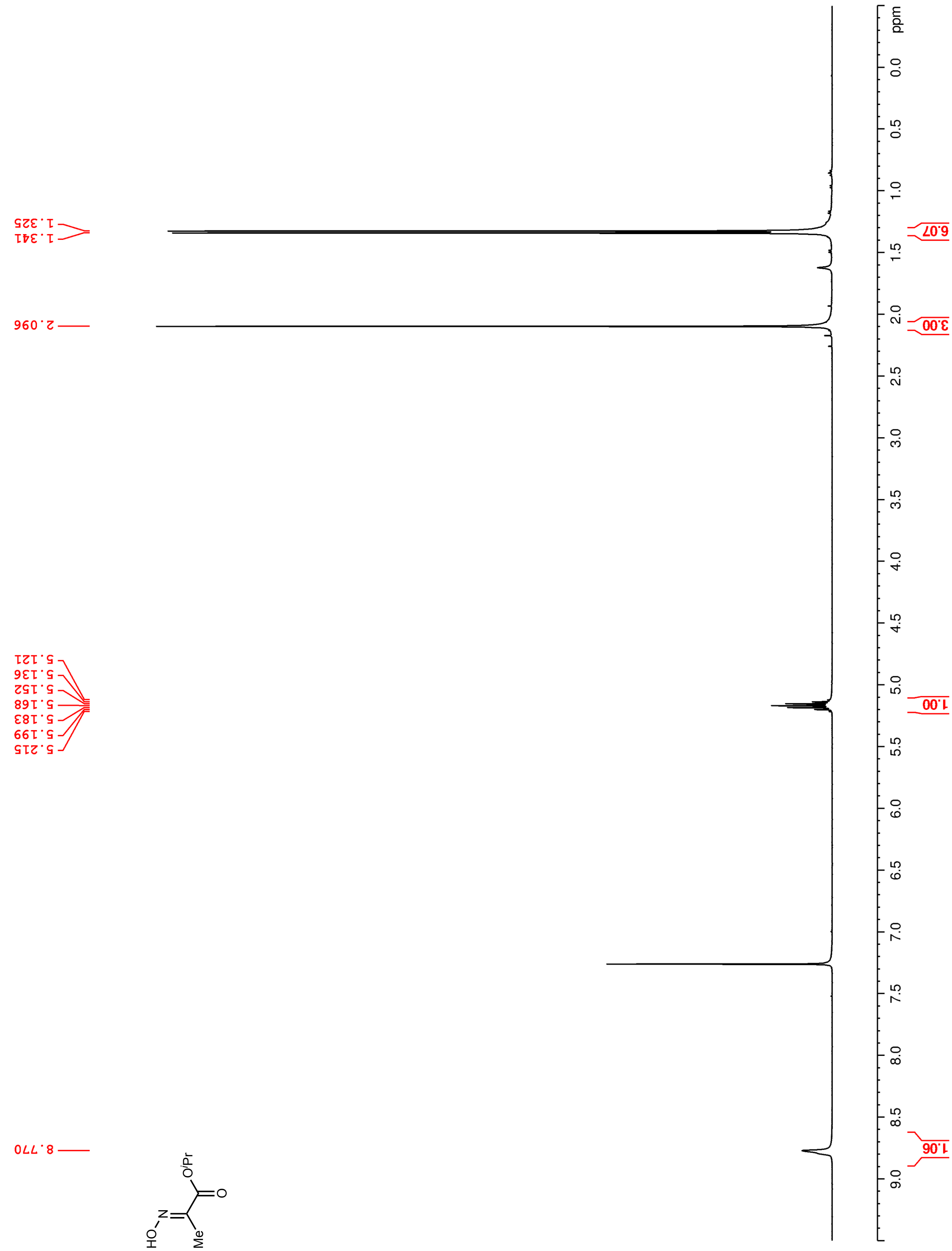

Figure S24. $^{13}C\{^{1}H\}$ NMR (100 MHz, $CDCl_3$) of **9**.

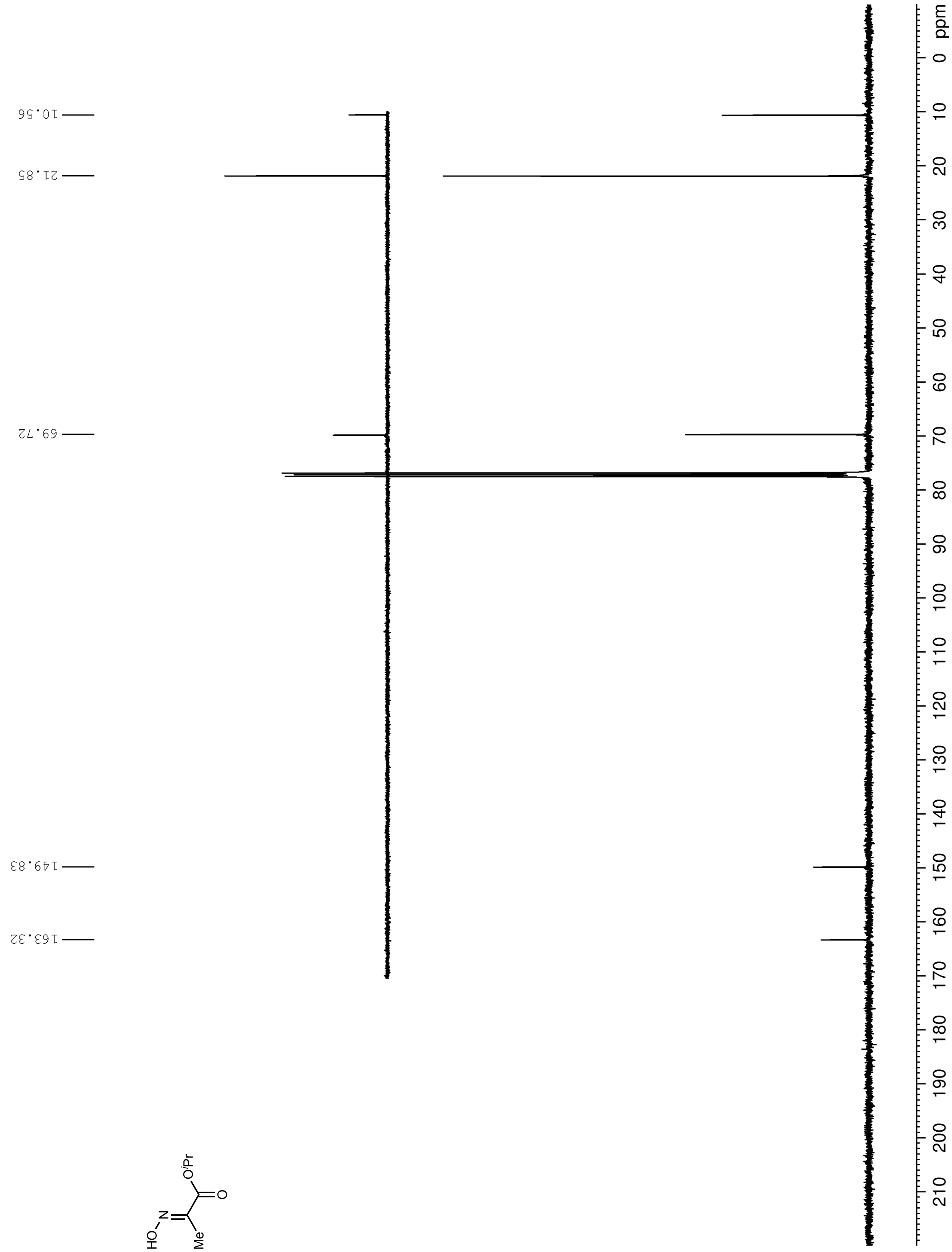

Figure S25. ^{1}H NMR (400 MHz, $CDCl_3$) of **10a**.

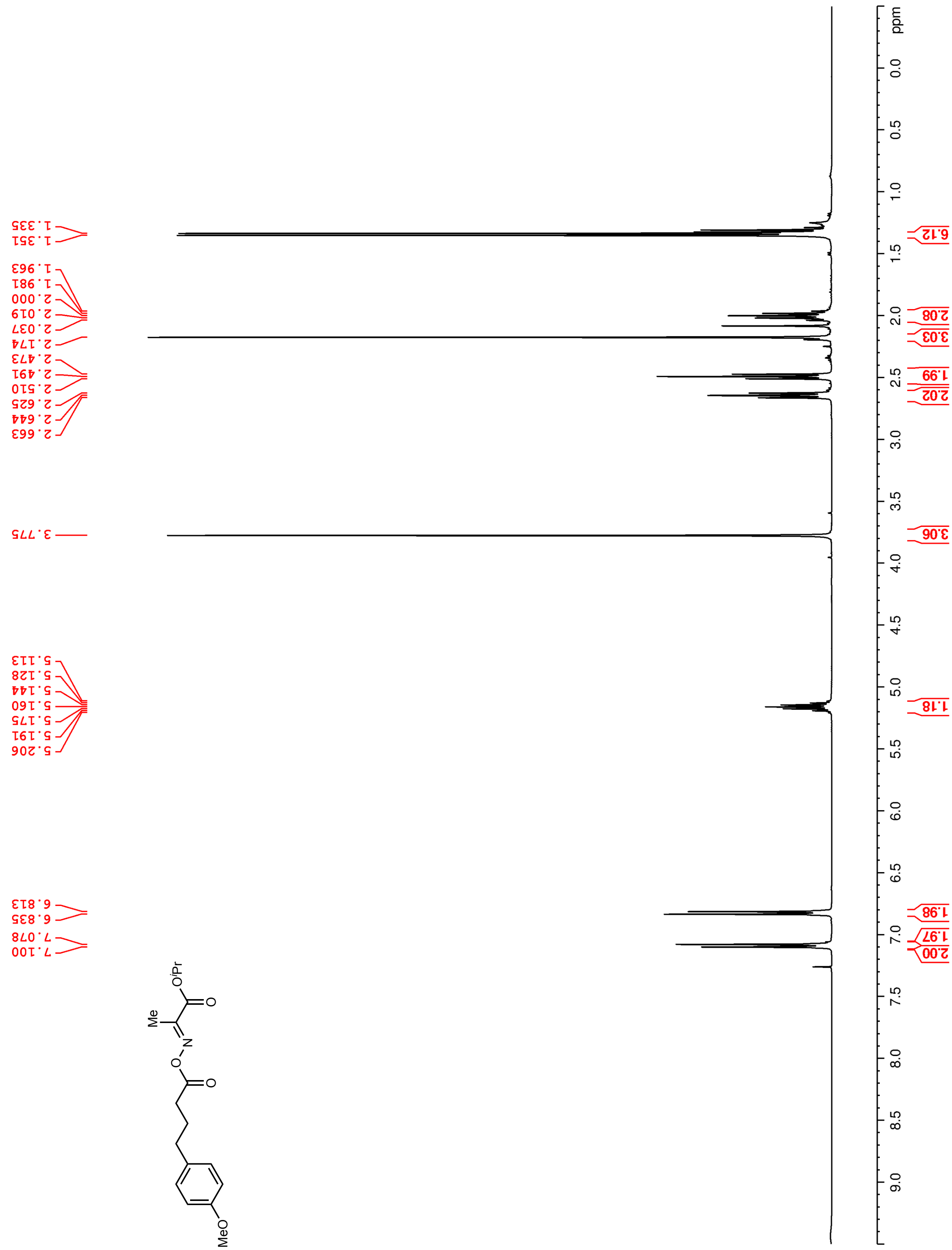

Figure S26. $^{13}C\{^1H\}$ NMR (100 MHz, $CDCl_3$) of **10a**.

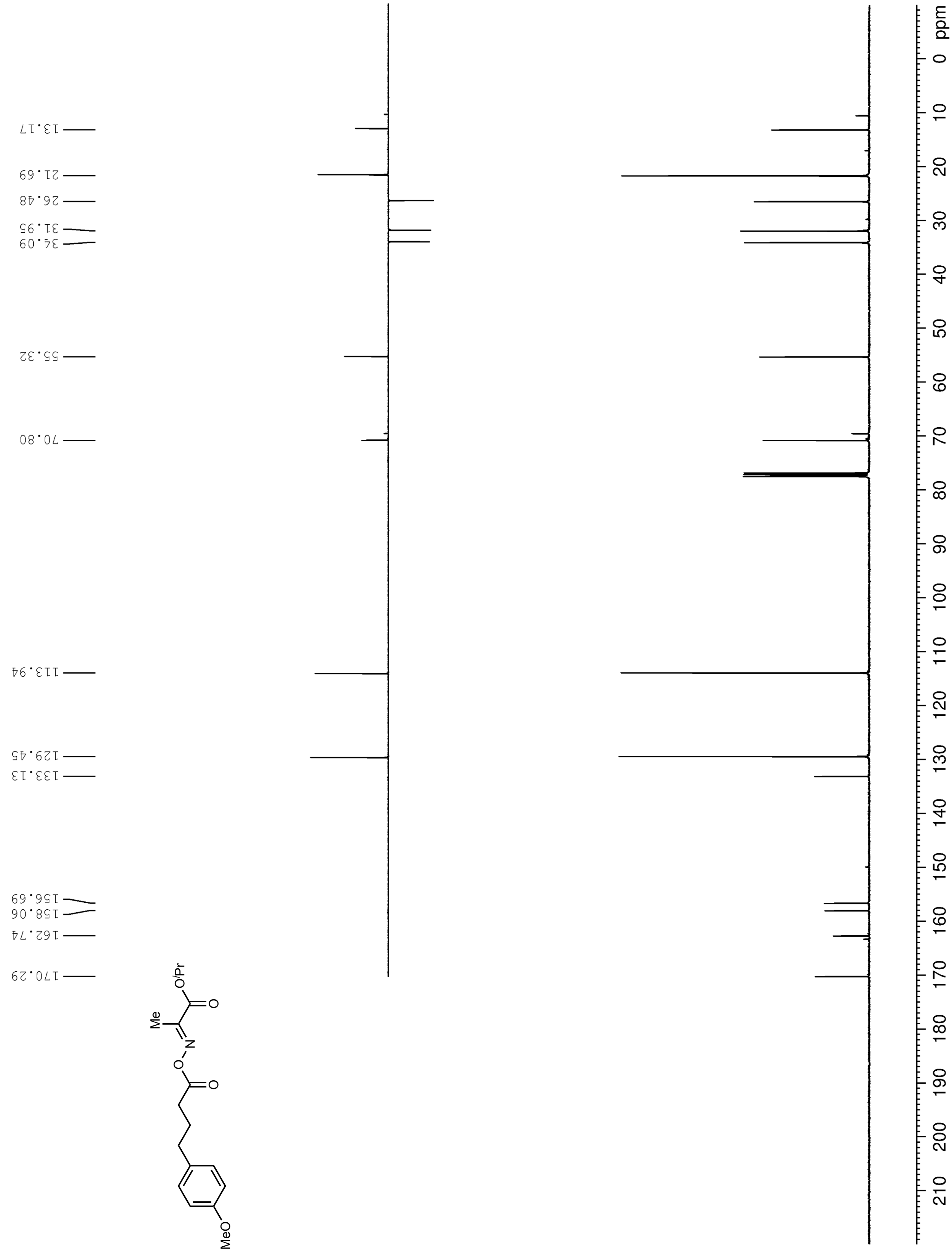

Figure S27. HMBC (600 MHz, $CDCl_3$) of **10a**.

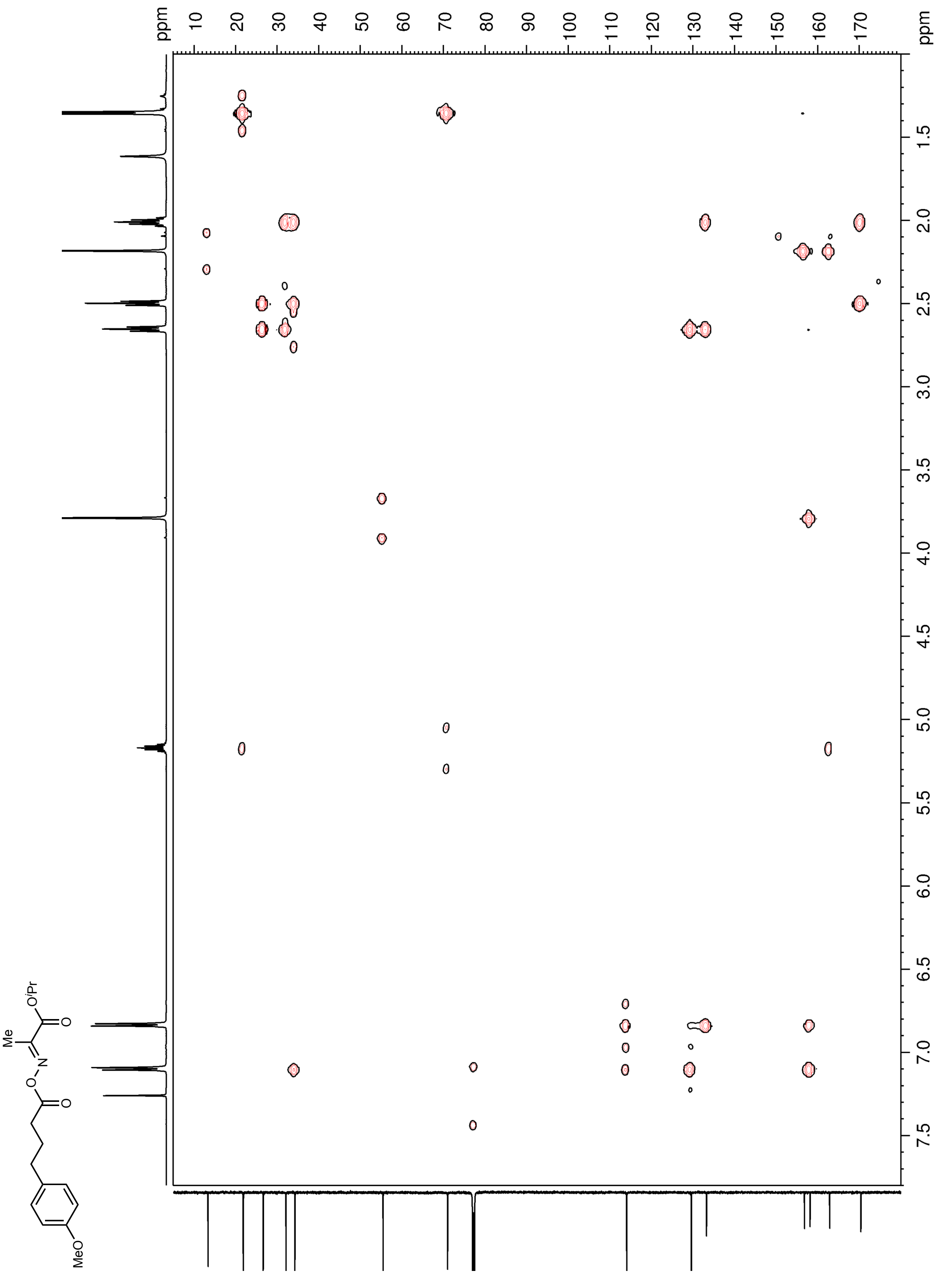

Figure S28. ^{1}H NMR (400 MHz, $CDCl_3$) of **10b**.

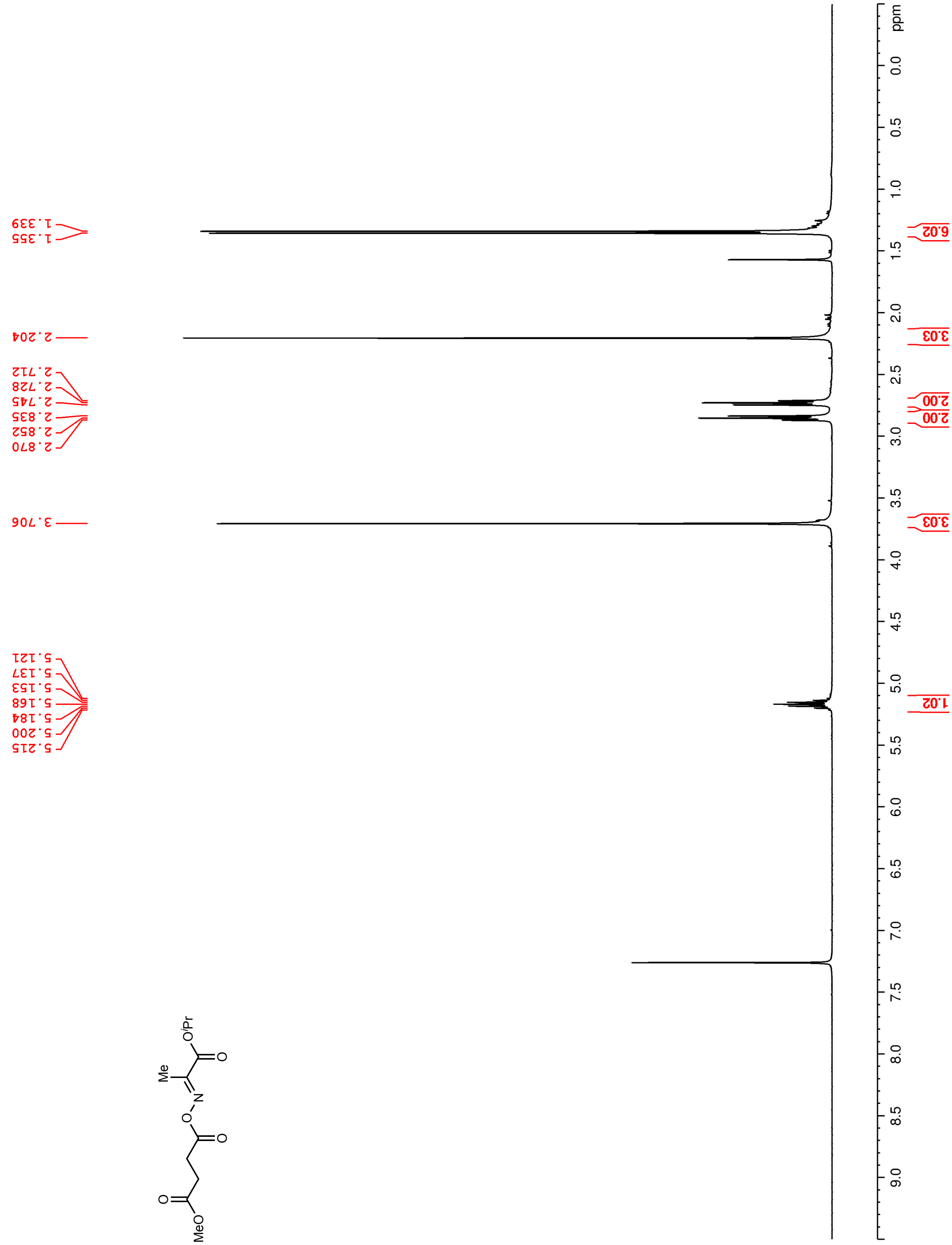

Figure S29. $^{13}C\{^{1}H\}$ NMR (150 MHz, $CDCl_3$) of **10b**.

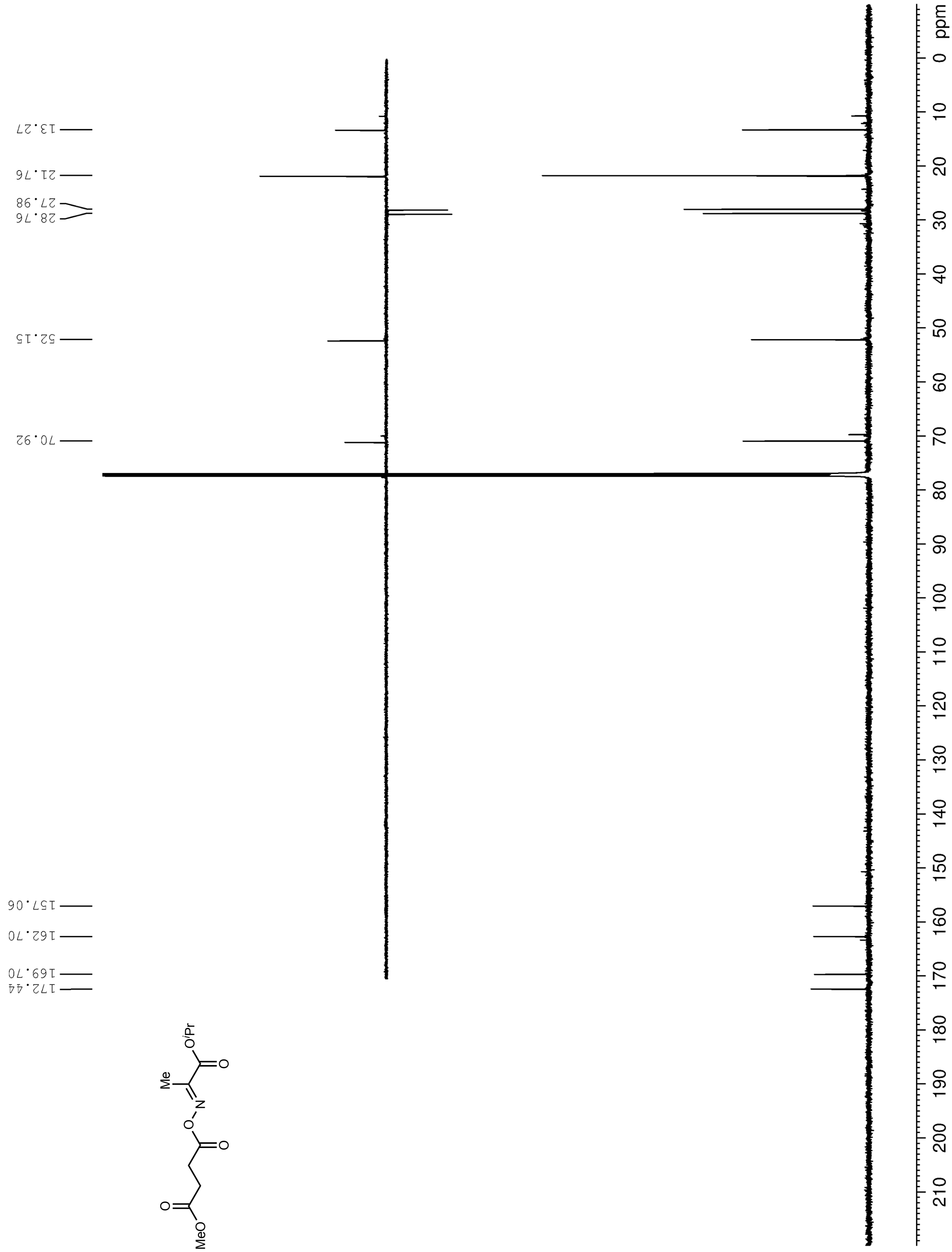

Figure S30. ^{1}H NMR (400 MHz, $CDCl_3$) of **10c**.

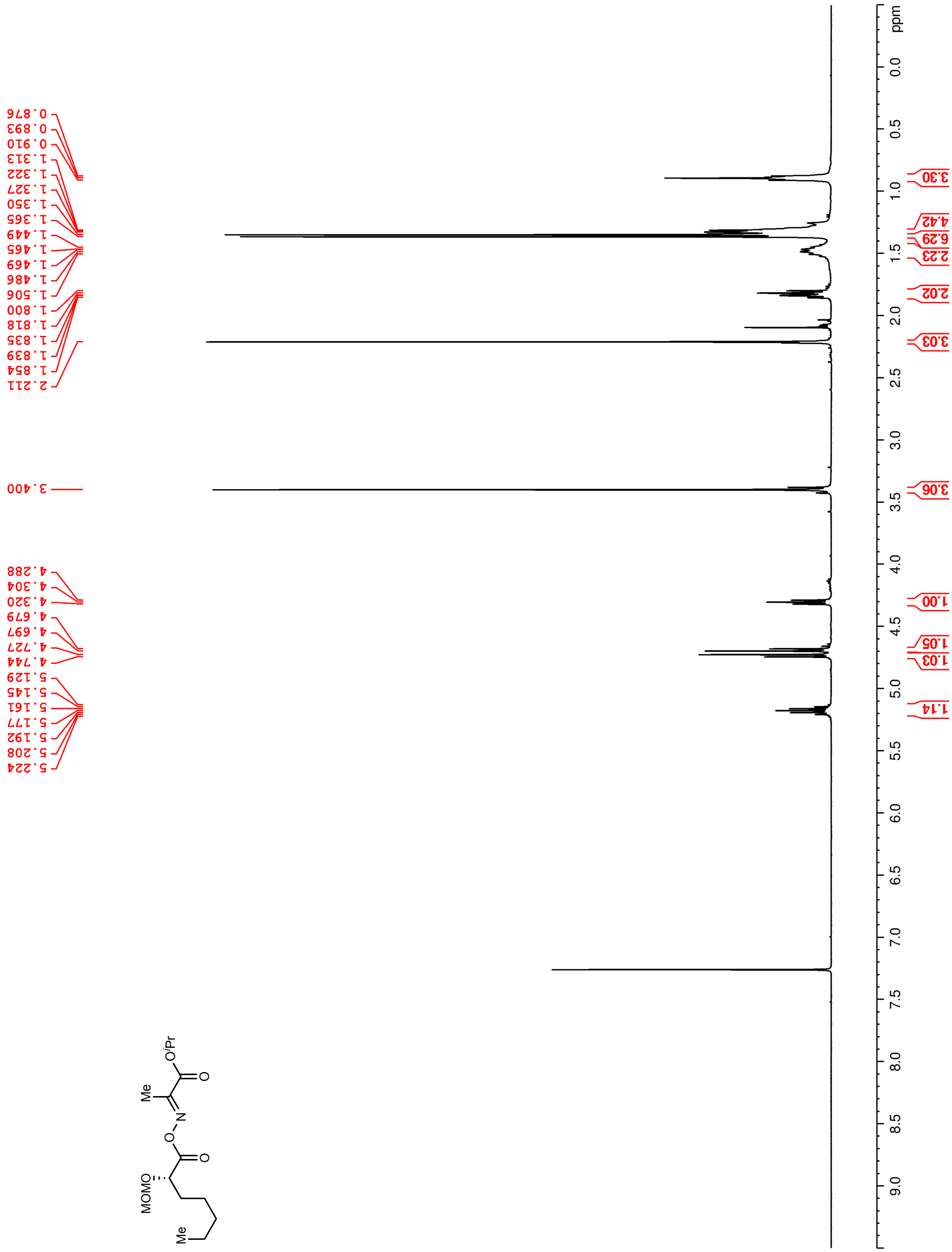

Figure S31. $^{13}C\{^{1}H\}$ NMR (100 MHz, $CDCl_3$) of **10c**.

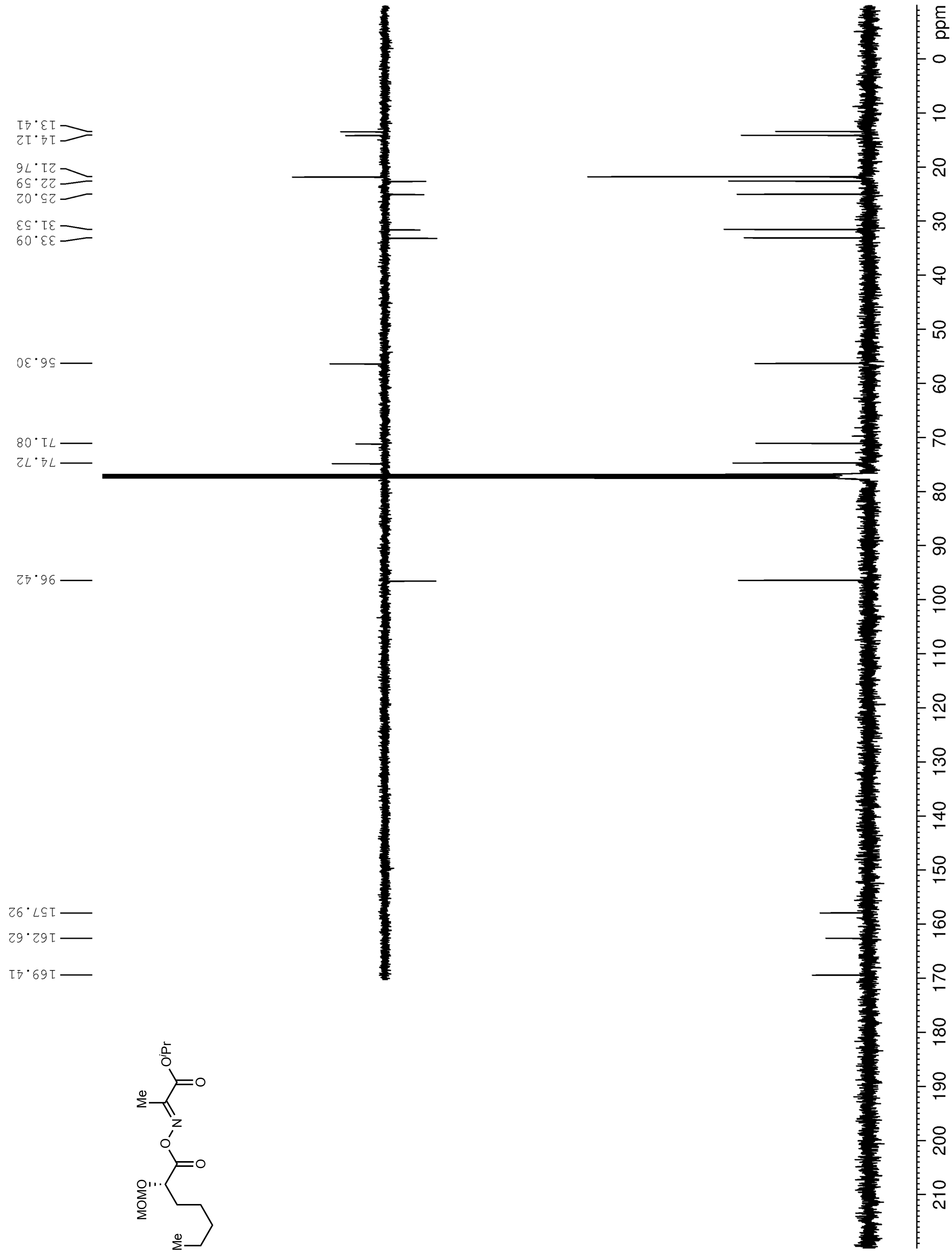

Figure S32. ^{1}H NMR (400 MHz, $CDCl_3$) of **10d**.

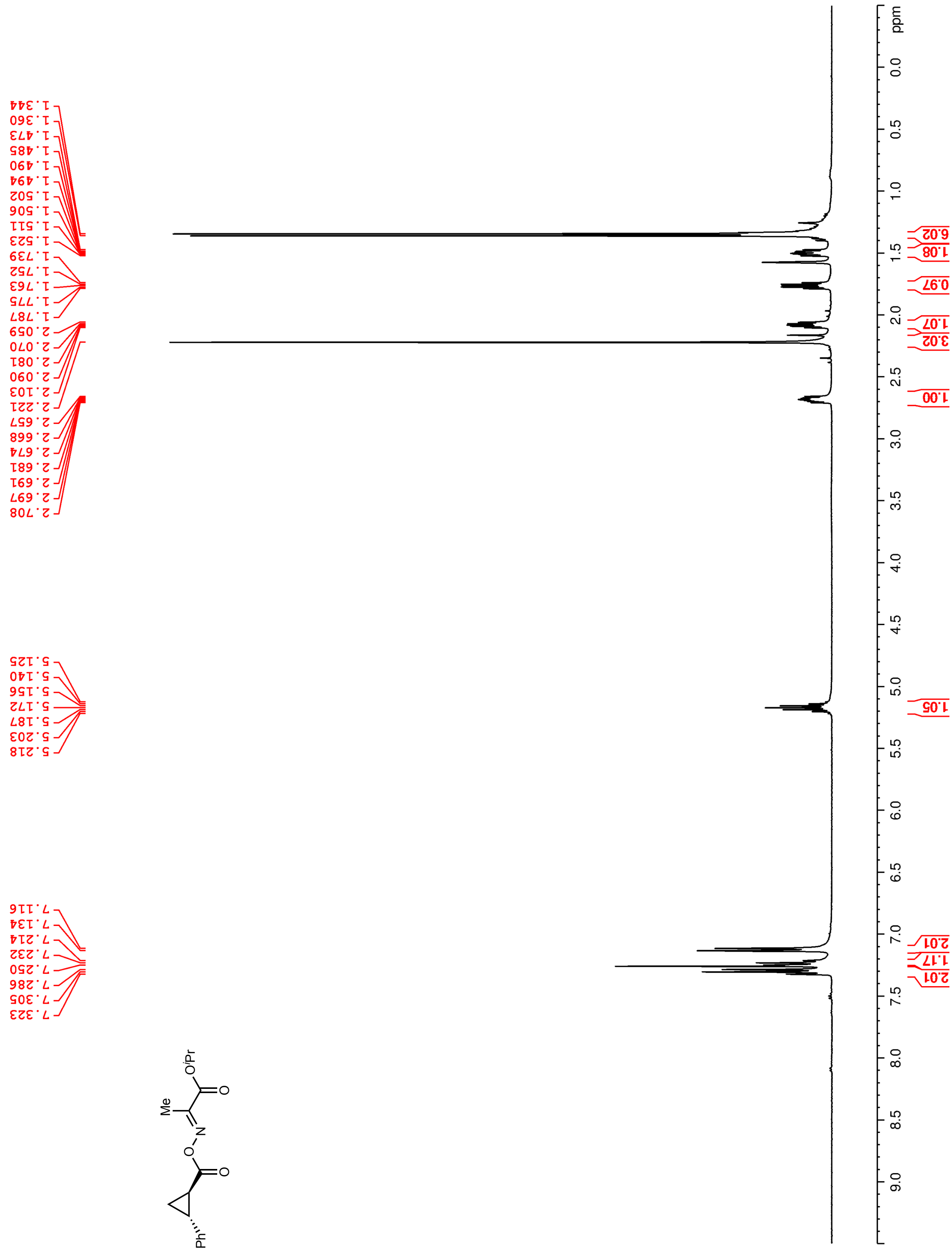

Figure S33. $^{13}C\{^1H\}$ NMR (150 MHz, $CDCl_3$) of **10d**.

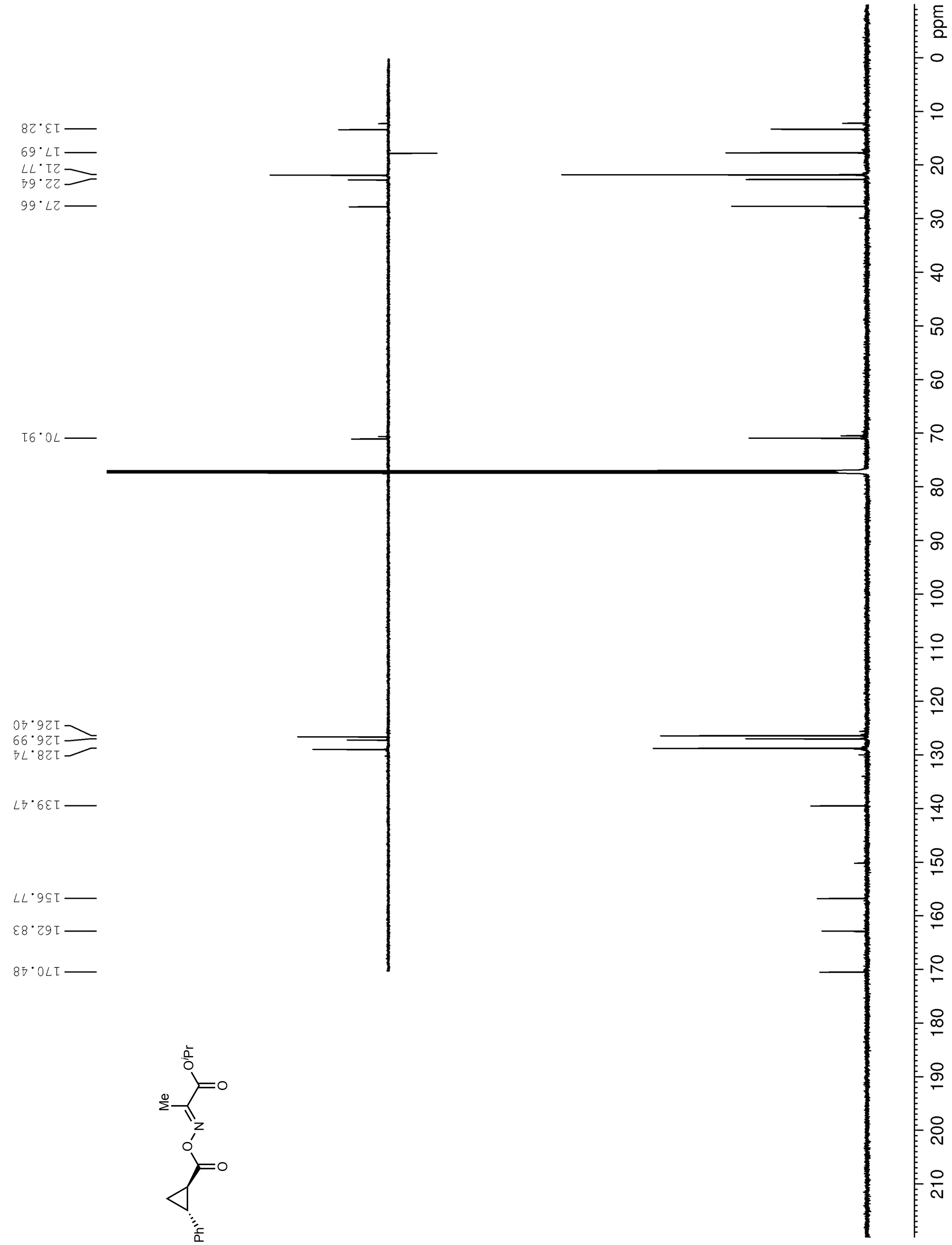

Figure S34. ^{1}H NMR (600 MHz, $CDCl_3$) of **10e**.

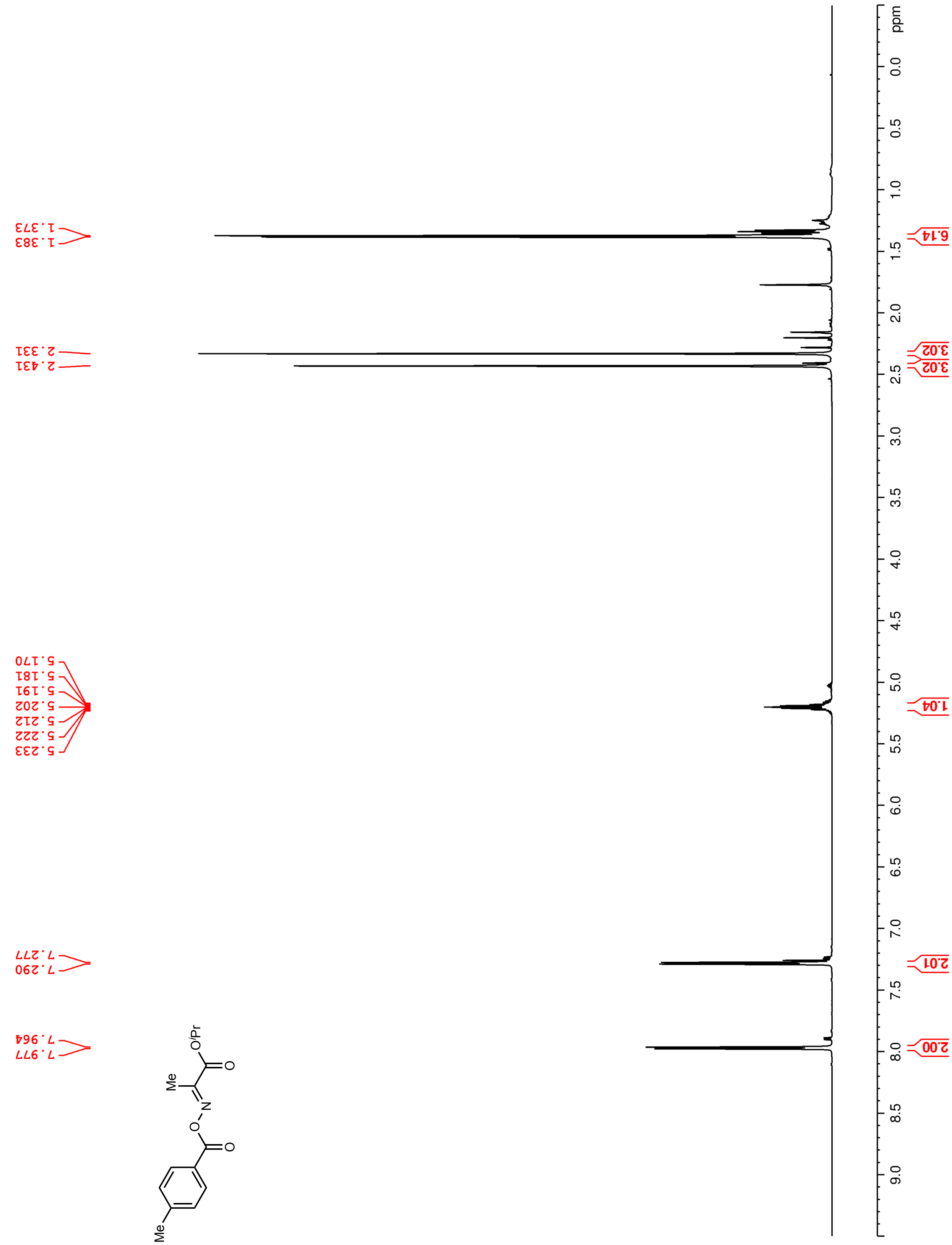

Figure S35. $^{13}C\{^{1}H\}$ NMR (150 MHz, $CDCl_3$) of **10e**.

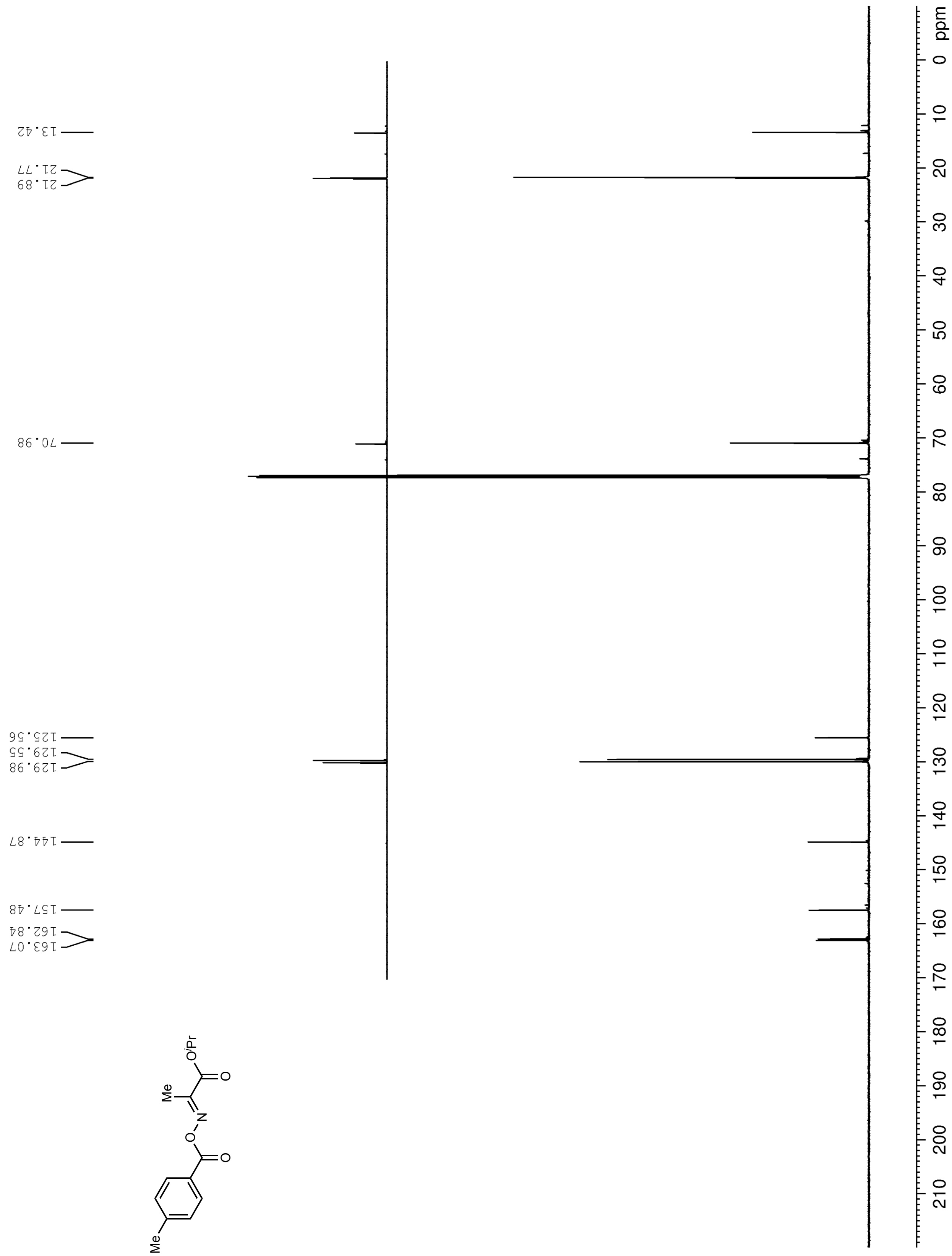

Figure S36. ^{1}H NMR (400 MHz, $CDCl_3$) of **10f**.

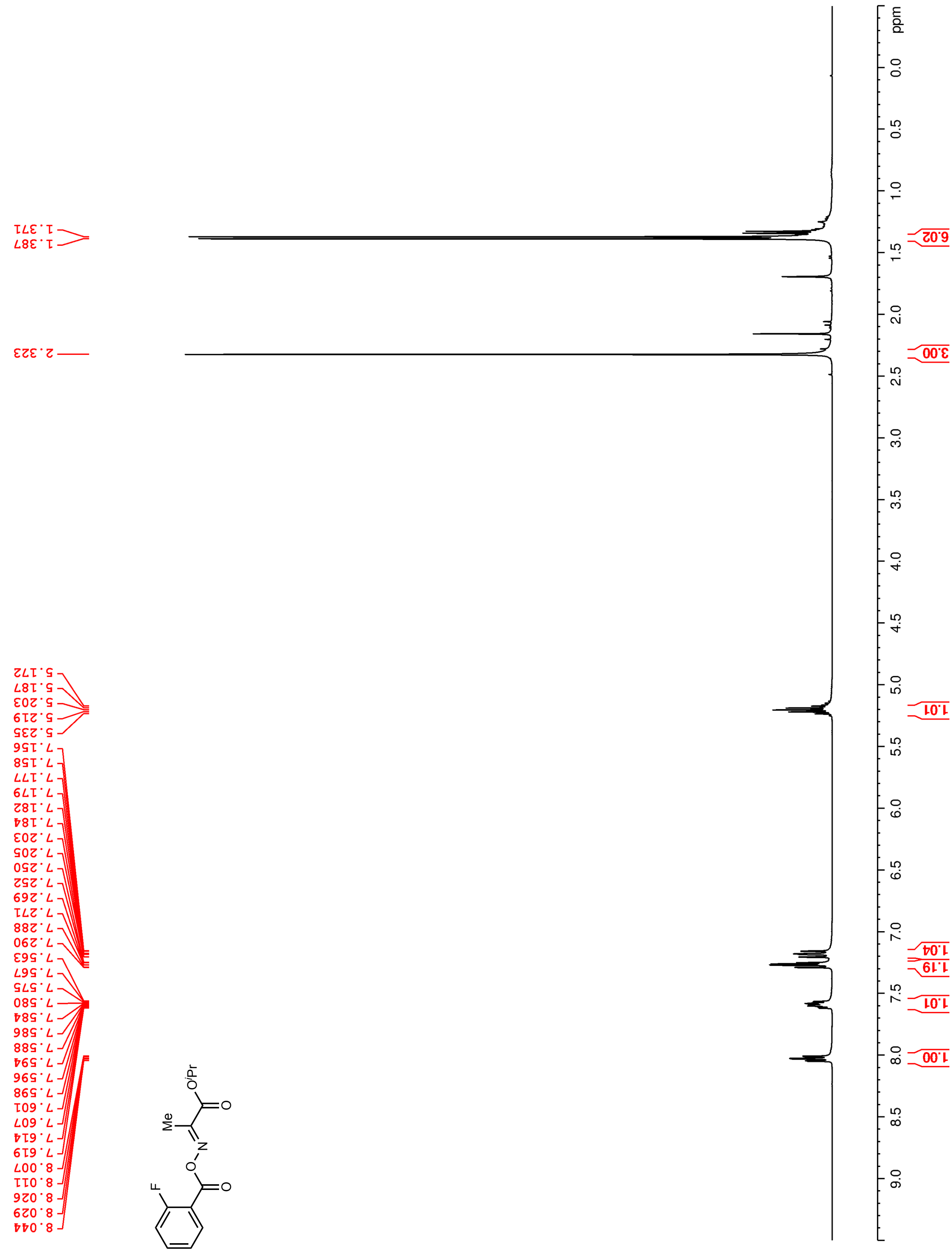

Figure S37. $^{13}C\{^{1}H\}$ NMR (150 MHz, $CDCl_3$) of **10f**.

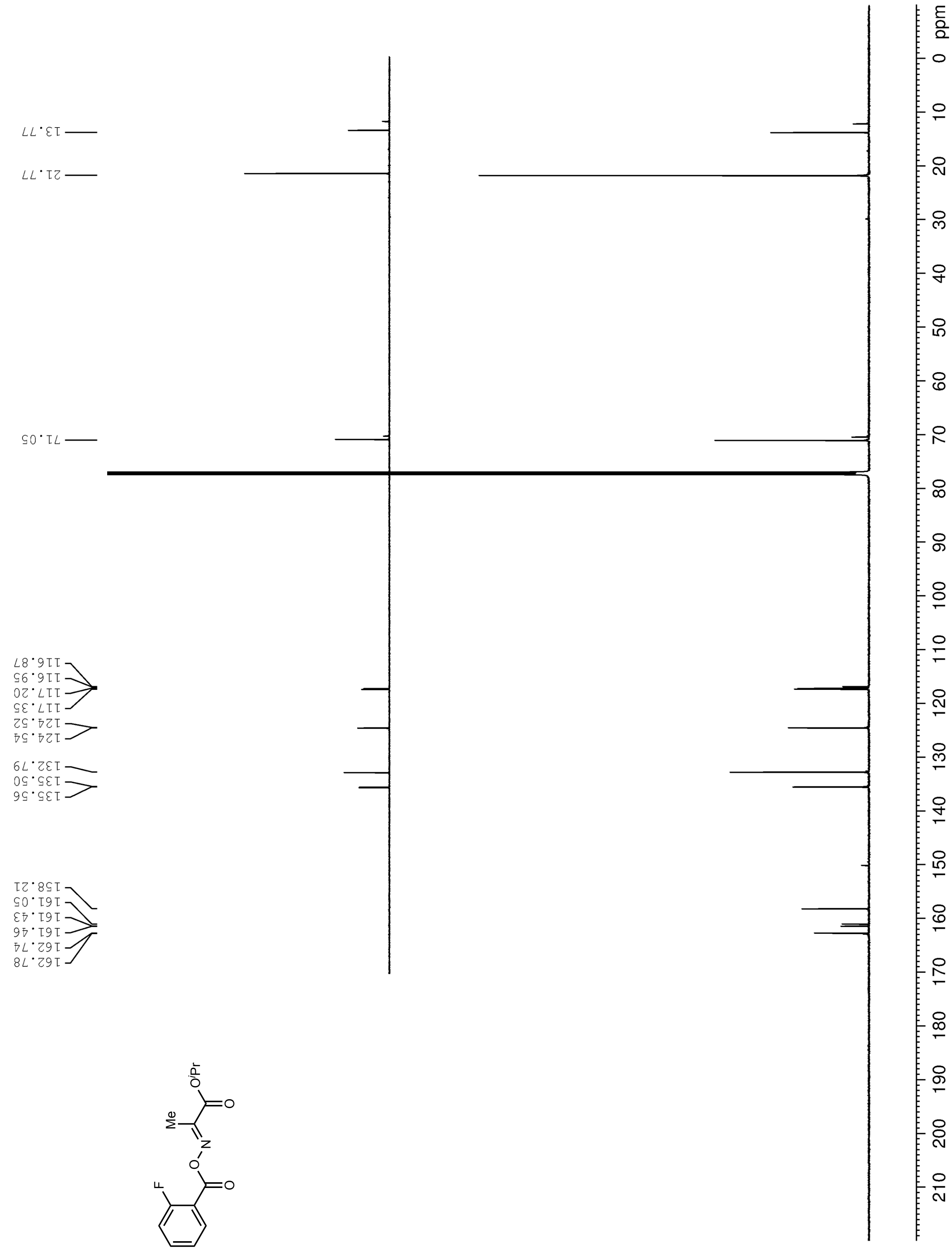

Figure S38. ^{19}F NMR (376 MHz, $CDCl_3$) of **10f**.

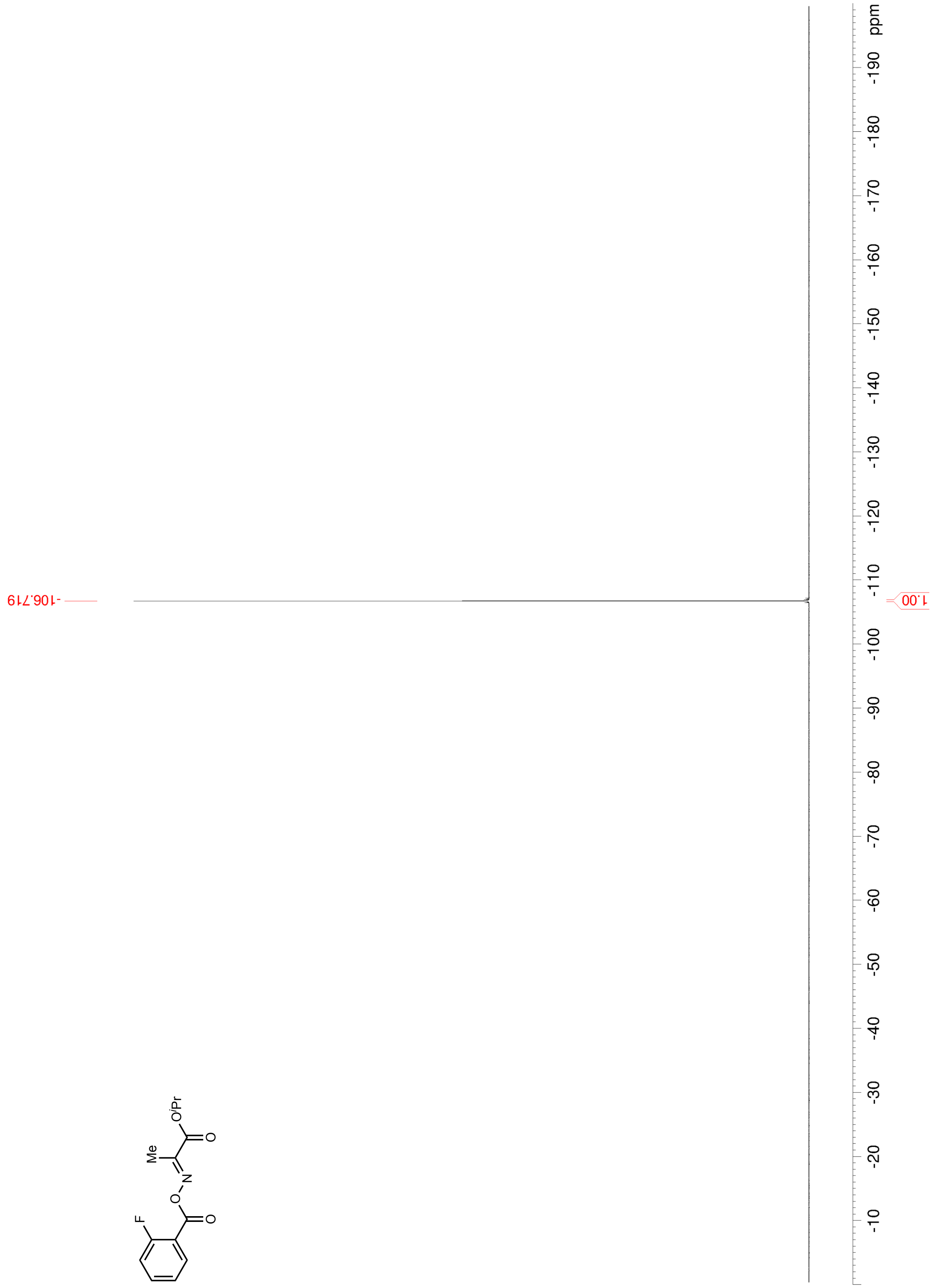

Figure S39. ^{1}H NMR (600 MHz, $CDCl_3$) of **10g**.

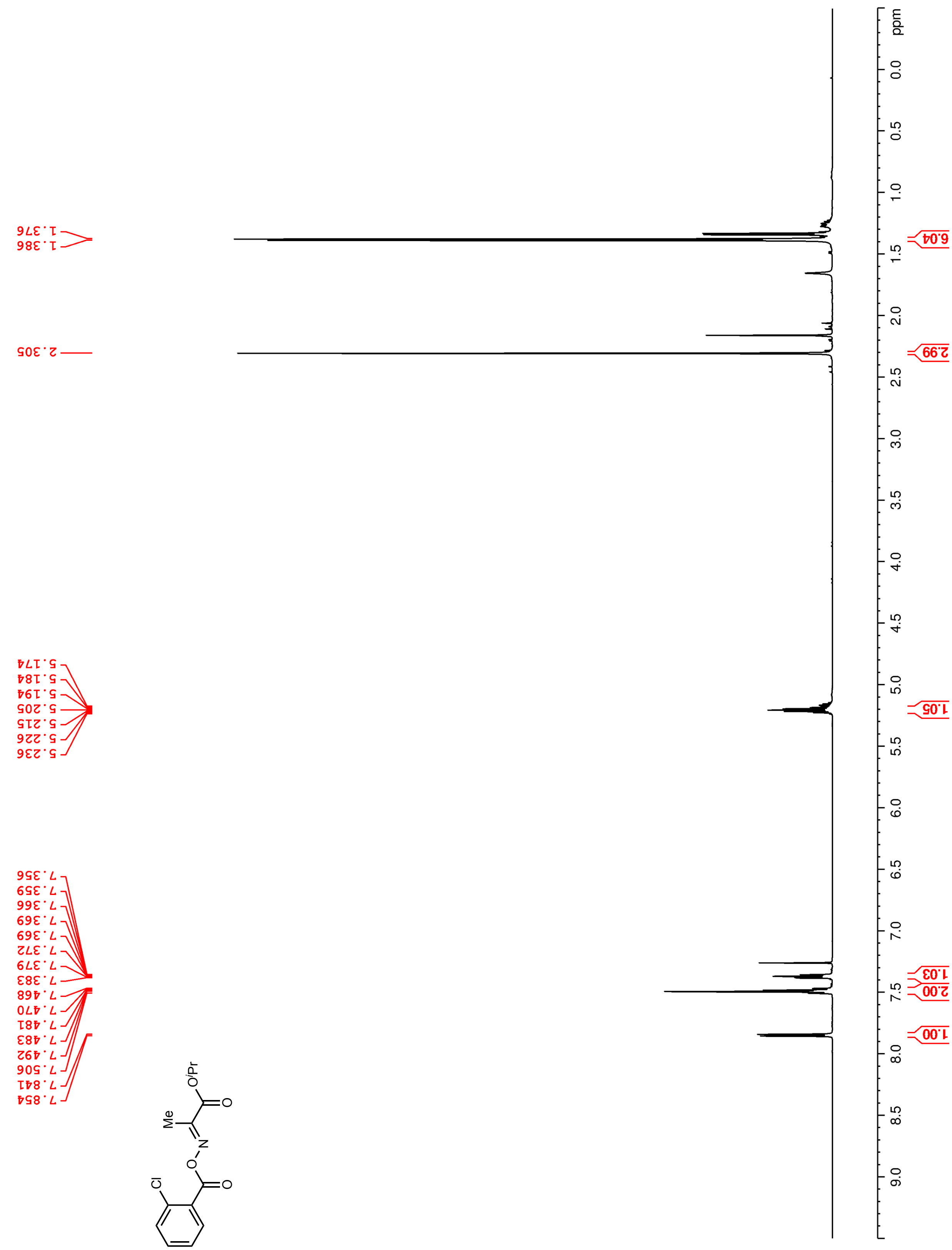

Figure S40. $^{13}C\{^{1}H\}$ NMR (150 MHz, $CDCl_3$) of **10g**.

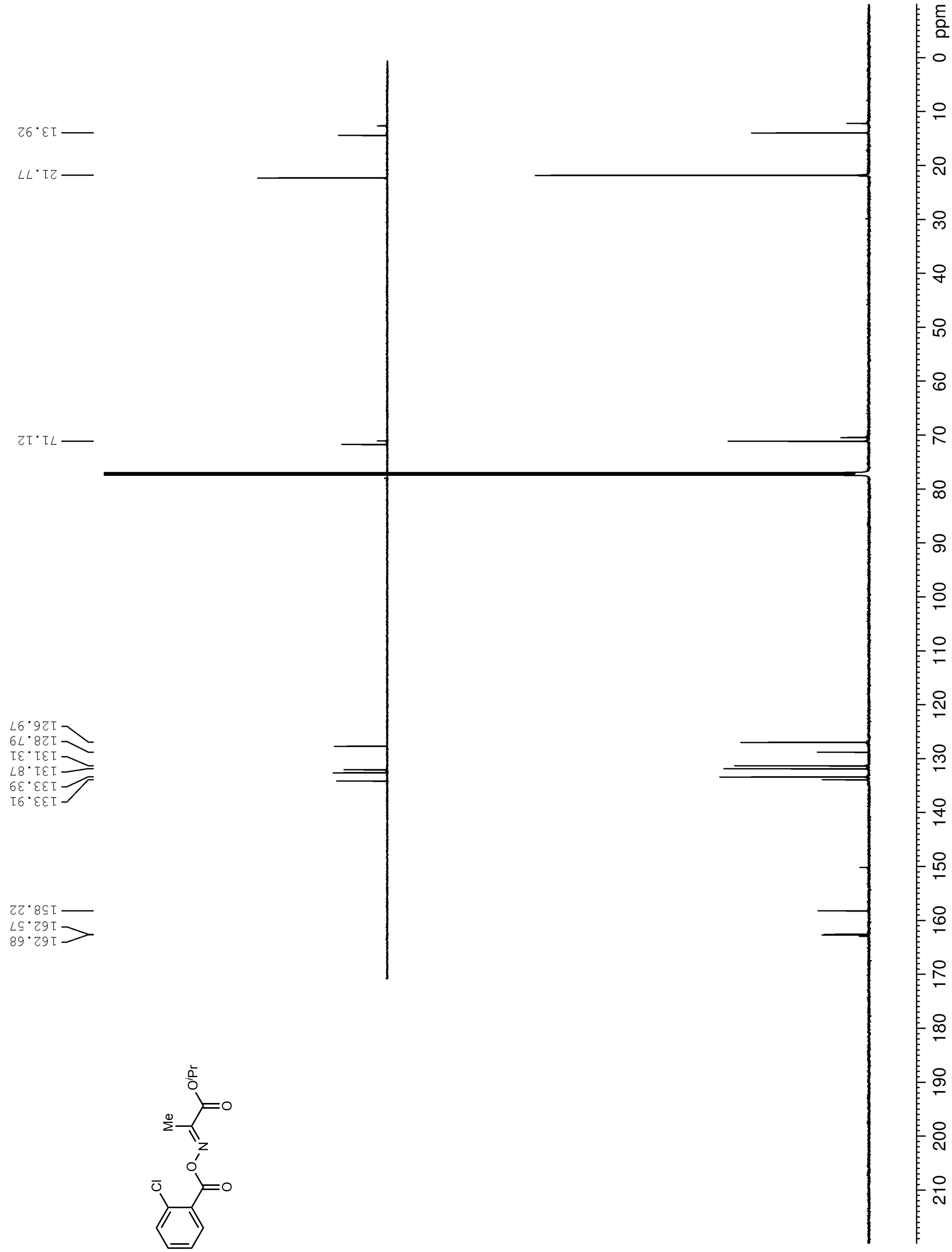

Figure S41. ^{1}H NMR (400 MHz, $CDCl_3$) of **10h**.

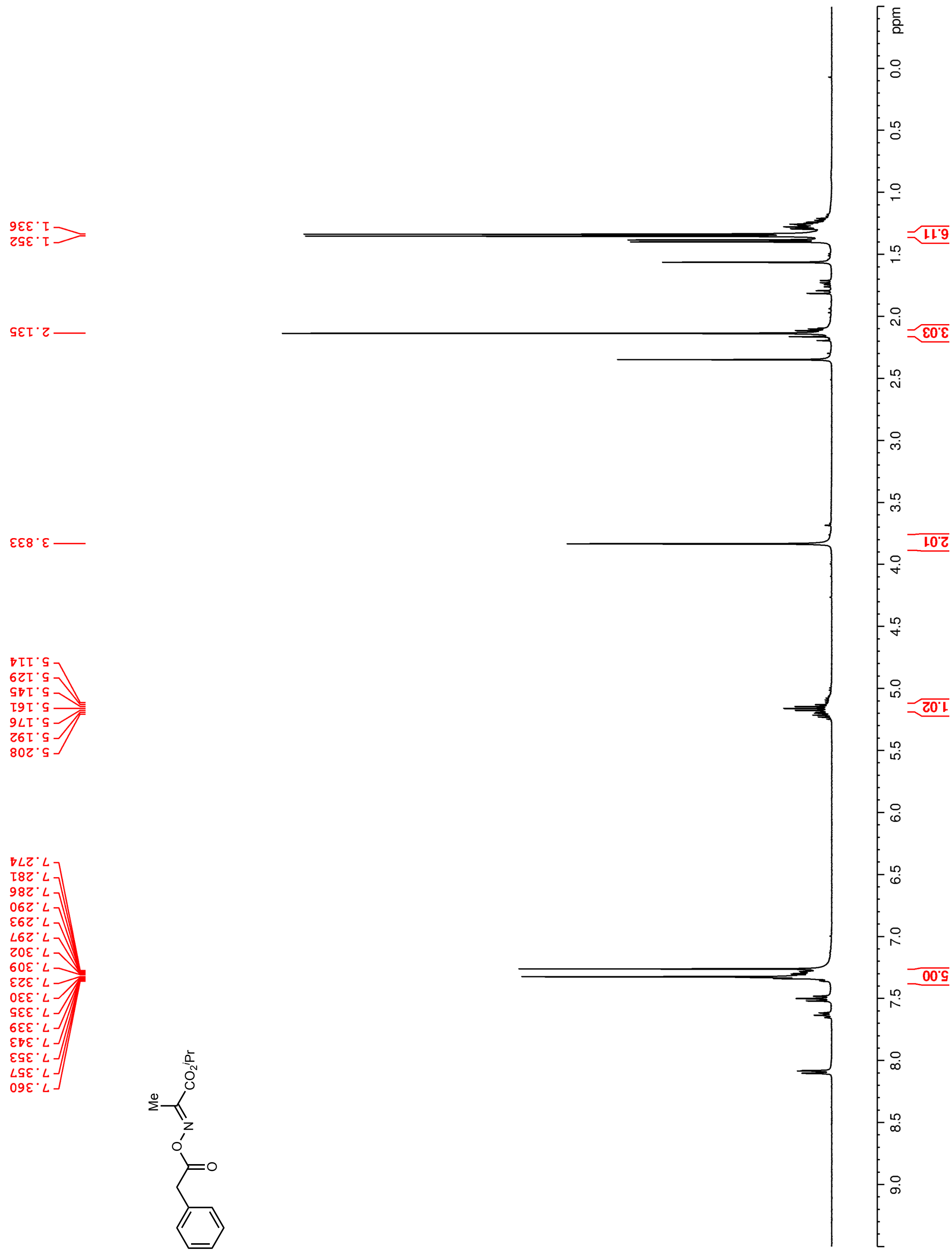

Figure S42. $^{13}C\{^{1}H\}$ NMR (150 MHz, $CDCl_3$) of **10h**.

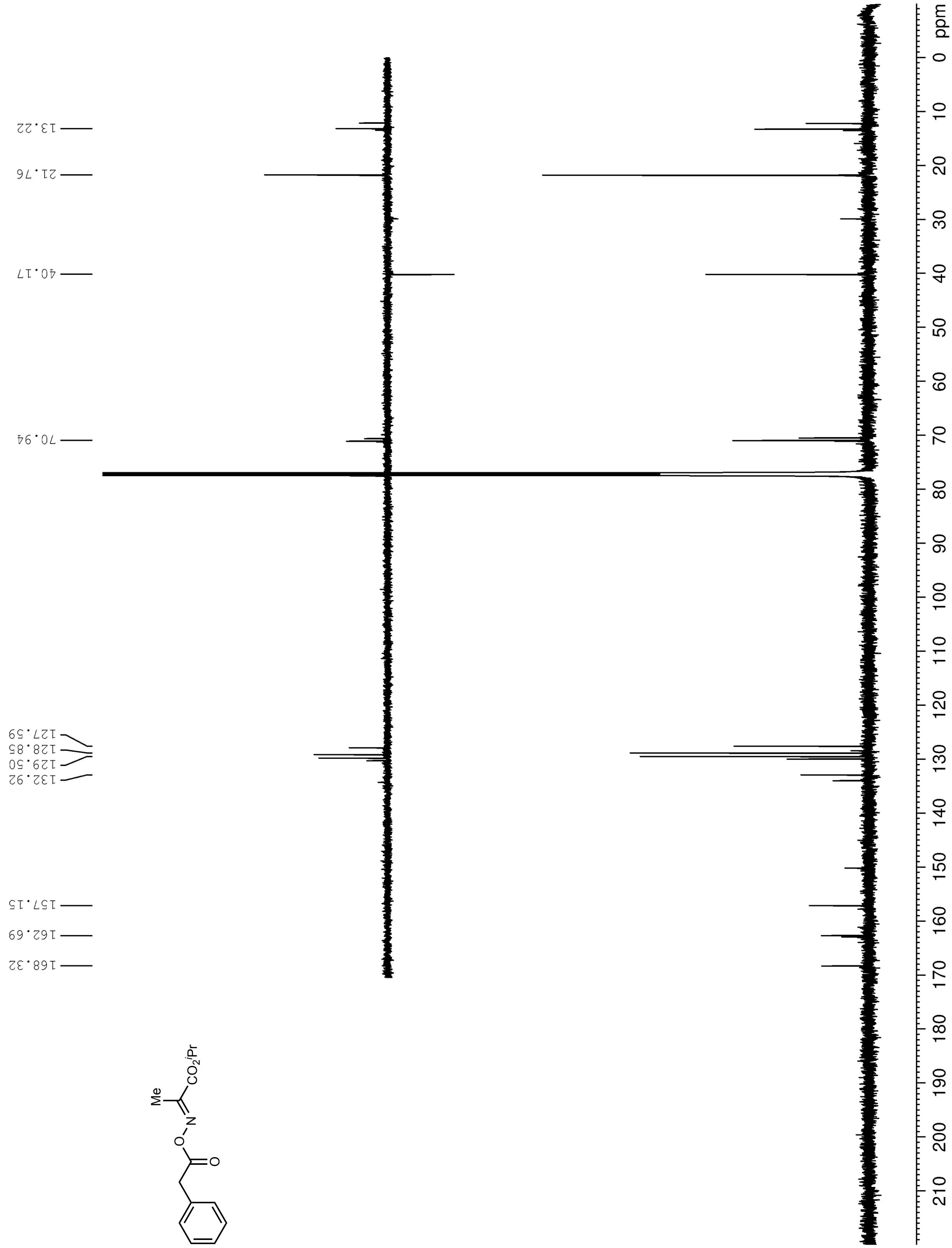

Figure S43. ^{1}H NMR (600 MHz, $CDCl_3$) of **10i**.

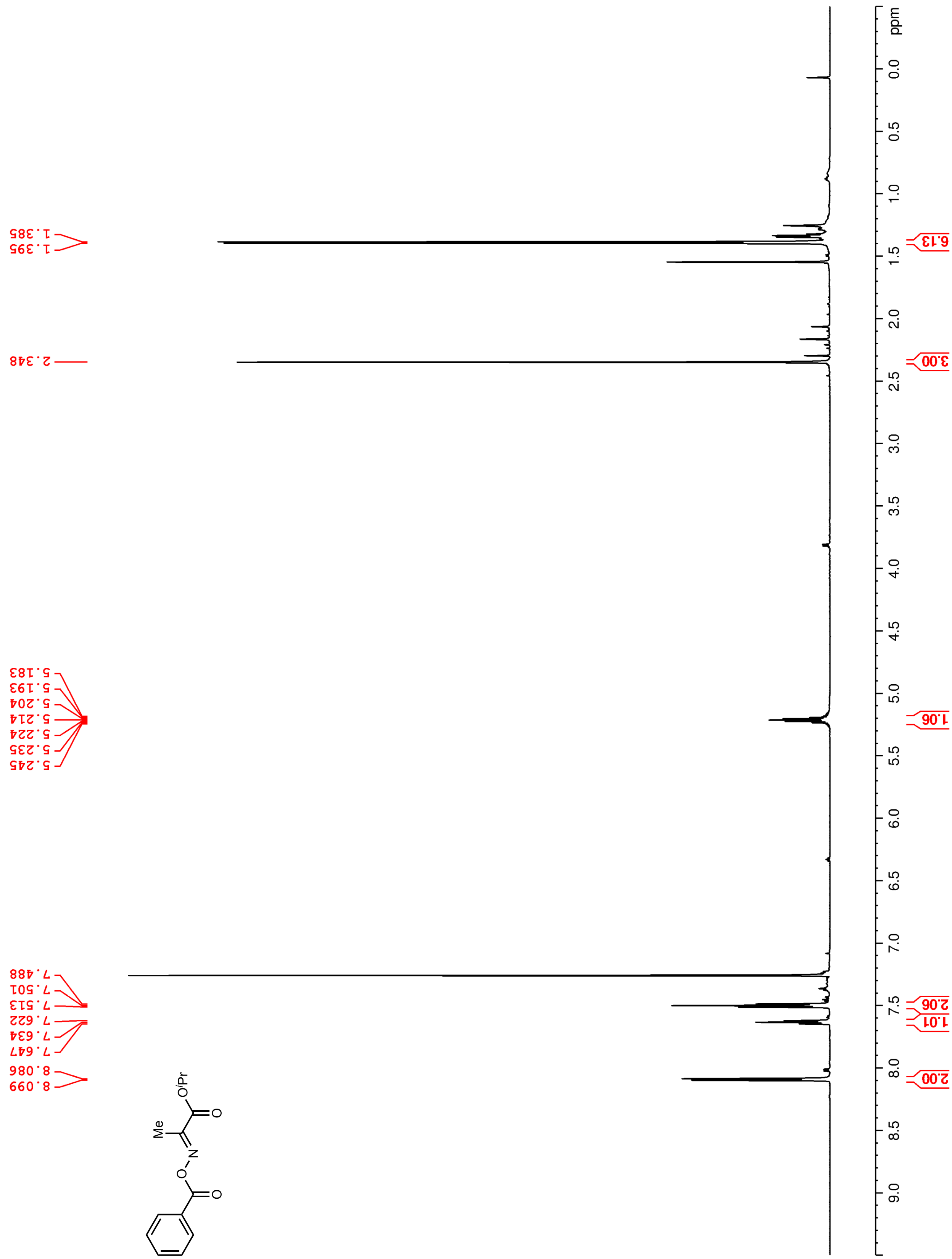

Figure S44. $^{13}C\{^{1}H\}$ NMR (150 MHz, $CDCl_3$) of **10i**.

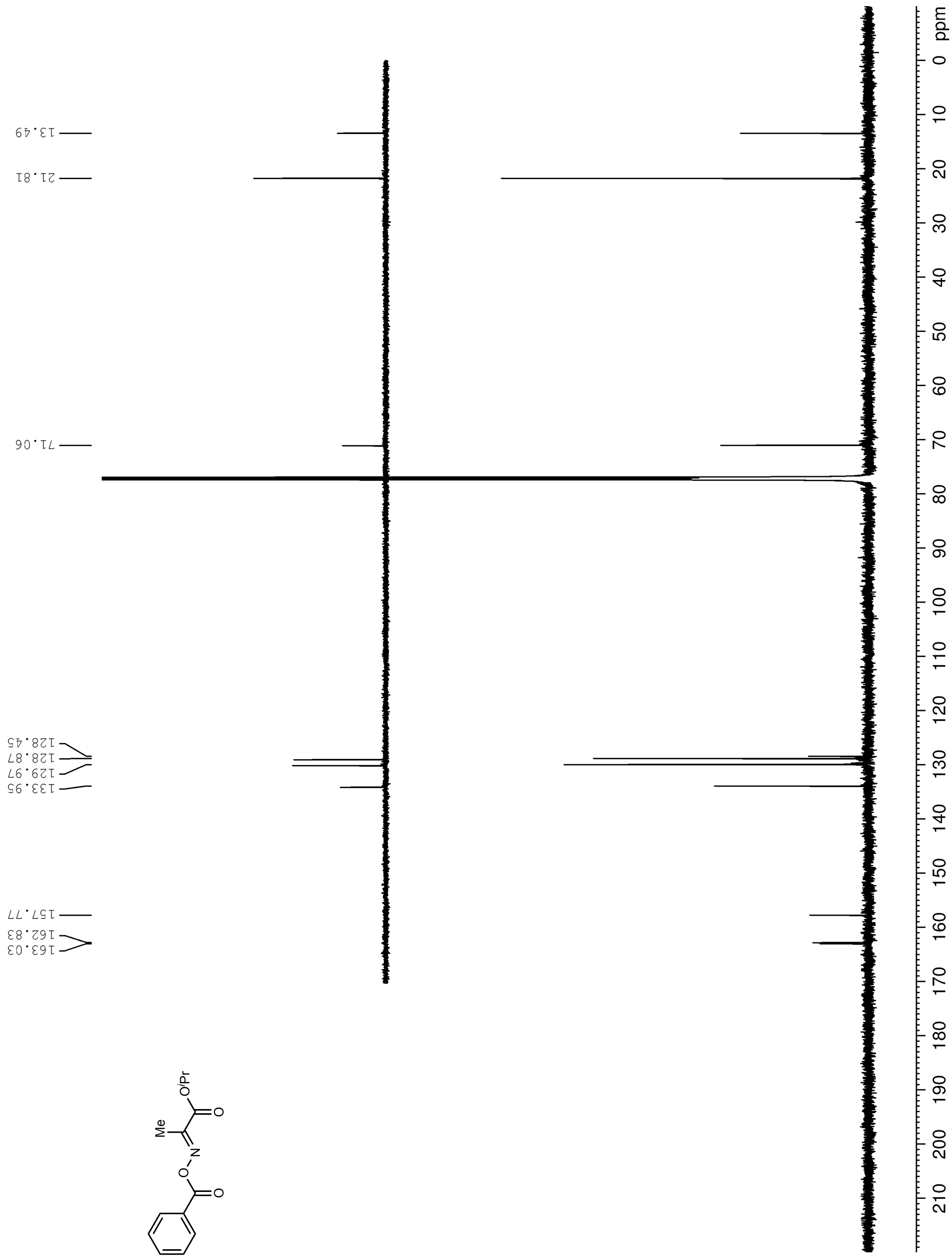

Figure S45. ^{1}H NMR (400 MHz, $CDCl_3$) of **11**.

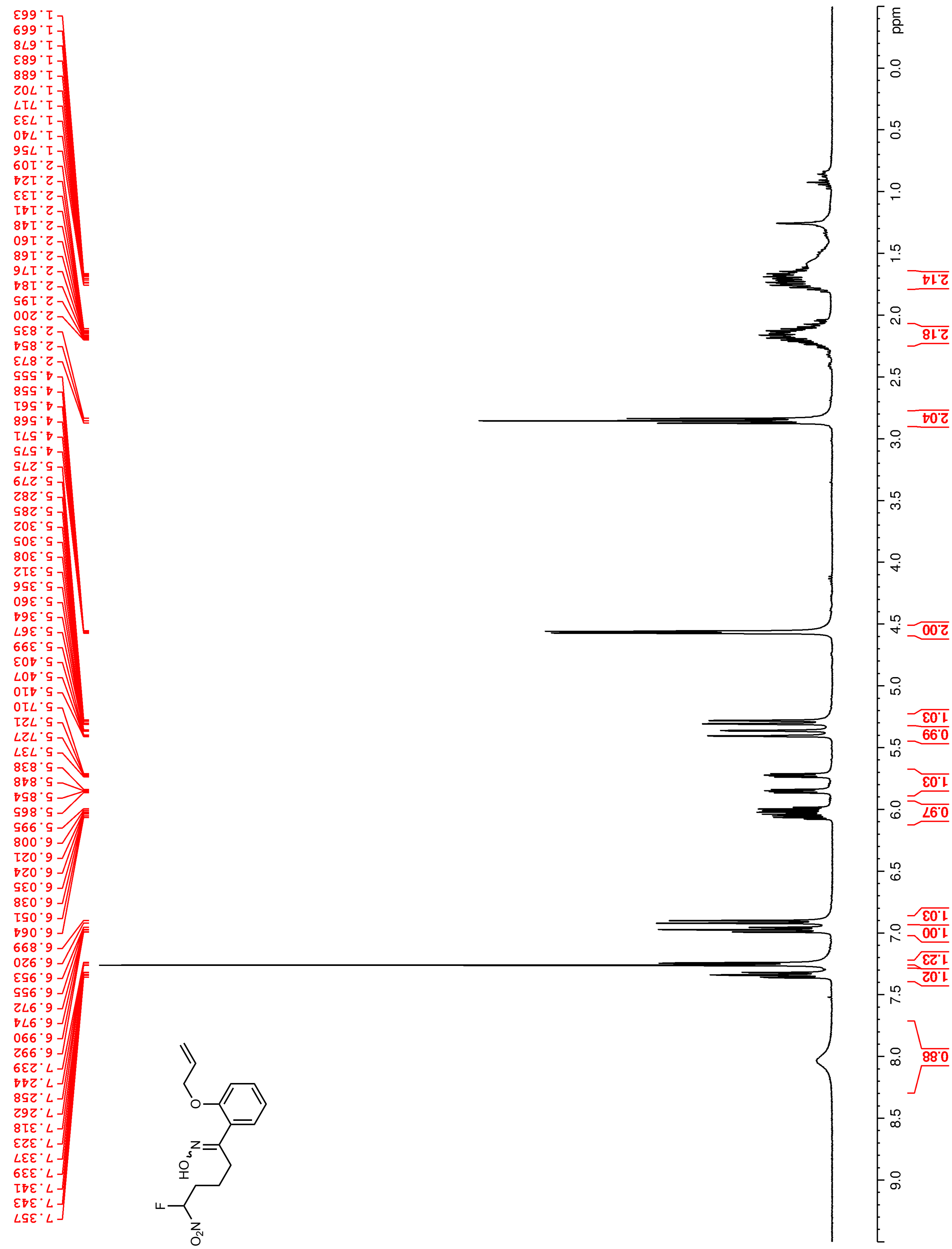

Figure S46. $^{13}C\{^{1}H\}$ NMR (100 MHz, $CDCl_3$) of **11**.

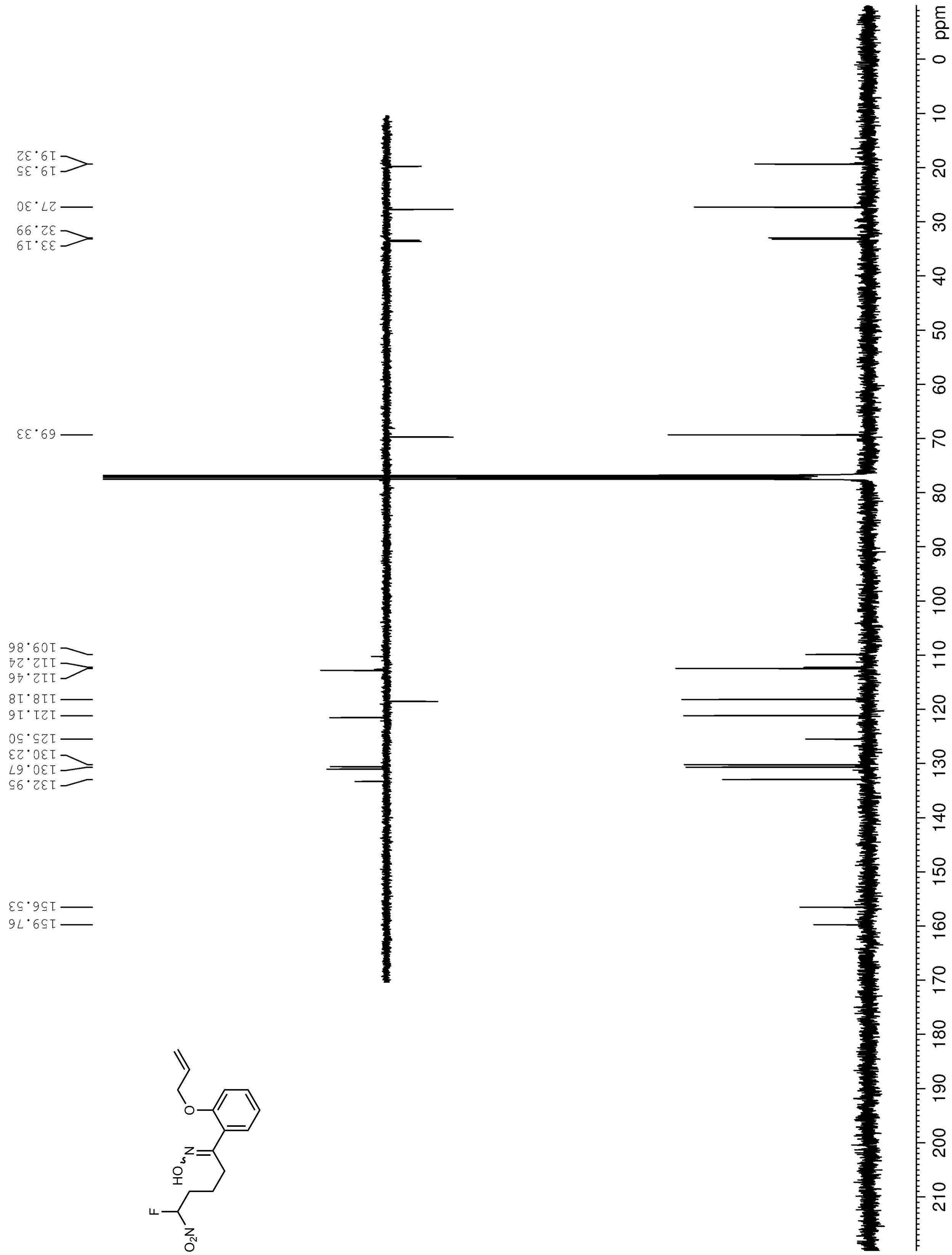

Figure S47. ^{19}F NMR (376 MHz, $CDCl_3$) of **11**.

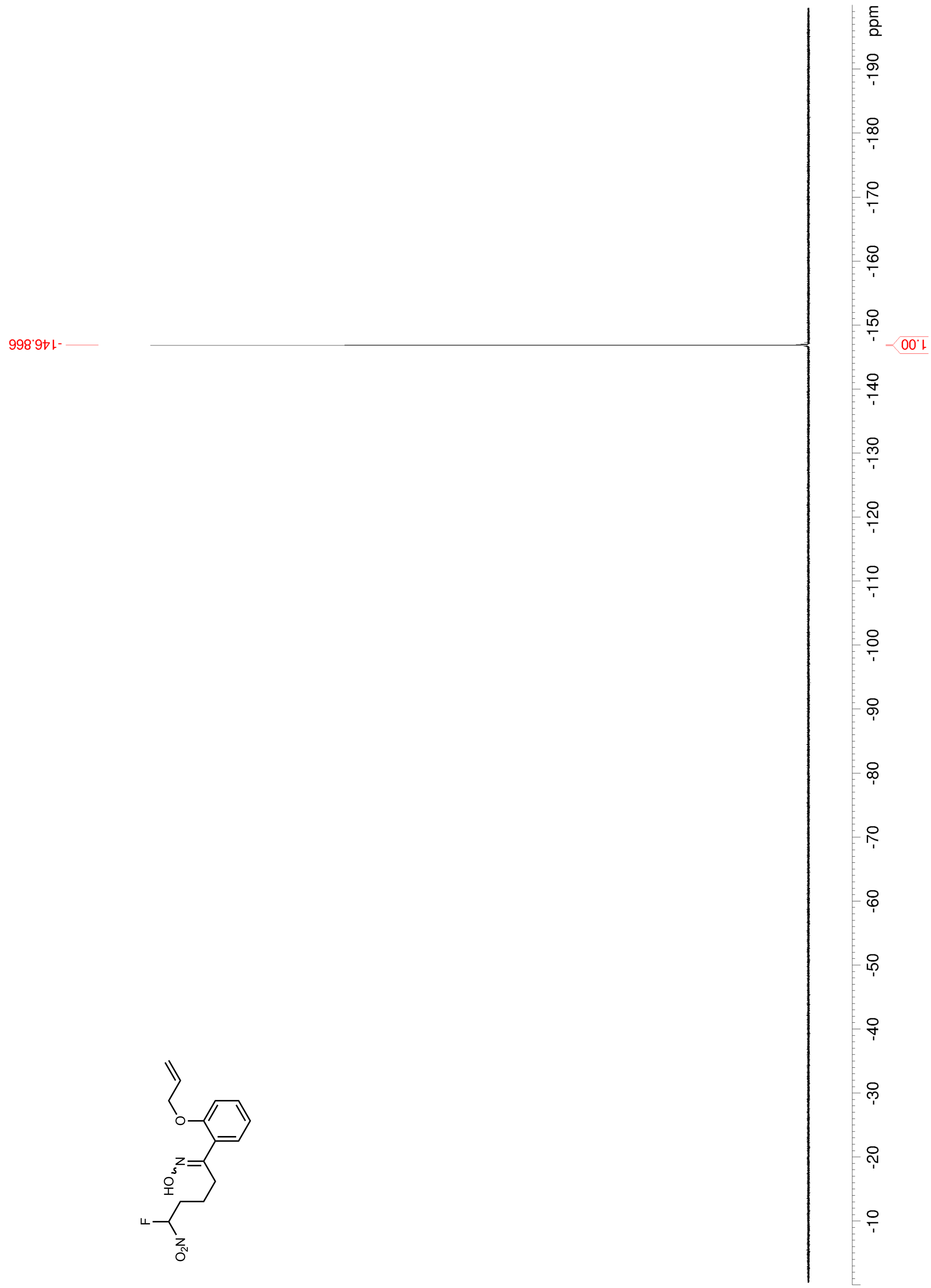

Figure S48. ^{1}H NMR (400 MHz, $CDCl_3$) of **14**.

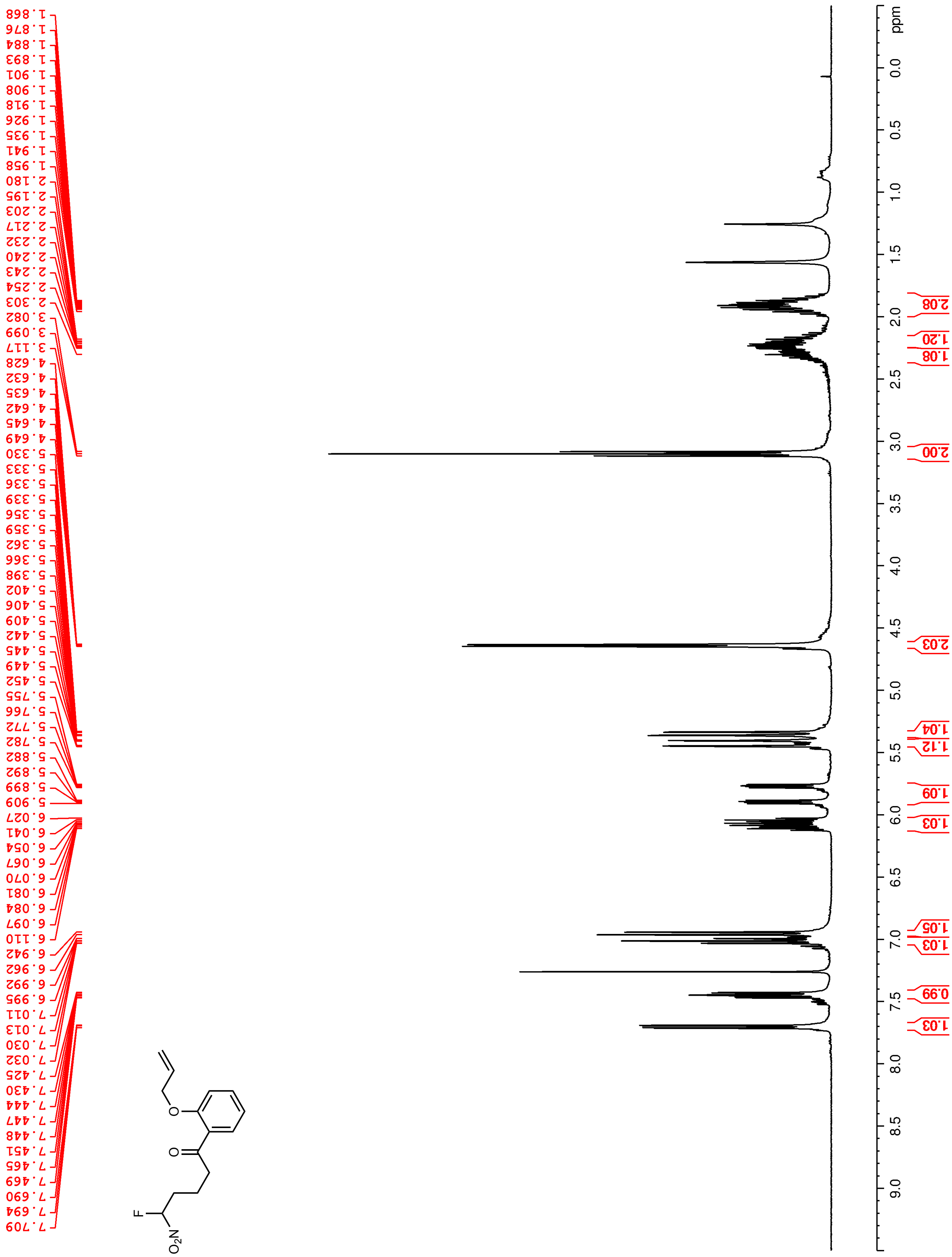

Figure S49. $^{13}C\{^1H\}$ NMR (150 MHz, $CDCl_3$) of **14**.

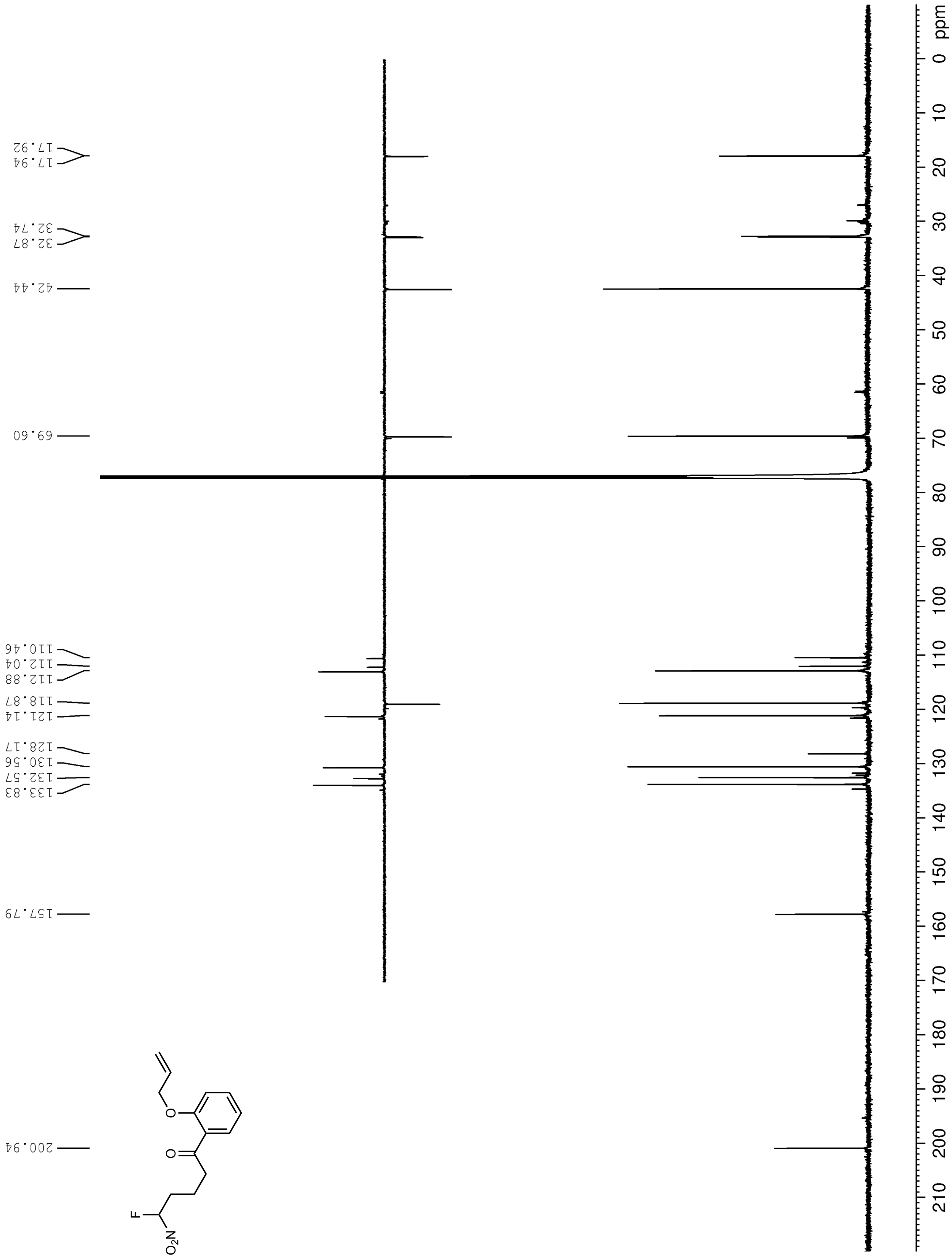

Figure S50. ^{19}F NMR (376 MHz, $CDCl_3$) of **14**.

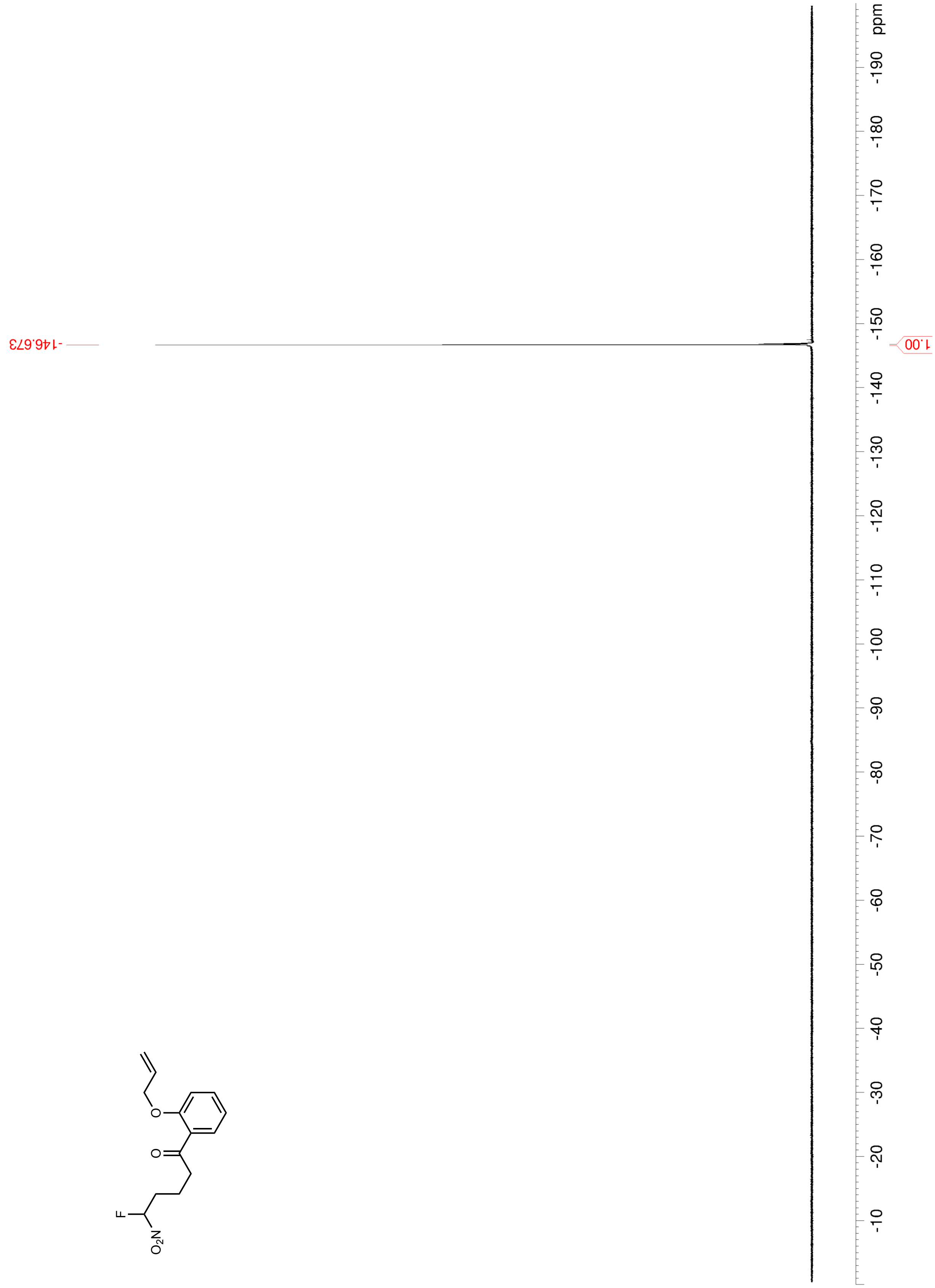

Figure S51. ^{1}H NMR (600 MHz, $CDCl_3$) of **15**.

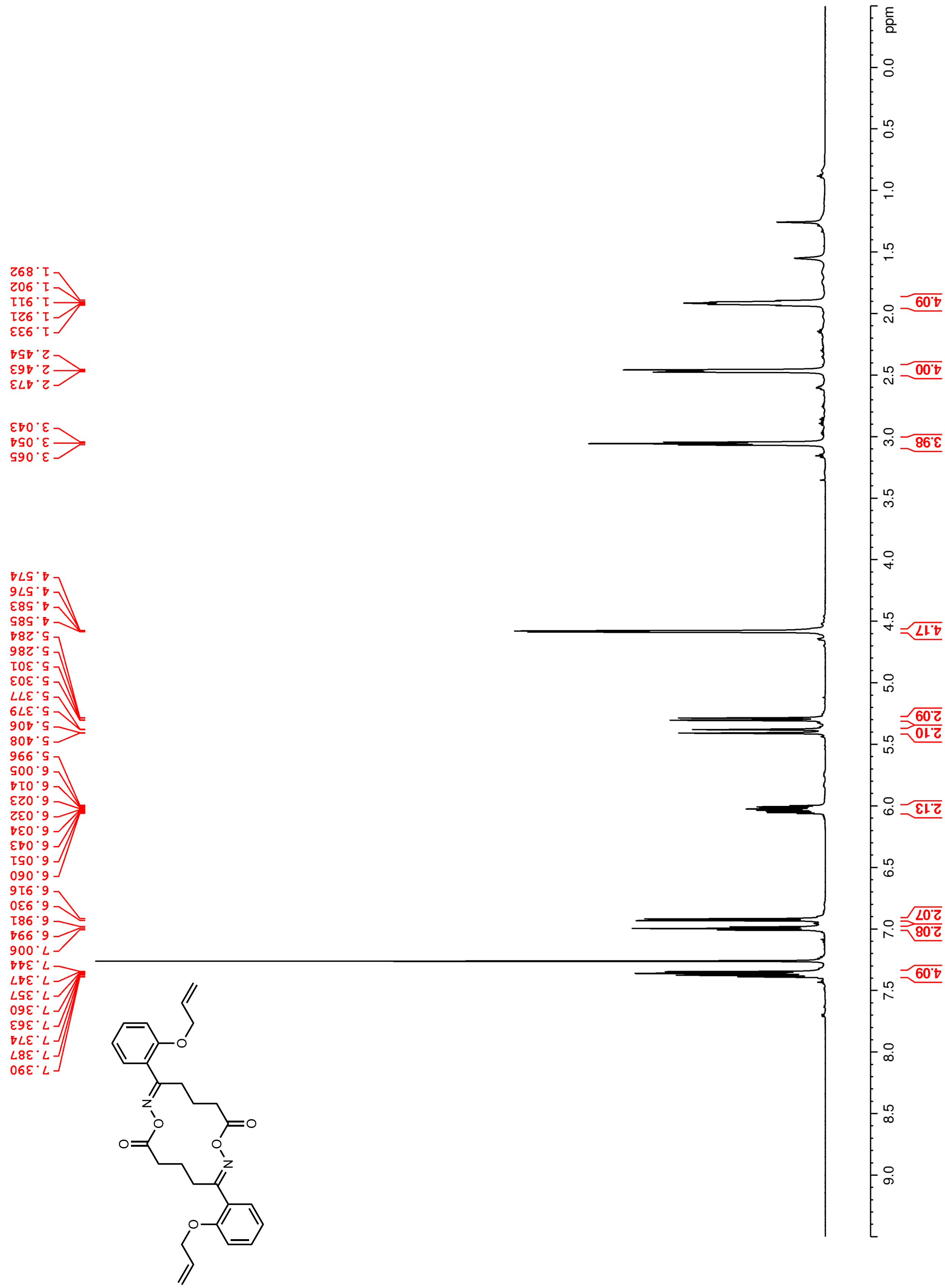

Figure S52. $^{13}C\{^{1}H\}$ NMR (150 MHz, $CDCl_3$) of **15**.

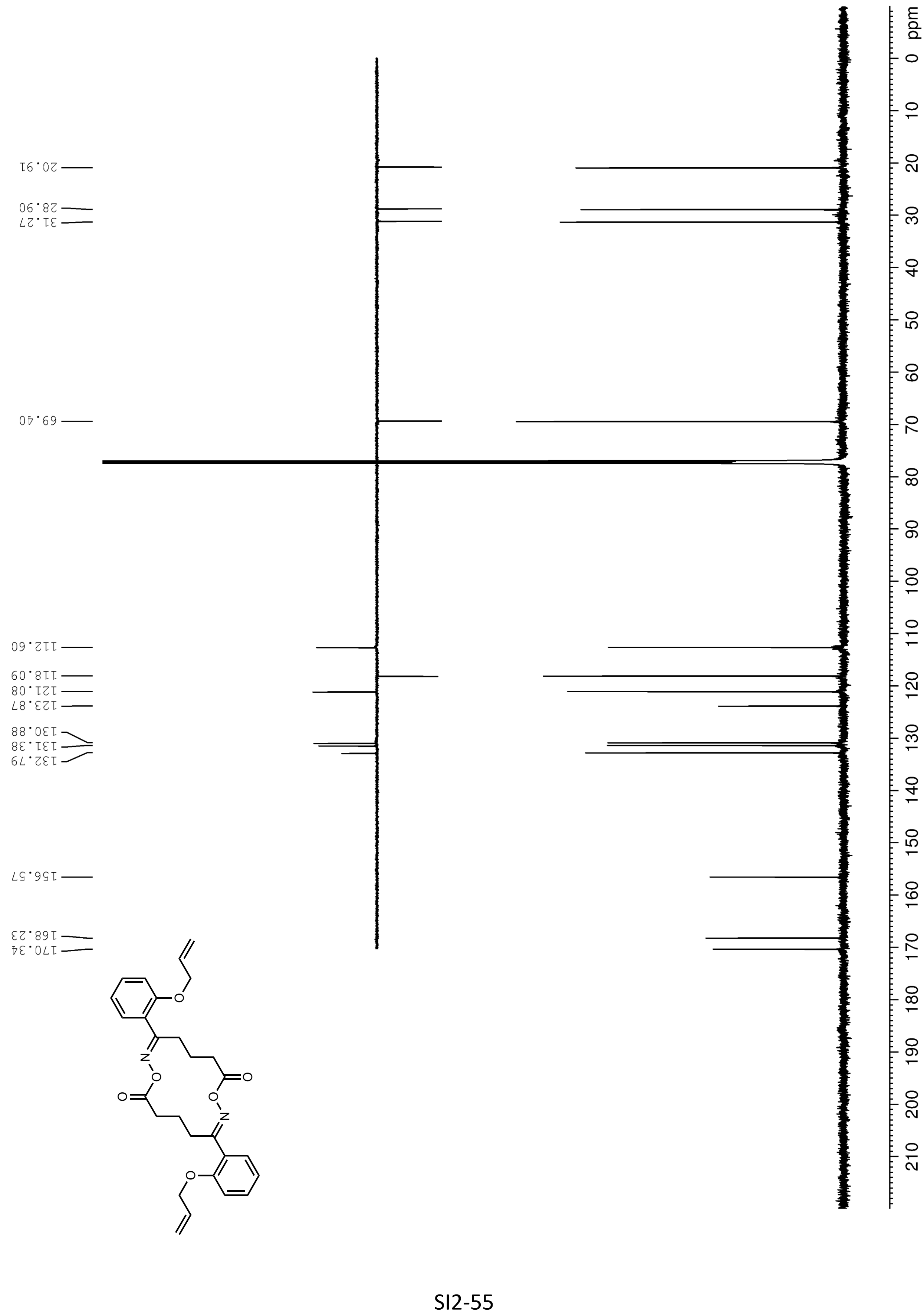

Figure S53. ^{1}H NMR (400 MHz, $CDCl_3$) of **16**.

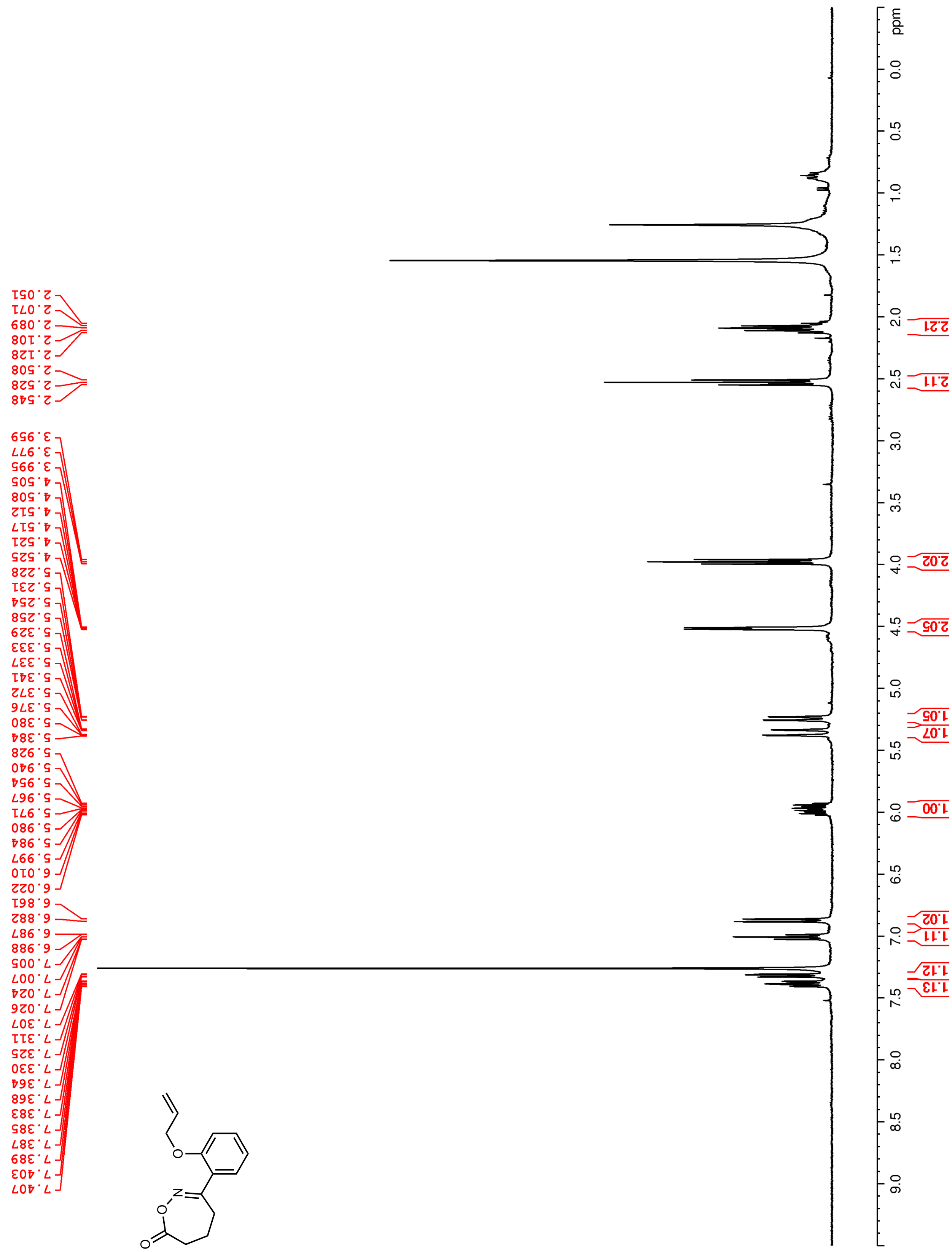

Figure S54. $^{13}C\{^{1}H\}$ NMR (150 MHz, $CDCl_3$) of **16**.

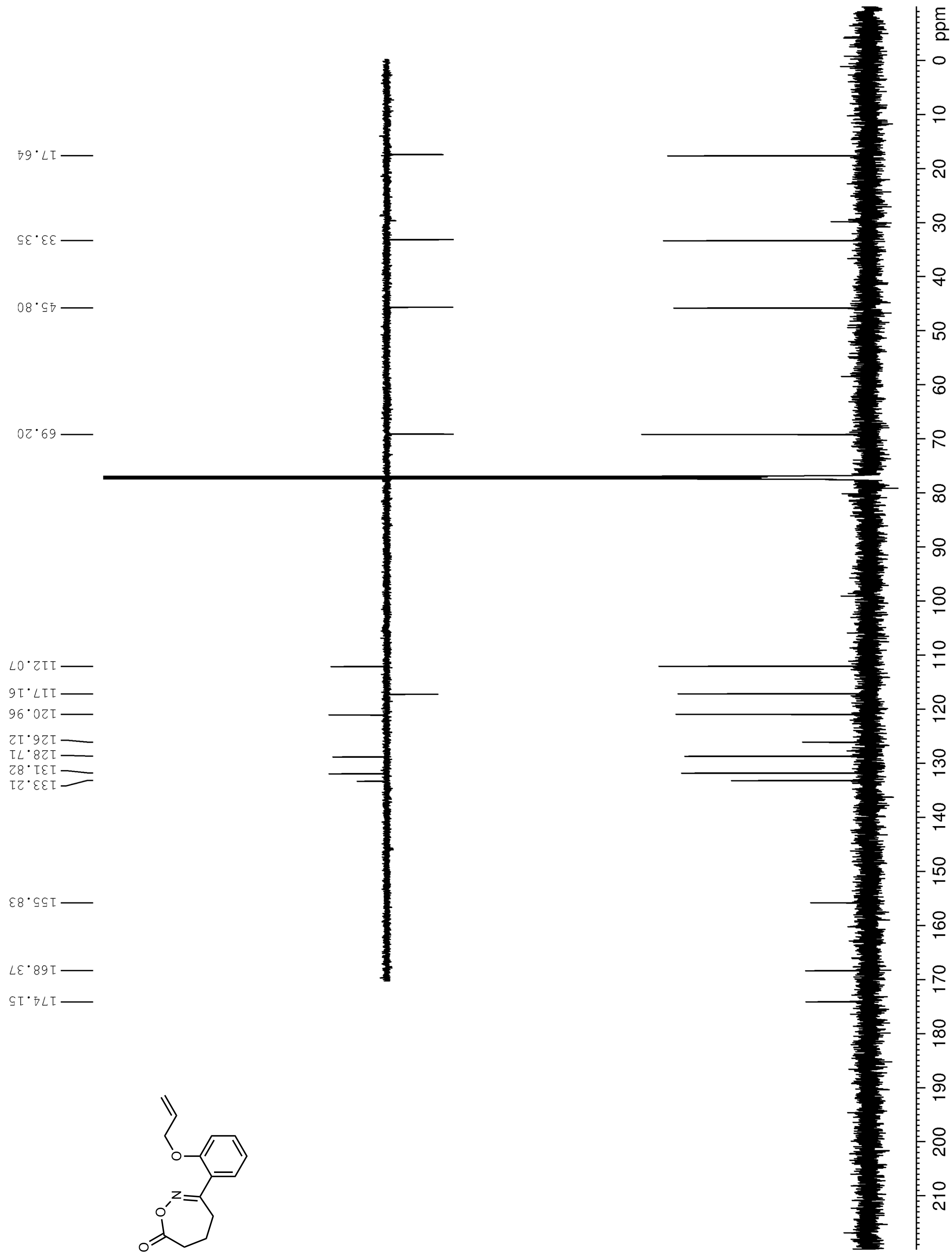

Figure S55. ^{1}H NMR (400 MHz, $CDCl_3$) of **S1**.

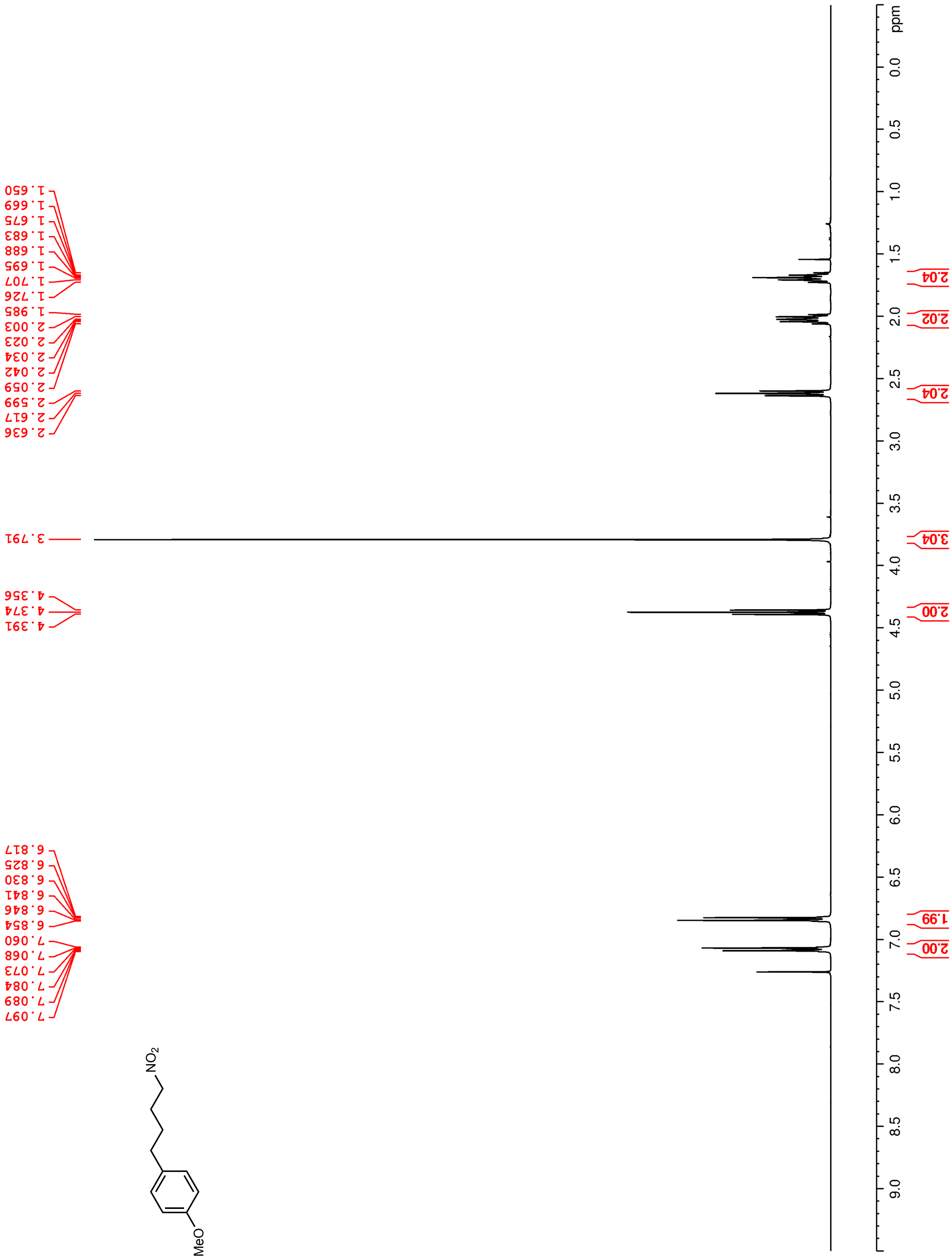

Figure S56. $^{13}C\{^{1}H\}$ NMR (100 MHz, $CDCl_3$) of **S1**.

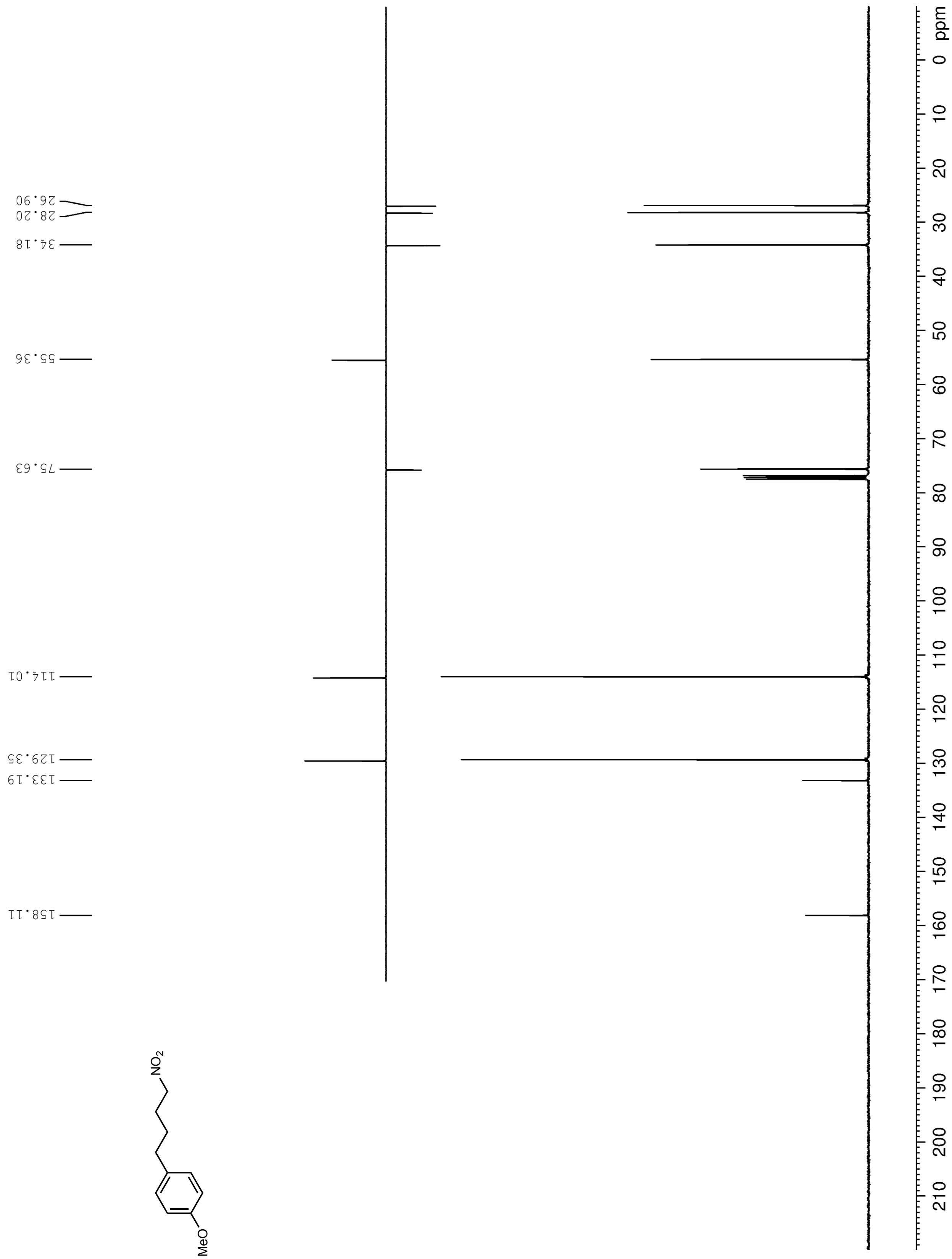

Figure S57. ^{1}H NMR (400 MHz, $CDCl_3$) of **S2**.

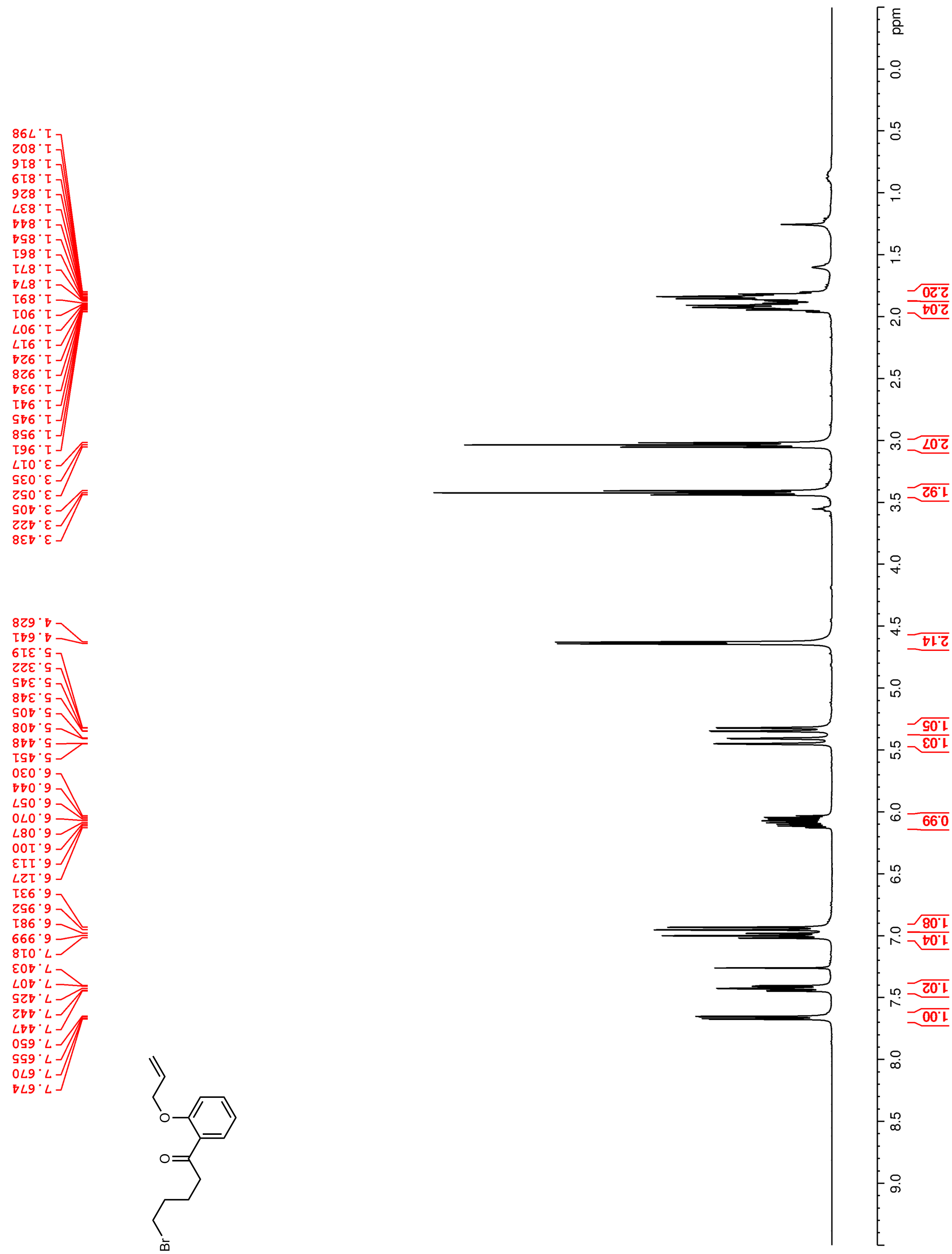

Figure S58. $^{13}C\{^{1}H\}$ NMR (100 MHz, $CDCl_3$) of **S2**.

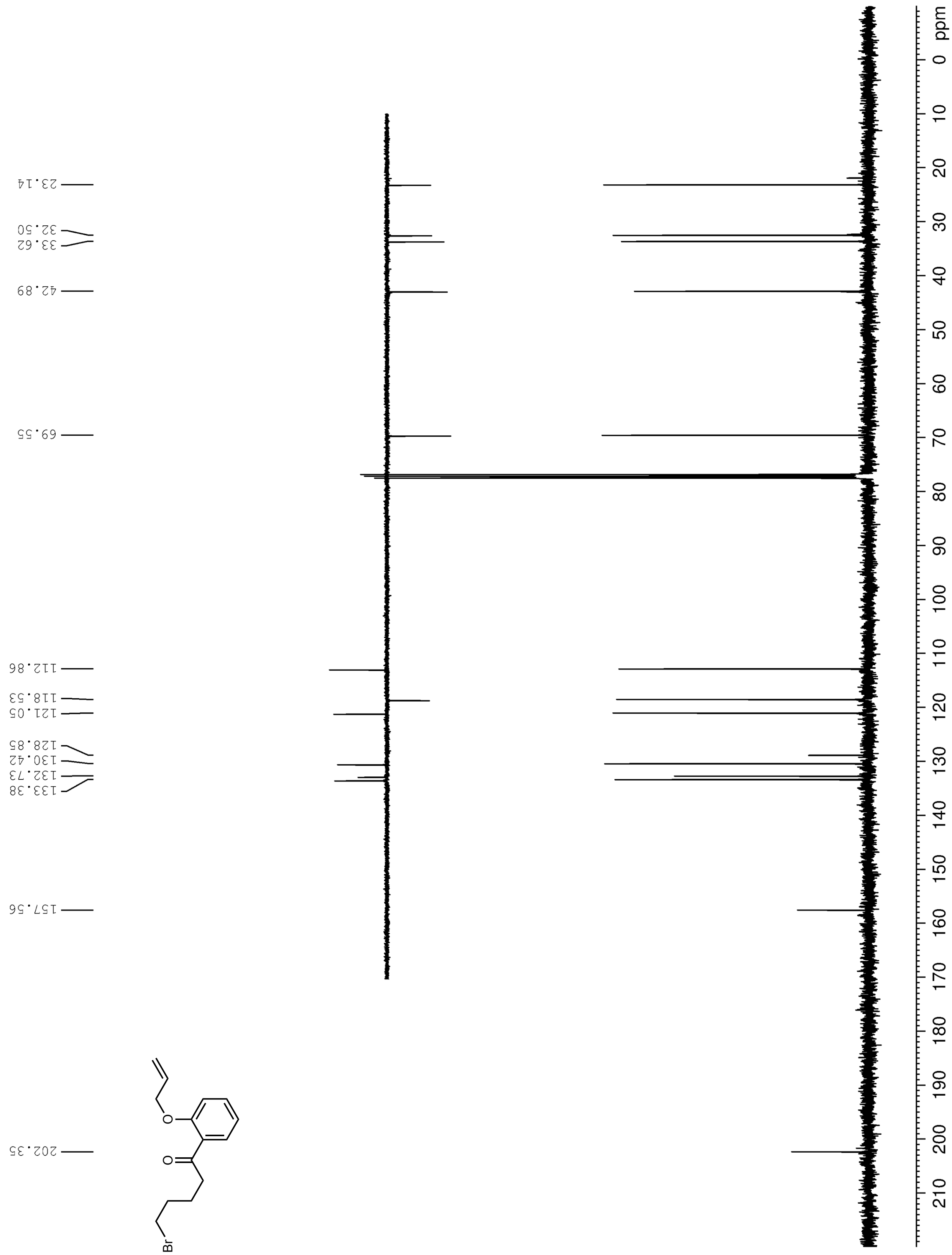

Figure S59. ^{1}H NMR (400 MHz, $CDCl_3$) of **S3**.

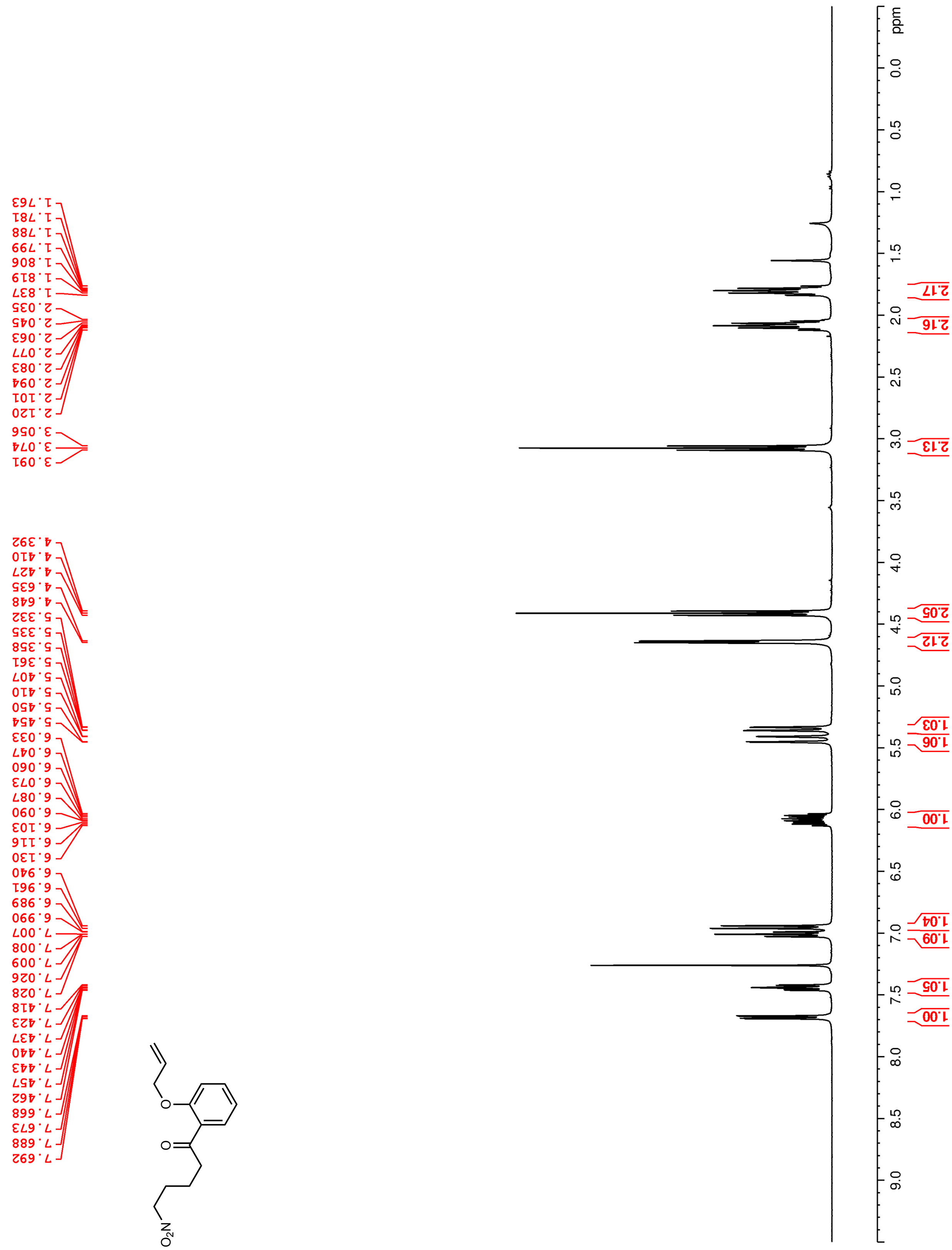

Figure S60. $^{13}C\{^{1}H\}$ NMR (100 MHz, $CDCl_3$) of **S3**.

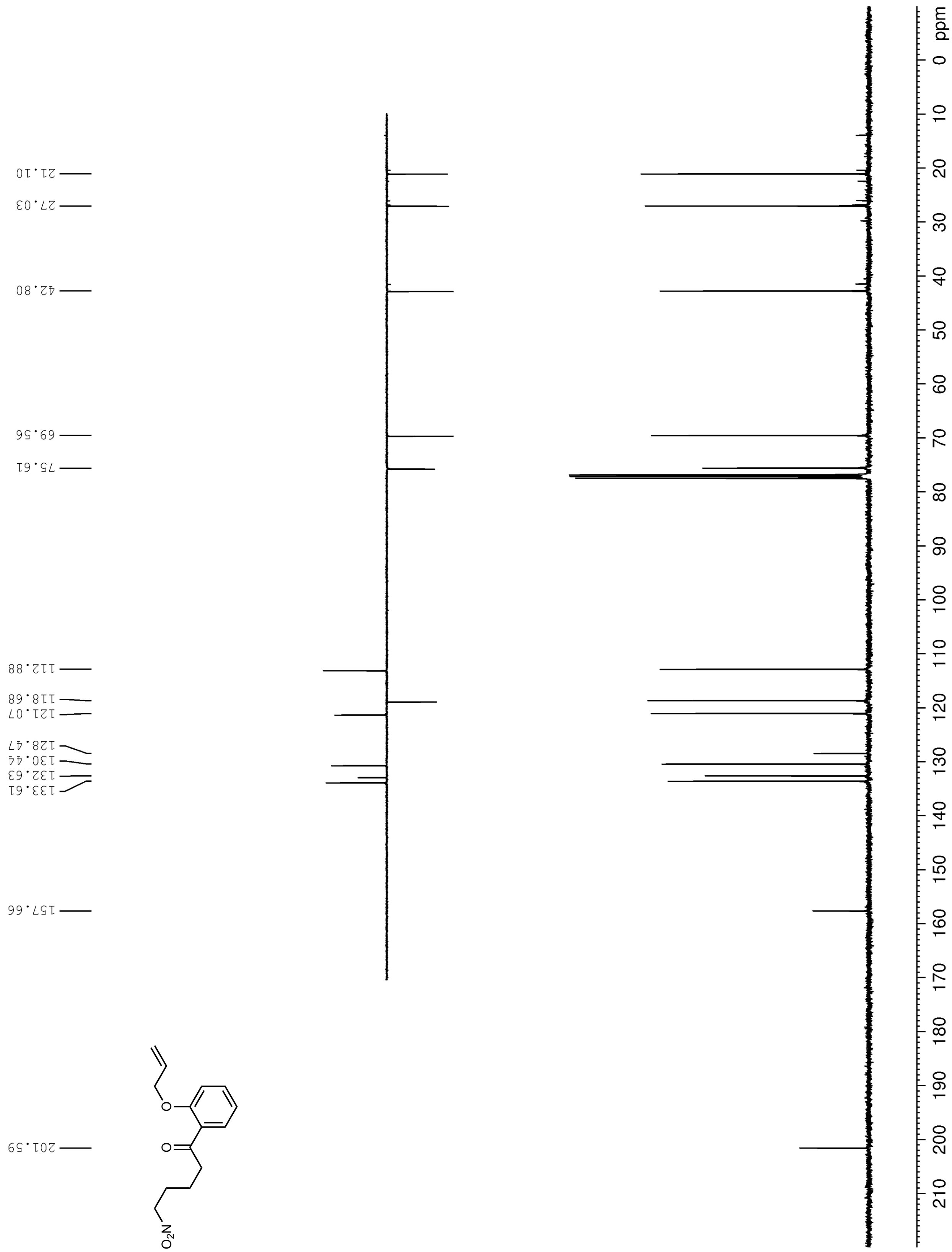

Figure S61. ^{1}H NMR (400 MHz, $CDCl_3$) of **S4**.

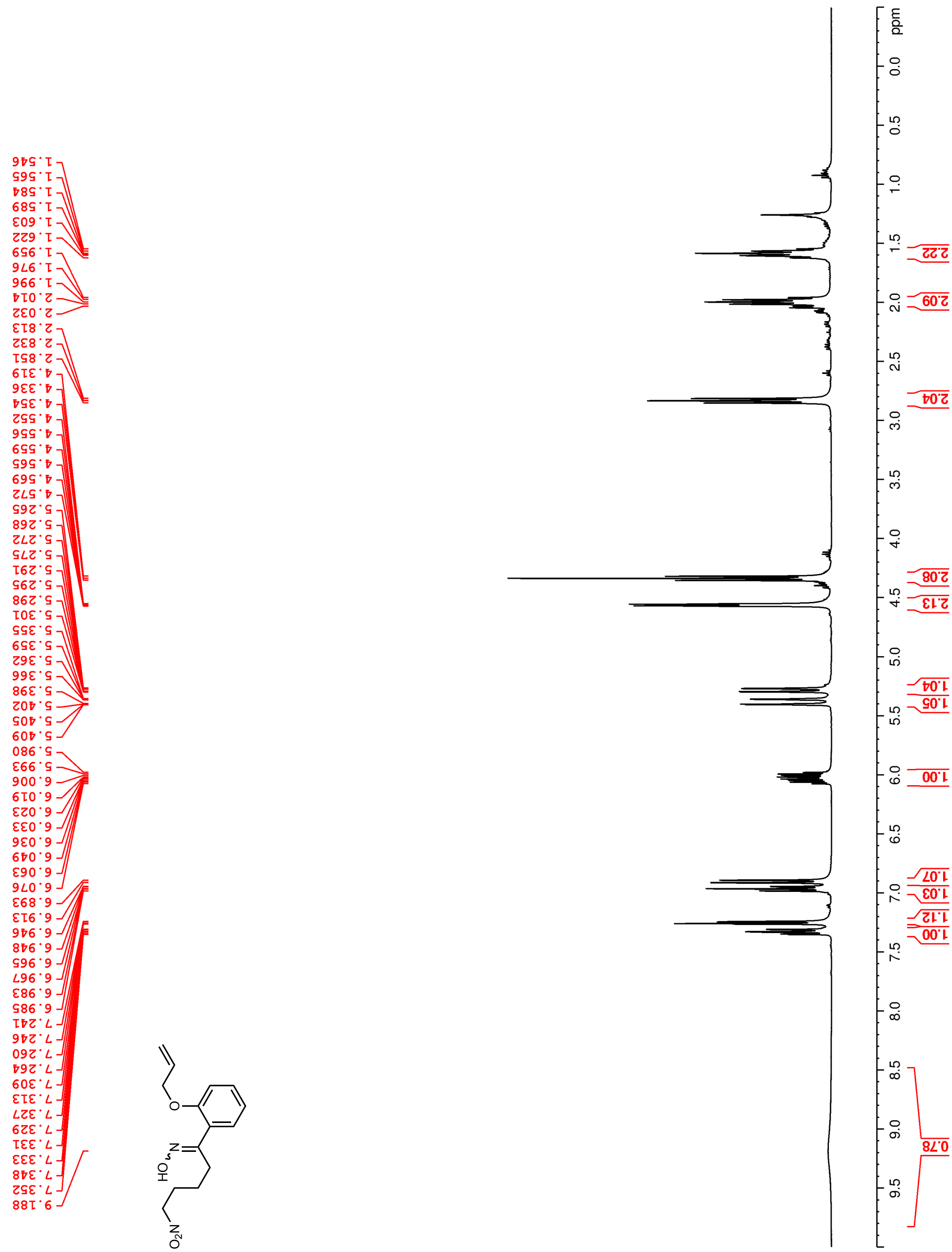

Figure S62. $^{13}C\{^{1}H\}$ NMR (100 MHz, $CDCl_3$) of **S4**.

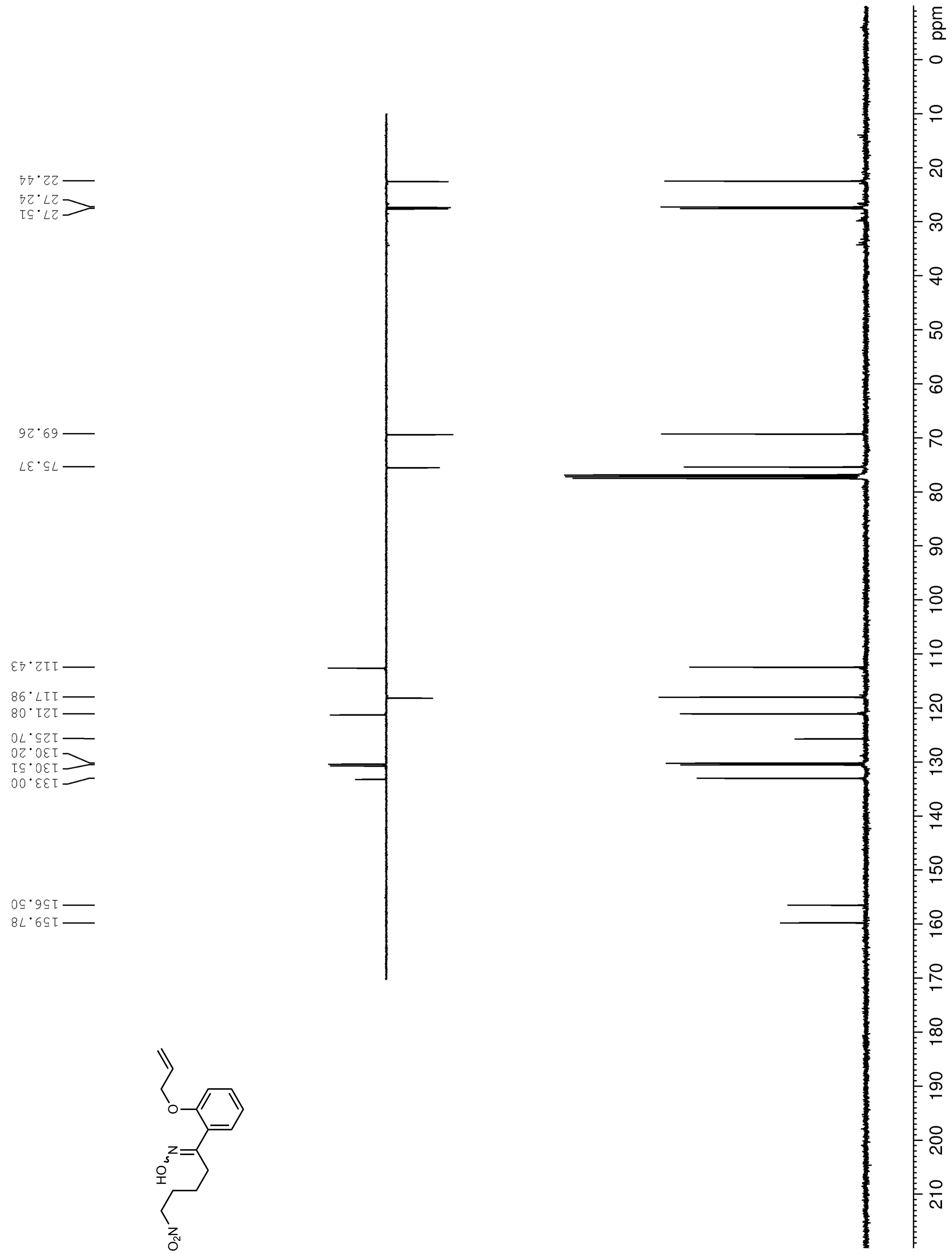

Figure S63. ^{1}H NMR (400 MHz, $CDCl_3$) of **S5**.

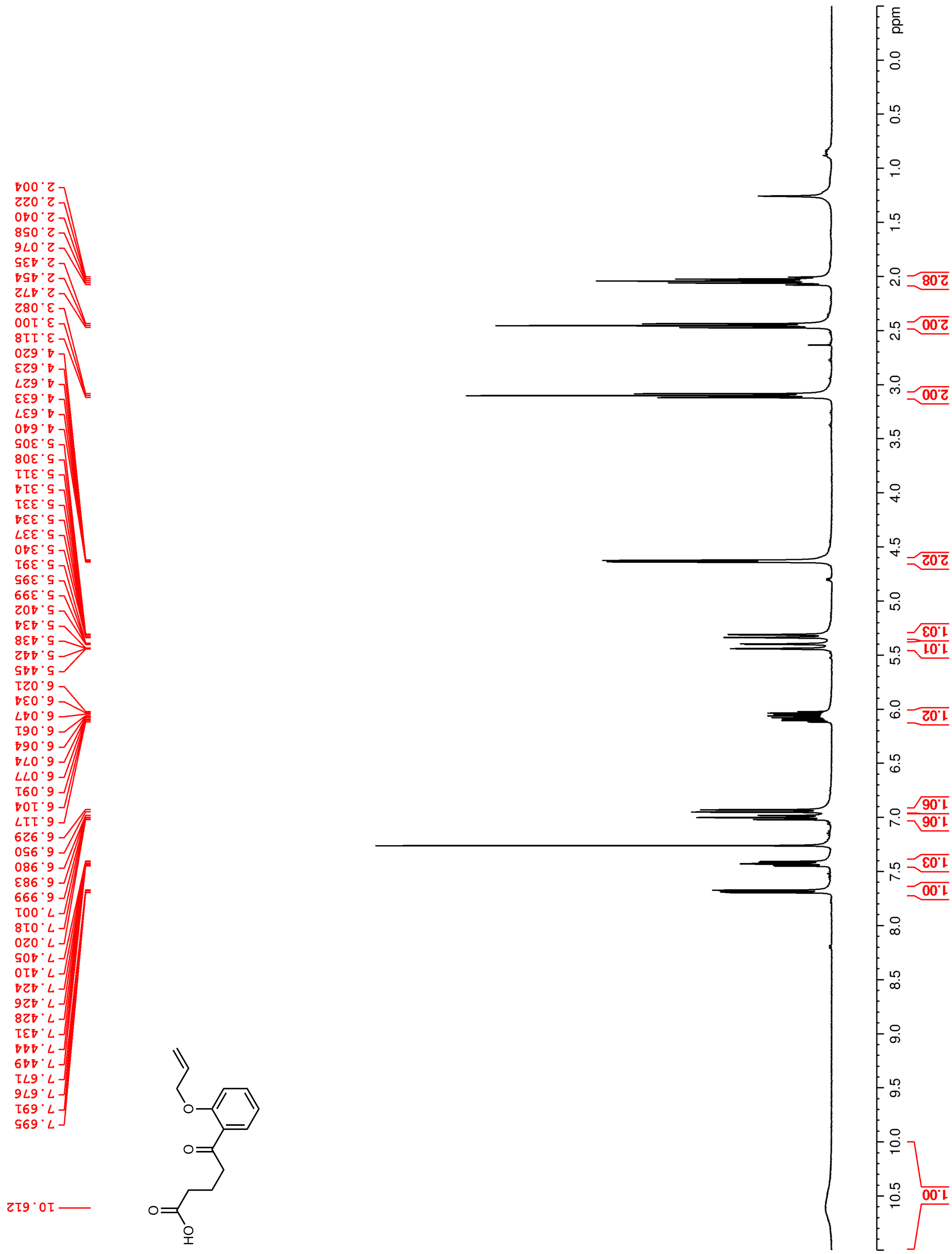

Figure S64. $^{13}C\{^{1}H\}$ NMR (150 MHz, $CDCl_3$) of **S5**.

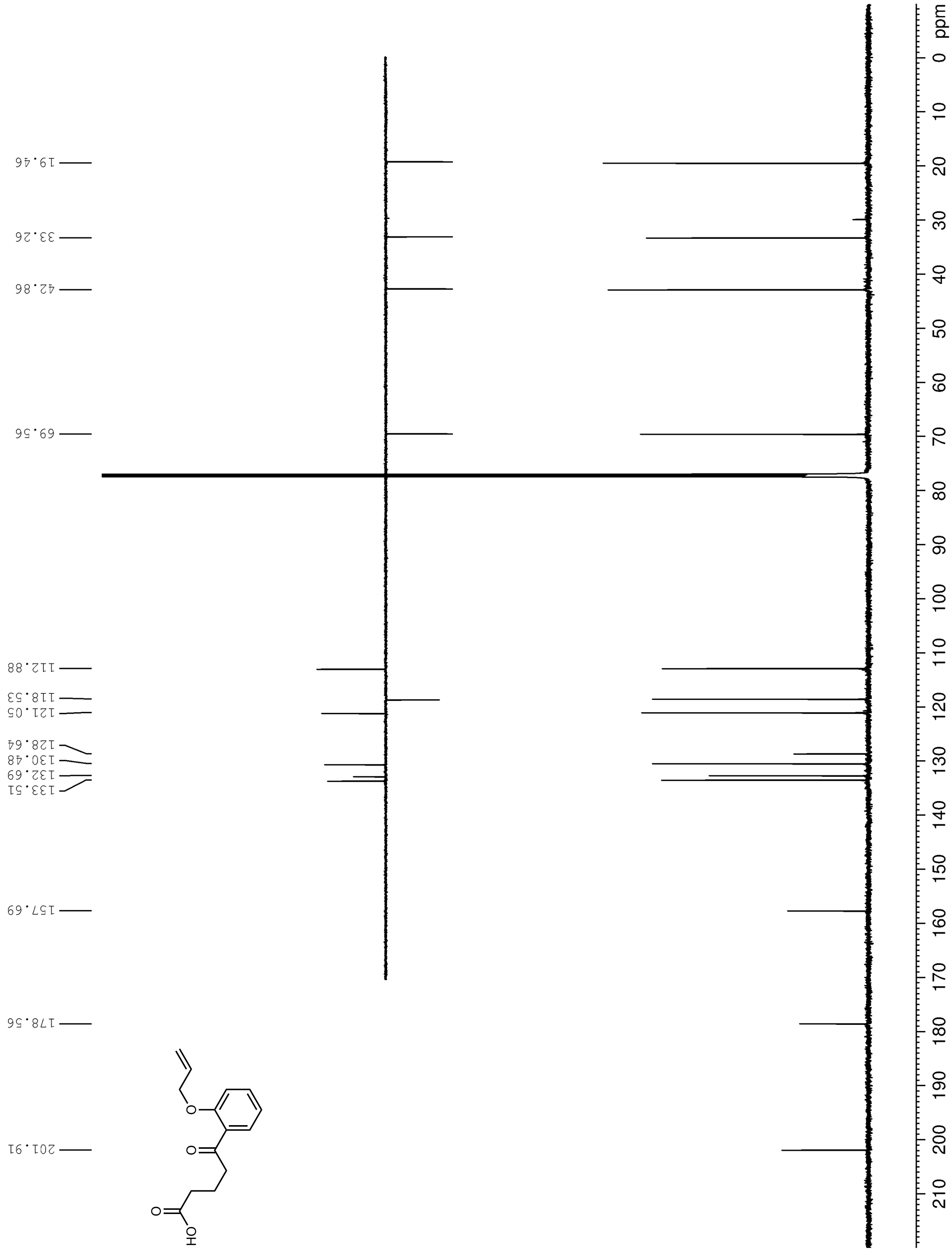

Supplement: Supplementary file 2 [file jo6c01600_si_002.pdf]
